# Significant Association Between XRCC1 Expression and Its rs25487 Polymorphism and Radiotherapy-Related Cancer Prognosis

**DOI:** 10.3389/fonc.2021.654784

**Published:** 2021-05-19

**Authors:** Li Gong, Ming Luo, Renhuang Sun, Li Qiu, Chunli Chen, Zhiguo Luo

**Affiliations:** ^1^ Department of Clinical Oncology, Taihe Hospital, Hubei University of Medicine, Shiyan, China; ^2^ Department of Pharmacology, School of Basic Medicine, Hubei University of Medicine, Shiyan, China

**Keywords:** radiation, side-effects, treatment response, overall survival, rs25487, XRCC1

## Abstract

**Background/Aims:**

XRCC1 (X-ray repair cross-complementing protein 1) expression and its single nucleotide polymorphism XRCC1 rs25487 (G>A) may be related to radiotherapy-related cancer prognosis or radiation-induced side effects. However, this association is controversial. We performed a bioinformatic analysis and a meta-analysis to obtain comprehensive results.

**Methods**

TCGA data sets and eligible publications published before November 31, 2020 were retrieved by searching the PubMed, Web of Science and CNKI (China National Knowledge Infrastructure) databases. ORs (odds ratios) and HRs (hazard ratios) with their corresponding 95% CIs (confidence intervals) were calculated to evaluate associations. For XRCC1 single nucleotide polymorphisms, we employed three types of comparisons: GA vs GG, AA vs GG and GA+AA vs GG.

**Results:**

Sixty nine articles with 10232 patients and 17 TCGA data sets with 2705 patients were included in the analysis. We observed that high XRCC1 expression was associated with an increased risk of minor treatment response and poor overall survival, XRCC1 rs25487 was associated with reduced risk of minor treatment response in esophageal cancer and an increased risk of high-grade side effects in head and neck cancer.

**Conclusion:**

The results suggest that XRCC1 expression and rs25487 polymorphism are prognostic factors for patients receiving radiotherapy-related treatment. Considering the insufficient treatment parameters provided and the various sample sizes in most of the studies, we suggest that genetic association studies related to radiation-based treatment should include more cancer types with sufficient statistical power and more detailed clinical parameters.

## Introduction

Radiotherapy using ionizing radiation is among the main treatments used to control or kill malignant neoplasms. Ionizing radiation functions by creating double-strand breaks (DSBs) or by damaging cell membranes, which can lead to cell death. However, the responses to radiotherapy vary between different cancer types, or even between cancer cells inside a tumor (1). Several factors are related to the responses of a specific tumor to radiotherapy: dose and fraction of radiation, and clinicopathologic characteristics including TNM stage (which is a notation system that employs alphanumeric codes to describes the stage of cancer that originates from a solid tumor with.), tumor size (2), and biological characteristics, such as pathological type, hypoxic state, DNA repair capacity, gene expression level and functional gene mutations. Normal tissue surrounding the tumor region or in the path of the radiation beams can be temporally injured during radiation treatment, lasting for a period after treatment, or even irrevocably damaged in some cases. Moreover, radiation might also have an impact on remote normal tissue. Patients treated with radiotherapy, even under the same treatment procedures, may experience a significant difference in radiation-induced early or late side effects, in terms of incidence and severity (3), which is a major challenge for radiotherapy practice. For patients, the major treatment response and slight/no side effects are important factors for long-term survival (4) and quality of life.

Since 2002, more than two-hundred published studies have reported an association between single nucleotide polymorphisms (SNPs) and cancer prognosis in patients receiving radiotherapy-related treatment. In the meantime, many studies have focused on the gene expression level, radiotherapy-related treatment response and cancer prognosis. According to our statistics, the key factors in evaluating prognosis mainly include survival (e.g. overall survival, progression-free survival), treatment response (e.g. complete remission, partial remission), and radiotherapy-related side effects (e.g. pneumonitis, esophagitis). One of the important goals of these studies is to identify genetic factors that can be used to predict radiotherapy-related cancer prognosis (5). Among these published studies, X-ray repair cross-complementing protein 1 (XRCC1) is one of the most studied genes and has been investigated for its possible association with cancer prognosis. The protein encoded by XRCC1, is involved in DNA repair with DNA ligase III and DNA polymerase (6). Preclinically, XRCC1 deficiency delays single-strand break rejoining, induces mutations and results in elevated levels of sister chromatid exchanges, a hallmark of genomic instability. XRCC1 deficiency results in hypersensitivity to ionizing radiation (7). Some clinical studies have indicated that a high XRCC1 expression level is associated with poor overall survival and treatment response in patients treated with radiotherapy (8, 9). Another study concludes that the XRCC1 expression level has no impact on overall survival or treatment response in rectal cancer patients treated with radiotherapy (10). The SNP rs25487 [Arg399Gln, G>A substitution at position 28152, exon 10, Arg to Gln (11)] is among the most widely researched XRCC1 SNPs, and yet researchers still do not agree on its impact. A number of studies with small sample sizes have investigated the association between this SNP and response to chemoradiotherapy (12–15). However, the results are not consistent. A previous meta-analysis of four studies has stated that this SNP does not predict response to chemoradiotherapy (n = 511) in patients with rectal cancer (16). However, the sample size in genetic association studies is still too small to obtain sufficient power to a robust conclusion.

To address the issues of inconsistent conclusions and limited sample size, we performed meta-analyses that included the most comprehensive literature and obtained the largest sample size for this topic to date. To minimize the effect of publication bias, we collected data from published articles (including published Ph.D. and master’s theses) on the association between XRCC1 expression level and radiotherapy-related treatment response/cancer prognosis; we also explored the relationship between XRCC1 rs25487 polymorphism and radiotherapy-related treatment response/radiation-induced side effects.

## Methods

### Literature Search

Eligible publications were retrieved by searching the PubMed, Web of Science and Chinese National Knowledge Infrastructure (CNKI) databases up to November 31, 2020. The search strategy was based on the following keywords: [(X-ray Repair Cross Complementing Protein 1(MeSH Terms)] AND Radiotherapy [MeSH Terms]) AND Neoplasms [MeSH Terms]. Studies focusing on the association between XRCC1 expression/XRCC1 rs25487 and radiotherapy-related cancer prognosis were screened for further analysis.

### Inclusion Criteria and Exclusion Criteria

Two independent reviewers performed the inclusion assessment. The inclusion criteria were as follows: (1) an independent case-control or cohort study; (2) evaluation of the association between the XRCC1 expression level and radiotherapy-related treatment response/cancer prognosis; (3)evaluation of the association between XRCC1 rs25487 and radiotherapy-related treatment response/radiation-induced side effects in patients receiving radiotherapy-related treatment; (4) sufficient data on the relationship between XRCC1 expression and overall survival, with estimated hazard ratios (HRs) and 95% confidence intervals(CIs) determined by multivariate analysis and reported in the articles or available to be indirectly calculated *via* Kaplan Meier (K-M) curves; and (5) provision of the number of patients in the high/low XRCC1 expression group or with different genotypes in the case-control group.

Exclusion criteria: (1) studies without sufficient data associated with radiotherapy-related treatment response, overall survival (OS), or radiation-induced side effects, (2) duplicated publications, (3) studies based on animal models or cell lines, (4) other literature types: reviews, letters, abstracts, meta-analysis, case reports, etc.

## Data Extraction and Quality Assessment

Two independent reviewers collected data from the studies included in the meta-analysis. The following data were collected: first author, publication year, cancer type, treatment, side effects, acute/late degree, evaluation criteria, cutoff value, follow-up period, survival outcome, HR (95% CI), number of patients in the high/positive XRCC1 expression group and low/negative XRCC1 expression group associated with treatment response, and number of patients in the case-control group and the association between XRCC1 rs25487 and treatment response/side effects. If survival rates were not obtained from multivariate analysis, the survival HR (95% CI) was indirectly retrieved from K-M curves using Engauge Digitizer software. This method was described in detail by Tierney et al. (17). A calculation spreadsheet was prepared in Microsoft Excel to obtain the observed minus expected events (O-E), the variance (V), the HR, the log (HR), and its standard error (SE) for each of the individual trials. Newcastle-Ottawa Scale (NOS) criteria were used to assess the quality of studies. NOS score ≥ 6 were considered high-quality studies, otherwise, the studies were considered as low-quality.

### Validation of TCGA Database

The Cancer Genome Atlas (TCGA) database was searched to further verify the relationship between XRCC1 expression and radiotherapy-related cancer prognosis. The patients were filtered out if they had not undergone radiotherapy and/or had no survival information. Overall survival was assessed using R software (version 4.0.3).

### Subgroup Analysis

To reduce the effect of specific parameters (e.g. cancer type, side effects, treatment, acute/late degree and cutoff value) on the association between rs25487 and treatment/side effects, we performed subgroup analysis if there were five or more studies. When we performed subgroup analysis for cancer, we classified by the type of tissue and the primary site. In treatment response, the patients in each study were divided into two groups according to their treatment response: minor treatment response (case group) and major treatment response (control group). Major treatment response refers to a complete/partial response (complete response: disappearance of all known disease, confirmed at 4 weeks; partial response ≥50% decrease, confirmed at 4 weeks.) or grade <3 regression grade (grade 1: absence of residual cancer and extensive fibrosis, grade 2: rare residual cancer cells scattered through the fibrosis), and minor treatment response refers to stable/progressive disease (stable disease: neither partial response nor complete response criteria meet, progressive disease: ≥25% increase, no partial response or complete response, stable disease documented before increased disease, new lesion(s), or a ≥25% increase in one lesion) or a ≥3 regression grade (grade 3: increased residual cancer cells but fibrosis still predominating; grade 4: residual cancer outgrowing fibrosis, grade 5: an absence of regressive changes). We classified side effects by organ system and clinical dissection/irradiation region. Cystitis and proctitis were classified as bladder and/or rectal toxicity. Digestive system toxicity included dysphagia and radiation esophagitis. Anemia, leukocytopenia, neutropenia and thrombocytopenia were classified as hematological toxicity. Skin toxicity included radiation dermatitis and erythema. Telangiectasia, fibrosis or fat necrosis were classified as soft tissue injury.

### Statistical Analysis

HRs and ORs, with their corresponding 95% CIs, were utilized to analyze the association between XRCC1 expression and prognostic indicators (OS) and treatment response, respectively. ORs and 95%CIs were also calculated to evaluate the association between XRCC1 rs25487 and treatment response/side effects. For XRCC1 rs25487 analysis, we used three genetic models to combine data: heterozygote model (GA vs GG), homozygote model (AA vs GG), and dominant model (GA+AA vs GG). A chi-squared test and Higgins’s (I^2^) test were used to assess heterogeneity. If I^2^ <50%, the fixed effect model was chosen to combine data, otherwise the random effect model would be adopted. In the sensitivity analysis, the “metaninf” module was used to investigate the influence of each individual study on the overall meta-analysis summary estimate by omitting each study in turn. Begg’s rank correlation test and Egger’s linear regression test were chosen to assess publication bias and P<0.05 was considered statistically significant. All statistical analyses were performed using STATA 14.0 software (StataCorp. 2015. Stata Statistical Software: Release 14. College Station, TX: StataCorp LP.).

## Results

### Literature Search and Study Characteristics

**Figure 1:**
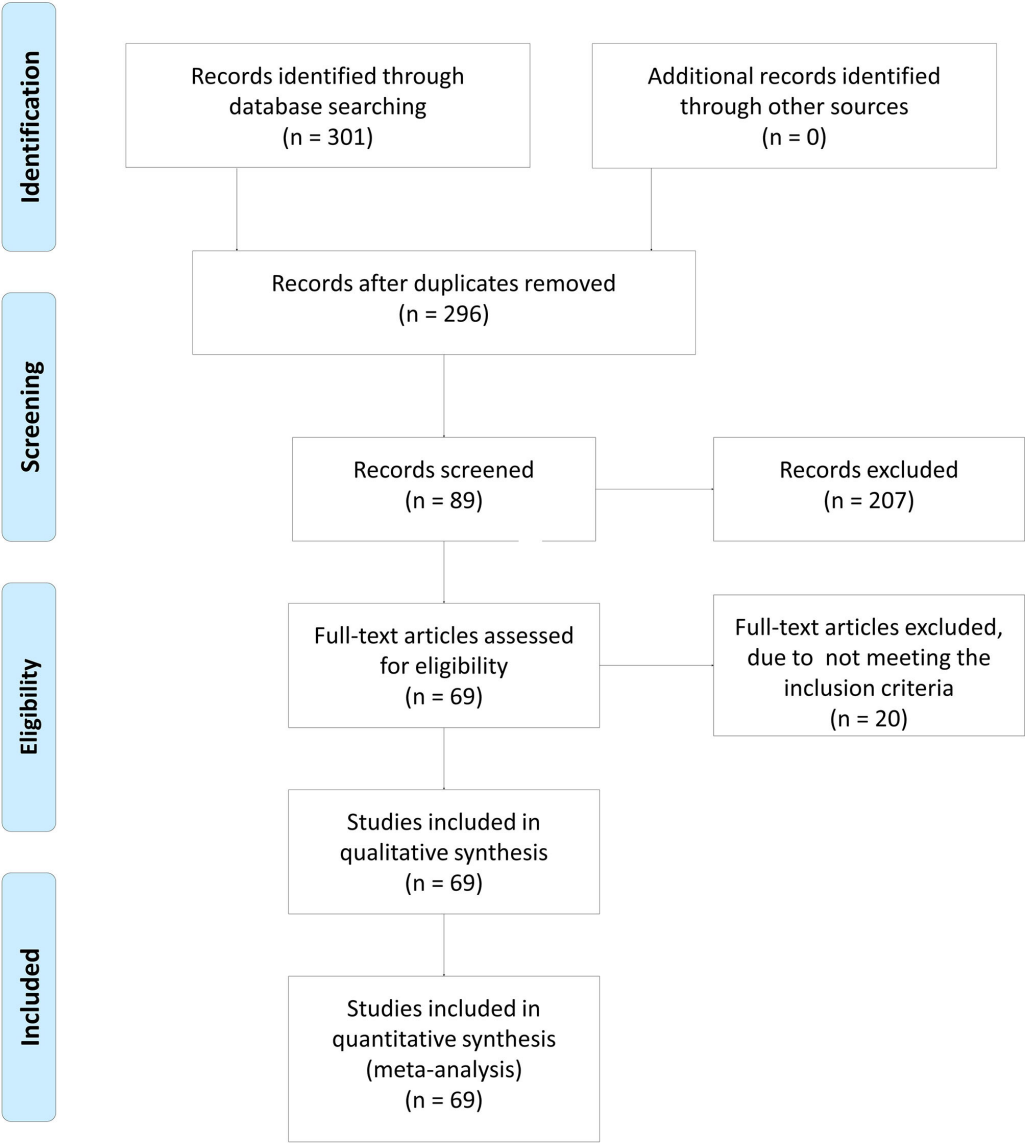
The flow diagram of study inclusion. In total, 301 articles were identified using the searching strategy. Of these, 207 articles were excluded because they do not report the association. Then, five articles were excluded due to duplication. Finally, 69 articles were included in meta-analysis. The flow diagram of study inclusion was cited from Moher et al. (18).

**Figure 1** shows the flow diagram of the study selection process. In total, 301 articles were identified using the search strategy. Of these, 207 articles were excluded due to irrelevancy. Another five articles were excluded due to duplication. Finally, 69 articles were included in the meta-analysis (8–10, 13–15, 19–79). The articles were allocated into two parts: nine articles included XRCC1 expression and treatment response/OS data, of which seven articles reported treatment response data, six articles reported OS data, and four articles reported both treatment response and OS data. Patients in these studies were divided into high/positive and low/negative groups. 60 articles included XRCC1 rs25487 and treatment response/side effects data, in which 24 articles reported treatment response data, 40 articles reported side effects data, and four articles reported both treatment response and side effects data. Patients in these studies were divided into case and control groups. All studies scored ≥6 on the NOS, which indicated that all studies were of high quality. **Tables 1** and **2**, a partial list of the characteristics of the included articles is presented. In total, 10,232 patients were included in the meta-analysis. **Supplementary Table 1** shows the full characteristics of these articles.

**Table 1:** Characteristics of studies associated with XRCC1 expression and prognosis.

Author (Ref)	Cancer	Treatment	Prognosis type	Sample size	Cutoff value	HR (95% CI)	OR (95% CI)	NOS
Liu et al. (58)	ESCC	RT	Treatment response	59	10%	/	0.32 (0.06–1.74)	8
Zhao and Yu (9)	NSCLC	RT	Treatment response	62	50%	/	0.34 (0.10–1.14)	6
Ang et al. (8)	HNSC	RCT+Surgery	OS	68	8 score	6.02 (2.36–15.37)	/	8
Sakano et al. (59)	Bladder cancer	RCT	Treatment response	142	H-score ≥1.0	/	0.97 (0.48–1.96)	8
Ge et al. (60)	ESCC	RCT+Surgery	OS	44	2–6 scores	1.09 (0.43–2.77)	/	8
Zheng (61)	ESCC	RT	Treatment response and OS	76	>4 scores	1.48 (0.84–2.60)	0.53 (0.19–1.47)	7
Geng (62)	Gastric cancer	RT	Treatment response and OS	46	NM	1.45 (0.36–6.96)	0.11 (0.02–0.57)	8
Huang et al. (10)	Rectal cancer	RCT+Surgery	Treatment response and OS	86	50%	1.80 (0.48–6.82)	0.54 (0.21–1.38)	8
Zhang et al. (63)	ESCC	RT	Treatment response and OS	76	2–6 scores	1.08 (0.59–1.99)	0.47 (0.18–1.23)	8

RT, Radiotherapy; RCT, Radio-chemotherapy; OS, Overall survival; NM, Not mentioned; ESCC, Esophageal squamous cell carcinoma; NSCLC, Non-small cell lung cancer; HNSC, Head and neck squamous cancer.

**Table 2:** Characteristics of studies associated with XRCC1 rs25487 and prognosis.

Author (Ref)	Cancer	Treatment ^1^	Prognosis type ^2^	Number of Patients	NOS
Sakano et al. (54)	Bladder cancer	RCT	Treatment response	72	8
Qing-hua et al. (55)	NSCLC	RCT	Treatment response	120	6
Warnecke-Eberz et al. (56)	ESCA	RCT	Treatment response	50	8
Xu-sheng et al. (57)	ESCC	RT	Treatment response	94	7
Grimminger et al. (14)	Rectal cancer	RCT	Treatment response	81	7
Lamas et al. (15)	Rectal cancer	RCT	Treatment response	93	8
Balboa et al. (13)	Rectal cancer	RCT	Treatment response	65	8
Cecchin et al. (12)	Rectal cancer	RCT	Treatment response	235	8
Yoon et al. (41)	EAC	RCT	Treatment response	60	6
Paez et al. (72)	Rectal cancer	RCT	Treatment response	126	8
Zha et al. (73)	NSCLC	RCT	Treatment response	52	6
Huang et al. (74)	ESCC	RT	Treatment response	150	8
Fan et al. (75)	CESC	RT	Treatment response	73	7
Chen et al. (76)	NSCLC	RT	Treatment response	60	8
Yu et al. (77)	ESCC	RCT	Treatment response	73	8
Wu (49)	NPC	RT+RCT	Treatment response and side effects	114	7
Zhai et al. (39)	NPC	RCT	Treatment response and side effects	60	8
Huang et al. (78)	ESCC	RCT	Treatment response	50	8
Sun et al. (79)	ESCC	RT+RCT	Treatment response	97	6
Wang et al. (65)	NPC	RT+RCT	Treatment response and side effects	174	7
Zhang et al. (66)	NPC	RCT	Treatment response	100	8
Zhang (69)	Rectal cancer	RCT	Treatment response	55	8
Nicosia et al. (68)	Rectal cancer	RCT	Treatment response	80	8
Yang and Liu (71)	NSCLC	RT	Treatment response and side effects	486	6
Moullan et al. (19)	Breast cancer	RT	Side effects	254	6
Chang-Claude et al. (20)	Breast cancer	RT	Side effects	446	7
Giotopoulos et al. (21)	Breast cancer	RCT	Side effects	82	6
Suga et al. (22)	Breast cancer	RT	Side effects	389	6
Alsbeih et al. (23)	NPC	RT+RCT	Side effects	50	7
Burri et al. (24)	Prostate adenocarcinoma	RT+(RT+HT)	Side effects	135	7
Falvo et al. (25)	Breast adenocarcinoma	RCT+(RT+HT)	Side effects	403	7
Chang-Claude et al. (26)	Breast cancer	RT	Side effects	403	7
Popanda et al. (27)	Prostate cancer	RT	Side effects	405	6
Zschenker et al. (28)	Breast cancer	RT+RCT+(RT+HT)+(RCT+HT)	Side effects	69	7
Alsbeih et al. (29)	NPC	RT+RCT	Side effects	60	8
Mangoni et al. (30)	Breast cancer	RT+RCT	Side effects	87	6
Zhou et al. (31)	Breast cancer	RT	Side effects	171	6
Sakano et al. (32)	Bladder cancer	RCT	Side effects	95	6
Ishikawa et al. (33)	Cervical cancer	RT	Side effects	208	8
Yin et al. (34)	NPC	RT+RCT+(RT+Other)	Side effects	165	8
Pratesi et al. (35)	HNSC	RCT	Side effects	101	6
Langsenlehner et al. (36)	Prostate cancer	RT+(RT+HT)	Side effects	575	8
Terrazzino et al. (37)	Breast cancer	RT	Side effects	237	7
Yoon et al. (41)	EAC	RCT	Side effects	60	7
Raabe et al. (40)	Breast cancer	RT+(RCT+HT)	Side effects	83	6
Terrazzino et al. (38)	Breast cancer	RT+RCT+(RT+HT) +(RCT+HT)	Side effects	285	6
Li et al. (42)	NPC	RT+RCT	Side effects	114	8
Duldulao et al. (43)	Rectal cancer	RCT	Side effects	347	6
Tucker et al. (44)	NSCLC	RT+RCT	Side effects	169	6
Zhu (45)	ESCC	RT+RCT	Side effects	182	7
Cheuk et al. (46)	NPC	RT+RCT	Side effects	120	6
Venkatesh et al. (47)	Head and neck cancer	RT+RCT	Side effects	166	6
Alsbeih et al. (48)	NPC	RT+RCT	Side effects	155	7
Chen et al. (50)	NSCLC	RT	Side effects	60	7
Lan et al. (51)	Cervical cancer	RT	Side effects	152	8
Mumbrekar et al. (52)	Breast cancer	RT+RCT	Side effects	119	8
Smith et al. (53)	Rectal cancer	RCT	Side effects	165	7
Chen et al. (64)	NPC	RCT	Side effects	114	7
Du et al. (67)	Lung cancer	RT	Side effects	149	8
Xie et al. (70)	NSCLC	RCT	Side effects	178	8

RT, radiotherapy; RCT, radio-chemotherapy; HT, hormonal therapy; NSCLC, non-small cell lung cancer; ESCA, esophageal carcinoma; ESCC, esophageal squamous cell carcinoma; EAC, esophageal adenocarcinoma; CESC, cervical squamous cell carcinoma; NPC, nasopharyngeal cancer; HNSC, head and neck squamous cell carcinoma.

### Association Between XRCC1 Expression and Treatment Response/OS

#### Overall Analysis

Seven studies reported data showing patient treatment response. The pooled OR indicated that high XRCC1 expression was closely related to an increased risk of minor treatment response (OR = 0.52, 95% CI: 0.36–0.76, P = 0.001). A heterogeneity test showed low heterogeneity among these studies (*I^2^* = 17.9%, P = 0.294). The association between XRCC1 and OS was assayed using data from six studies, and we found that high XRCC1 expression was significantly related to poor OS (HR = 1.67, 95% CI: 1.00–2.78, P = 0.048), However, heterogeneity was found to be relatively large (*I^2^* = 50.2%, P = 0.074). **Supplementary Files 1** and **2** show the forest plots of overall analysis of the association between XRCC1 expression and treatment response and OS, respectively.

#### Subgroup Analysis

In treatment response analysis, high XRCC1 expression was associated with increased risk of minor treatment response in esophageal cancer (OR = 0.46, 95% CI: 0.24–0.88, P = 0.019) and in patients receiving radiotherapy only (OR = 0.36, 95% CI: 0.21–0.62, P = 0.000). In OS analysis, we found no relation between XRCC1 expression and OS in the cancer subgroup or treatment subgroup. **Supplement Files 1** and **2** show the forest plots of subgroups with regard to treatment response and OS, respectively.

#### Sensitivity Analysis and Publication Bias

To assess whether the combined results were affected by a single study, sensitivity analysis was performed by a single study by calculating the results when individual studies were omitted and determining if the result was within the CI. The results are shown in **Supplementary Files 5**, indicating that the results were robust and reliable. To evaluate publication bias, Begg’s test and Egger’s test were conducted, and we found publication bias in the analysis between XRCC1 expression and treatment response (Begg’s test P = 0.035, Egger’s test P = 0.004). No significant publication bias was detected for OS (Begg’s test P = 0.452, Egger’s test P = 0.554). **Supplementary File 8** show the funnel plots of treatment response and OS.

#### Validation of TCGA Data Set Results

**Figure 2:**
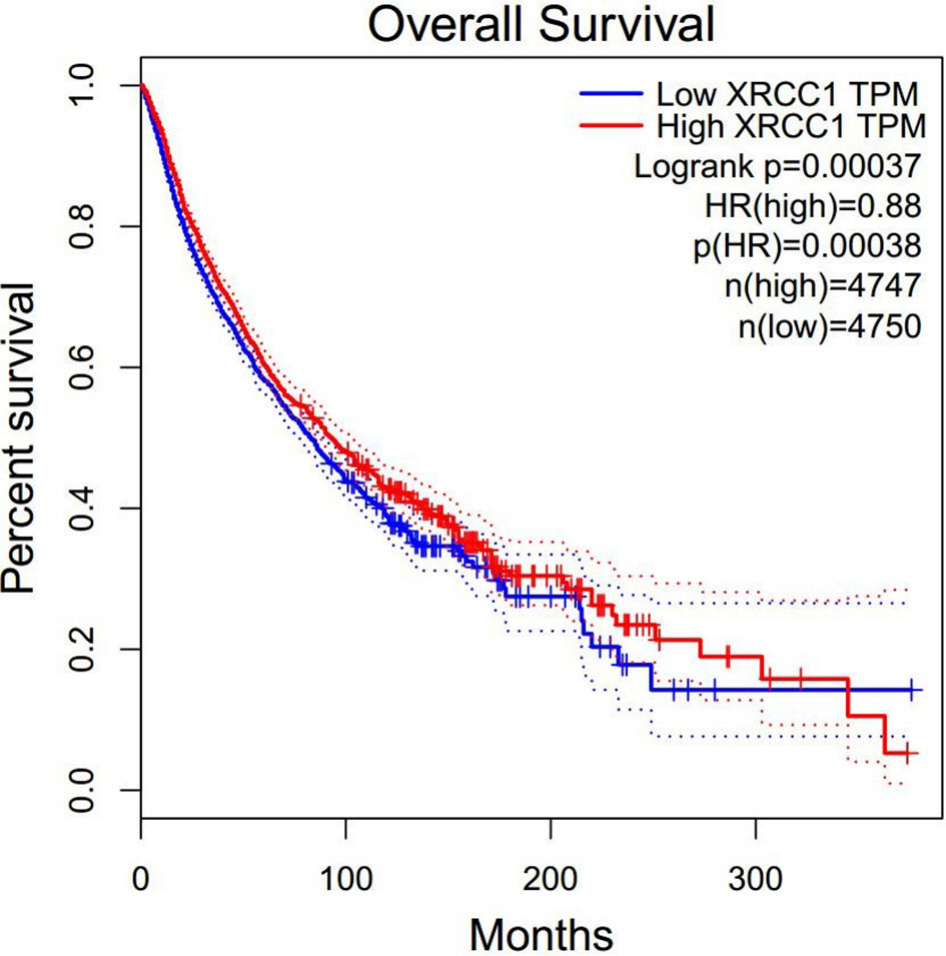
Overall survival of XRCC1 expression in TCAG data set.

To further explore the prognostic value of XRCC1 expression in cancer patients who received radiotherapy, we retrieved expression data for radiotherapy-related cancer prognosis in all cancer types from TCGA data set. A total of 2705 patients with 17 cancer types, consisting of digestive, respiratory, urinary, female reproductive, head and neck, neurological, urinary, and soft tissue system cancers, were included in the analysis. The patients were divided into high- and low- XRCC1 expression groups according to the median XRCC1 expression value. Meta-analysis of all the studies indicated that high XRCC1 expression may be related to poor OS; however, the result was not statistically significant (HR = 1.08, 95% CI: 0.93–1.25, P = 0.329). **Supplement File 2** show the forest plots of the result. We also explored the prognostic value of XRCC1 expression in cancer patients regardless of treatment. Surprisingly, the results showed that high XRCC1 expression was significantly associated with better OS (HR = 0.88, 95% CI: 0.82–0.94, P = 0.00038), which was contrary to our results related to radiotherapy (**Figure 2**).

### Association Between XRCC1 rs25487 and Treatment Response/Side Effects

#### Overall Analysis

No significant association was found between rs25487 and treatment response (**Table 3**). In the side effects analysis, increased risks were found to be associated with the GA genotype (OR = 1.67, 95% CI: 1.05–1.29, P = 0.004) and GA+AA genotype (OR = 1.18, 95% CI: 1.07–1.30, P = 0.001) compared with GG genotype (**Table 4**). **Supplementary Files 3** and **4** show the forest plots of overall and subgroup analyses for treatment response and side effects, respectively.

**Table 3:** Overall and subgroup analysis for relation between XRCC1 rs25487 and treatment response.

Comparison	Group	Subgroup	No. Patients	No. Study	OR	95% CI	P value	Heterogeneity	Effect model
AA vs GG	Overall		1396	19	0.89	0.53–1.49	0.658	61.0%	Random
	Cancer	Esophageal cancer	345	6	0.65	0.15–2.73	0.554	80.9%	Random
		Rectal cancer	383	6	0.63	0.39–1.01	0.056	0.0%	Fixed
	Cutoff	SD+PD	737	8	0.97	0.41–2.30	0.626	67.2%	Random
		**Grade ≥3**	**325**	**5**	**0.52**	**0.31**–**0.86**	**0.012**	**0.0%**	**Fixed**
	Treatment	RCT	688	12	0.69	0.47–1.01	0.059	20.2%	Fixed
		RT	557	5	1.30	0.36–4.63	0.687	84.7%	Random
GA vs GG	Overall		1843	19	1.14	0.93–1.40	0.221	19.1%	Fixed
	Cancer	**Esophageal cancer**	375	**6**	**0.50**	**0.30**–**0.83**	**0.008**	**15.5%**	**Fixed**
		Rectal cancer	537	6	1.41	0.99–2.01	0.059	0.0%	Fixed
	Cutoff	SD+PD	983	8	1.06	0.81–1.38	0.673	48.3%	Fixed
		Grade ≥3	388	5	1.20	0.78–1.86	0.407	6.1%	Fixed
	Treatment	RCT	945	12	1.19	0.90–1.59	0.222	0.0%	Fixed
		RT	683	5	0.92	0.46–1.86	0.819	60.7%	Random
GA+AA vs GG	Overall		2580	24	1.08	0.90–1.29	0.401	48.8%	Fixed
	Cancer	Esophageal cancer	573	7	0.71	0.29–1.77	0.464	76.6%	Random
		Rectal cancer	742	7	1.05	0.77–1.44	0.743	0.0%	Fixed
	Cutoff	SD+PD	1223	9	0.85	0.51–1.43	0.536	68.4%	Random
		Grade ≥3	586	6	0.85	0.59–1.21	0.360	0.0%	Fixed
	Treatment	RCT	1393	16	1.03	0.81–1.30	0.817	14.0%	Fixed
		RT	963	6	0.99	0.46–2.14	0.975	78.4%	Random

RCT, radio-chemotherapy; RT, radiotherapy; SD, stable disease; PD, progressive disease. The bold values mean that they are statistically significant.

**Table 4:** Overall and subgroup analysis for relation between XRCC1 rs25487 and side effects.

Comparison	Group	Subgroup	No. Patients	No. Study	OR	95% CI	P value	Heterogeneity	Effect model
AA vs GG	Overall		5333	57	1.05	0.90–1.23	0.525	44.5%	Fixed
	Acute/Late	Late	1795	22	0.81	0.62–1.07	0.139	3.6%	Fixed
		Acute	2950	31	1.29	0.90–1.85	0.168	53.0%	Random
	Cancer	Breast cancer	1538	11	0.85	0.55–1.31	0.462	53.9%	Random
		Head and neck cancer	1642	26	1.04	0.77–1.42	0.780	16.8%	Fixed
		Prostate cancer	688	5	0.88	0.51–1.52	0.656	0.0%	Fixed
		Non-small cell lung cancer	799	9	1.07	0.57–2.00	0.833	44.5%	Fixed
	Cutoff	Grade ≥2	3777	42	1.06	0.88–1.28	0.523	46.1%	Fixed
		Grade ≥3	820	8	0.98	0.59–1.62	0.943	46.5%	Random
	Side effects	Skin toxicity	1558	17	0.84	0.63–1.13	0.259	46.9%	Fixed
		Soft tissue injury	803	8	0.75	0.51–1.11	0.151	47.4%	Fixed
		Mucositis	579	9	1.30	0.78–2.16	0.310	0.0%	Fixed
	Treatment	RT	2164	18	1.03	0.63–1.68	0.905	66.9%	Random
		**RCT**	**746**	**14**	**1.86**	**1.25**–**2.77**	**0.002**	**0.0%**	**Fixed**
		RT+RCT	1383	16	0.87	0.60–1.26	0.463	22.1%	Fixed
GA vs GG	**Overall**		7732	**57**	**1.17**	**1.05**–**1.30**	**0.004**	**40.4%**	**Fixed**
	Acute/Late	Late	2660	22	1.00	0.83–1.20	0.985	22.4%	Fixed
		**Acute**	4248	**31**	**1.29**	**1.12**–**1.48**	**0.001**	**47.3%**	**Fixed**
	Cancer	Breast cancer	2369	11	1.07	0.89–1.28	0.470	13.9%	Fixed
		**Head and neck cancer**	2194	**26**	**1.25**	**1.03**–**1.52**	**0.025**	**45.2%**	**Fixed**
		Prostate cancer	1161	5	1.03	0.73–1.44	0.885	0.0%	Fixed
		Non-small cell lung cancer	1066	9	1.09	0.84–1.43	0.526	10.7%	Fixed
GA vs GG	Cutoff	**Grade ≥2**	5472	**42**	**1.19**	**1.05**–**1.35**	**0.006**	**29.2%**	**Fixed**
		Grade ≥3	1208	8	1.47	0.86–2.50	0.162	63.1%	Random
	Side effects	Skin toxicity	2249	17	1.10	0.91–1.33	0.347	19.0%	Fixed
		Soft tissue injury	1171	8	1.07	0.66–1.72	0.796	57.9%	Random
		**Mucositis**	**781**	**9**	**1.70**	**1.13**–**2.55**	**0.011**	**31.6%**	**Fixed**
	Treatment	**RT**	**3198**	**18**	**1.27**	**1.08**–**1.49**	**0.003**	**50.0%**	**Fixed**
		RCT	1036	14	1.23	0.94–1.61	0.134	47.6%	Fixed
		RT+RCT	1853	16	1.11	0.88–1.39	0.390	43.2%	Fixed
GA+AA vs GG	**Overall**		**10200**	**67**	**1.18**	**1.07**–**1.30**	**0.001**	**48.8%**	**Fixed**
	Acute/Late	Late	3278	23	0.93	0.79–1.10	0.401	23.7%	Fixed
		**Acute**	**5953**	**40**	**1.42**	**1.17**–**1.73**	**0.000**	**50.6%**	**Random**
	Cancer	Breast cancer	3469	18	1.03	0.88–1.20	0.738	9.6%	Fixed
		Head and neck cancer	2526	26	1.19	0.99–1.43	0.068	49.9%	Fixed
		Prostate cancer	1310	5	1.00	0.72–1.38	0.985	0.0%	Fixed
		Non-small cell lung cancer	1298	9	1.13	0.89–1.45	0.319	25.8%	Fixed
	Cutoff	**Grade ≥2**	**6566**	**45**	**1.15**	**1.03**–**1.29**	**0.013**	**47.3%**	**Fixed**
		**Grade ≥3**	**2311**	**15**	**1.57**	**1.07**–**2.30**	**0.020**	**52.2%**	**Random**
	Side effects	Skin toxicity	2719	19	1.06	0.89–1.27	0.498	26.7%	Fixed
		Soft tissue injury	1579	9	0.92	0.60–1.41	0.706	61.4%	Random
		**Mucositis**	**902**	**9**	**1.52**	**1.13**–**2.06**	**0.006**	**27.2%**	**Fixed**
	Treatment	RT	3913	20	1.23	0.95–1.61	0.119	62.5%	Random
		**RCT**	**2163**	**21**	**1.55**	**1.25**–**1.92**	**0.000**	**39.7%**	**Fixed**
		RT+RCT	2214	17	1.07	0.86–1.32	0.548	45.3%	Fixed

RCT, radio-chemotherapy; RT, radiotherapy. The bold values mean that they are statistically significant.

#### Meta-Analysis of Adjusted Data

Among all the included studies focusing on association between XRCC1 rs25487 and treatment response/side effects, adjusted data of side effects were available in seven studies, adjusted data of treatment response were available in two studies. Adjusted factors included age, tumor size, body mass index, adjuvant treatment, dose of radiotherapy, smoking status and so on. When we used adjusted data to analyze the association between rs25487 and side effects/treatment response, no significant association were found for both side effects analysis and treatment response analysis, which were consistent with our results when we used the crude data in corresponding studies. **Supplementary Files 11** and **12** show characteristics of studies with adjusted data and the forest plots of adjusted data and crude data of overall analyses for treatment response/side effects, respectively.

#### Subgroup Analysis

In the treatment response analysis, the GA genotype (OR = 0.50, 95% CI: 0.30–0.83, P = 0.008) was associated with a reduced risk of minor treatment response in esophageal cancer. The AA genotype (OR = 0.52, 95% CI: 0.31–0.86, P = 0.012) was associated with a reduced risk of minor treatment response when using grade ≥3 as the cutoff value (**Table 3**). We found that this SNP does not predict treatment response in rectal cancer, which is consistent with previous studies (16).

In the side effects analysis, an increased risk was found to be associated with the GA genotype (OR = 1.25, 95% CI: 1.03–1.52, P = 0.025) in head and neck cancer. The results further validated the results for the GA genotype (OR = 1.70, 95% CI: 1.13–2.55, P = 0.011) and GA+AA genotype (OR = 1.52, 95% CI: 1.13–2.06, P = 0.006), in terms of mucositis. Genotypes with variant alleles were associated with increased risks of side effects in patients receiving radiotherapy (GA: OR = 1.27, 95% CI: 1.08–1.49, P = 0.003) or radio-chemotherapy (AA: OR 1.86, 95% CI: 1.25–2.77, P = 0.002; GA+AA: OR = 1.55, 95% CI: 1.25–1.92, P = 0.000). GA (OR = 1.29, 95% CI: 1.12–1.48, P = 0.001) and the GA+AA genotype (OR = 1.42, 95% CI: 1.17–1.73, P = 0.000) was found to be associated with an increased risk of acute side effects. Heterozygous and mutant homozygotes were also associated with an increased risk of side effects when using Grade ≥2 or ≥3 as the cutoff value (**Table 4**).

#### Sensitivity Analysis and Publication Bias

In the sensitivity analysis, we removed the studies one by one to clarify their influence on the results. The confidence intervals for all the studies indicated that the results are stable. **Table 5** shows the P value for Begg’s rank correlation test and Egger’s linear response test. In addition, we found publication bias in the heterozygote and dominant models of side effects analysis (**Table 5**). **Supplementary Files 6** and **7** show the sensitivity analysis of treatment response and side effects, respectively. **Supplementary Files 9** and **10** show the publication bias in treatment response and side effects, respectively.

**Table 5:** P value in publication bias.

Group	Test	GA vs GG	AA vs GG	GA+AA vs GG
Treatment response	Begg	0.263	0.529	0.535
	Egger	0.116	0.542	0.161
Side effects	Begg	**0.042**	0.660	**0.006**
	Egger	0.095	0.673	**0.024**

P<0.05 means publication bias is statistically significant. The bold values mean that they are statistically significant.

## Discussion

Encoded by XRCC1 (X-ray repair cross complementing 1) gene, XRCC1 plays a crucial role in the oxidative DNA damage repair through the base excision repair (BER) pathway and single-stranded break repair (SSBR) processes, after exposure to ionizing irradiation or alkylating agents (80). XRCC1 functions as a scaffold protein to assemble a complex with polymerase beta (polβ), DNA ligase III (lig III), and poly (ADP-ribose) polymerase (PARP) (81). Changes in the expression of XRCC1 can affect the ability of cells to repair DNA damage, which may influence cell radiosensitivity. Previous studies have revealed that high XRCC1 expression levels are associated with resistance to radiotherapy in patients with lung, head, and neck cancer (82). However, conclusions about the relationship between XRCC1 expression and treatment efficacy are not consistent. On the other hand, XRCC1 SNPs are also associated with radiotherapy-related outcomes (65), and one of the most common polymorphisms of XRCC1 (rs25487; Arg399Gln) can impair the repair process through a missense mutation in exon 10 (codon 399) which results in dysfunction of the binding domain of PARP or polynucleotide kinase (PNK) (83). Studies have demonstrated that the XRCC1 rs25487 polymorphism is associated with an increased risks for several malignancy types. However, its association with RT-based treatment response and RT-induced normal tissue damage has not been to consistently confirmed.

To the best of our knowledge, this is the first comprehensive meta-analysis with the largest sample size investigating both the relationship between XRCC1 expression and radiotherapy-related treatment response/overall survival and the association between the XRCC1 r25487 polymorphism and radiotherapy-related treatment response/radiation-induced side effects. The results demonstrated that high XRCC1 expression is correlated with poor treatment response. For OS, the pooled HRs showed a strong correlation between high XRCC1 expression and poor OS, indicating that high XRCC1 expression could be considered a risk factor in cancer patients receiving radiotherapy. To further validate the radiotherapy-related prognostic value of XRCC1, we performed a TCGA data review. The pooled HRs indicated that high XRCC1 expression tended to be correlated with poor OS, although the result was not statistically significant. However, interestingly, when we performed a TCGA analysis in different tumor types without considering therapeutic modalities, we found that high XRCC1 expression was significantly related to better OS, indicating that high XRCC1 expression is a protective factor in the undifferentiated treatment cancer population. Studies have demonstrated that XRCC1 facilitates efficient DNA damage processing, which is pertinent in patients undergoing radiochemotherapy. This is mitigated to a certain extent by non−specific DNA repair systems; therefore, high XRCC1 expression levels may increase the DNA repair capacity of tumor cells, leading to an increased tolerance to DNA damage induced by radiochemotherapy (7). On the other hand, studies have also suggested that XRCC1 deficiency can sensitize cells to irradiation, and this enhanced sensitivity could be attributed to increased DNA damage and increased cell cycle arrest, which might be related to an increase in DNA-PKcs and gadd153 mRNA expression (84). All these evidences may explain why high XRCC1 expression is associated with poor radiotherapy-related treatment response and OS, which was identified in our meta-analysis results. Regarding TCGA results without considering therapeutic modalities, some studies concluded that high XRCC1 expression was significantly associated with early clinical stages and nodal status (85, 86). These results suggest that XRCC1 may play its normal role and act to protect individuals. However, one of our included studies (8) did not indicate an association between these parameters. One of the reasons may be that we did not consider the cancer type, and the impact of XRCC1 may differ from cancer to cancer, which may lead to conflicting results, further investigations are needed.

No high-throughput studies (such as GWAS) were found that reported a correlation between XRCC1 rs25487 and radiotherapy-related prognosis. However, given that many single variable studies with different sample sizes that drew different conclusions about the relationship between XRCC1 r25487 and radiotherapy-related prognosis, this meta-analysis is of special importance to obtain a more robust conclusion with higher statistical power. Overall, we found an increased risk of side effects (RT-induced normal tissue damage) associated with the GA genotype and GA + AA genotype, compared to the GG genotype. Furthermore, the GA + AA genotype was found to be associated with acute and severe side effects, especially in terms of mucositis in head and neck cancer (HNC). PARP and PNK are two important enzymes in the BER pathway. The variant alleles of XRCC1 rs25487 might significantly change the affinity of the binding domain of PARR or PNK (83), which could result in a reduced efficiency of DNA damage repair processes in the acute damage phase. However, in the late damage phase, which usually takes a period of months to years for late damage to manifest itself after the end of radiotherapy. It is a far more complicated event than the acute damage phase and has still not been fully elucidated. Radiobiological studies have demonstrated that irradiation initiates a network of pro- and anti-inflammatory cytokine cascades, which are crucial for tissue regeneration and healing. However, the balance of network changes with time and space during the late damage phase. With depletion of target cells and a lack of stem cells, it ends up a failure to regenerate functional tissue, which is replaced by fibrogenesis. Our study found that the XRCC1 SNP is associated with radiation-induced acute side effects instead of late side effects in HNCs. Further research is needed to determine whether XRCC1 plays a role in the late damage phase.

The era of advanced multimodality radiotherapy requires the development of approaches for tailoring treatments to the individual. Irradiation-related genomic biomarkers can contribute by identifying sufficient genetic variants associated with a patient’s risks of radiotherapy-related toxicity and cancer prognosis. Increasing clinical trials (ClinicalTrials.gov identifier: NCT00122239; NCT02573636; NCT00099112; NCT02112162; NCT03296124) are focusing on the relationship between genes and radiotherapy-related side effects, most of the results have not been released. One of our included studies is registered on the ClinicalTrials database (identifier: NCT01316328), which indicates no relationship between XRCC1 rs25487 and radiotherapy-induced fibrosis or fat necrosis in breast cancer patients. Our meta-analysis shows no relationship between XRCC1 rs25487 and radiotherapy-induced soft tissue injury, which confirmed the clinical trial results from a broader perspective with a larger sample size. The evidence above shows that it will be valuable that referencing our meta-analysis results when using the XRCC1 gene and XRCC1 rs25487 polymorphisms as biomarkers for patients receiving radiotherapy-related treatments in clinical practice.

According to statistics, nearly 90% of patients suffer from different grades of mucositis during radiotherapy (87), which has a great detrimental impact on the radiation effect and local control rate, as well as on long-term survival. However, in the subgroup analysis, we found that the XRCC1 rs25487 GA genotype was associated with a reduced risk of minor treatment response in esophageal cancer, which indicates that it is a prognostic factor of better treatment response for people with esophageal cancer under radiotherapy-related treatment. For more than a decade, studies (88) have shown that patients with variant alleles (GA + AA) of XRCC1 rs25487 have a lower pathological complete response (pCR) rate after neoadjuvant chemoradiotherapy. The meta-analysis in the present study further confirmed this result. According to our analysis, patients with the XRCC1 GA genotype and XRCC1 AA genotype are more likely to suffer from grade II or higher acute side effects, especially mucositis. The XRCC1 SNP might be a potential biomarker to tailor individual radiotherapy. In treatment response analysis, we found that the XRCC1 rs25487 GA genotype was associated with reduced risk of minor treatment response in esophageal cancer. In addition, there was no difference in treatment-related toxicity between the RT and RCT groups.

However, some methodological issues should be taken into consideration. First, the sample size among the included studies ranged from 50 to 575, which is relatively small. Thirty-three studies had fewer than 100 patients and only five studies had more than 400 patients. Andreassen et al. reported that studies with small sample sizes were underpowered to detect SNPs with a modest impact on complication risk (89). Therefore, both the conclusions about XRCC1 expression and cancer prognosis and the association between rs25487 and treatment response/side effects should be carefully assessed. Second, most included studies about XRCC1 expression and cancer prognosis are from the Chinese population, and most included XRCC1 SNP analysis studies did not provide information on ethnicity; thus, we could not conduct further research on the association in different populations. Therefore, further studies should specify the ethnicity of the patients involved. Third, in some articles, the HR value was not provided, and we had to extract the HR value from the K-M curve, a process that may introduce errors. Furthermore, the therapeutic regimen, evaluation criteria, and cutoff values adopted in these studies were not uniform, especially the cutoff for XRCC1 expression. Only two studies classified the cutoff according to medians, and we extracted survival data in TCGA by selecting the median as the cutoff value, which may lead to our conclusions being less persuasive. To solve this problem, a unified XRCC1 cutoff value and a more detailed subgroup analysis (such as age, cancer, ethnicity etc.) are necessary. Finally, although our meta-analysis indicates a strong association between XRCC1 expression and rs25487 and radiotherapy-related side effects/treatment response, it does not necessarily mean that XRCC1 expression and rs25487 are the only factors that influenced these outcomes. The genotype-phenotype relationship shows that a gene alone can neither cause an observable phenotypic trait, nor can it be necessary and sufficient to the emergence of observable characteristics. Genes need a cellular environment, the combined action of multiple other genes, as well as certain physico-chemical conditions to have an observable effect on organisms (90). It is important to remember that phenotypes are equally, or even sometimes more greatly influenced by environmental effects than genetic effects. So, a phenotype can be directly related to a genotype, but not necessarily. Our results should be carefully interpreted, other multiple genes and SNPs, environmental effects (like age, radiation dose, radiation quality, adjuvant treatment and so on) can all be the factors that influenced side effects and treatment response. Therefore, further studies should pay greater attention to detailed treatment parameters. These efforts might contribute to bringing radiation therapy from physical precision to biological precision.

## Conclusion

Our meta-analysis suggested that high XRCC1 expression is associated with increased risk of minor treatment response and poor OS. XRCC1 rs25487 is associated with reduced risk of minor radiotherapy-related/radiation-induced treatment response in esophageal cancer and increased risk of mucositis in head and neck cancer. Considering the insufficient reporting of treatment parameters and the various sample sizes for different cancer types, we suggest that genetic association studies related to radiation-based treatment should include more cancer types and patients with sufficient statistical power and more detailed clinical parameters.

## Data Availability Statement

The datasets supporting the conclusions of this article are included within the article and its additional files.

## Author Contributions

ZL designed the study. LG, ML, and RS reviewed articles and collected data. LG performed statistical analysis. LG, ML, and RS wrote the manuscript. LQ and CC revised the manuscript. All authors contributed to the article and approved the submitted version.

## Funding

This work was supported by the National Natural Science Foundation of China (No.81802997), the HuBei Provincial Department of Science and Technology Innovation Group Programme (No.2019CFA034), The Free Exploration Foundation of the Hubei University of Medicine (No.FDFR201802).

## Conflict of Interest

The authors declare that the research was conducted in the absence of any commercial or financial relationships that could be construed as a potential conflict of interest.
