# Supplementary material for: Significant Association Between XRCC1 Expression and Its rs25487 Polymorphism and Radiotherapy-Related Cancer Prognosis
Source: Front Oncol. 2021 May 19;11:654784. doi: 10.3389/fonc.2021.654784 (PMC8170393; doi:10.3389/fonc.2021.654784)
Supplement: Supplementary file 1 [file DataSheet_1.pdf]

Supplementary Table 1

Supplementary Table 1a. Characteristics of included studies associated with XRCC1 expression and treatment response

Study	Country	Cancer type	Treatment	Evaluation criteria	Cut-off for evaluation (responder)	cut-off for XRCC1 expression (high/positive)	Sample size	XRCC1 high/positive		XRCC1 low/negative		Association
								Responder	poor responder	Responder	poor responder	
Liu (2010)	China	Esophageal squamous cell carcinoma	RT	Unknown	CR+PR	10%	59	25	6	26	2	No
Zhao (2010)	China	Non-small cell lung cancer	RT	WHO	CR+PR	50%	62	6	9	31	16	No
Sakano (2013)	Japan	Bladder cancer	RCT	Unknown	CR	H-score ≥1.0	142	32	56	20	34	No
Zheng (2015)	China	Esophageal squamous cell carcinoma	RT	RECIST	CR+PR	> 4 scores	76	26	14	28	8	No
Geng (2016)	China	Gastric cancer	RT	RECIST	CR+PR	NM	46	13	17	14	2	Yes
Huang (2017)	China	Rectal cancer	RCT+surgery	TRG	CR	50%	86	31	20	26	9	No
Zhang (2017)	China	Esophageal squamous cell carcinoma	RT	RECIST	CR	2-6 scores	76	22	18	26	10	No

RT: Radiotherapy; **RCT**: Radio-chemotherapy; **CR**: Complete Response; **PR**: Partial Response; **WHO**: world health organization; **TRG**: Tumor Regression Grade; **RECIST**: Response Evaluation Criteria In Solid Tumors.

Supplementary Table 1b. Characteristics of included studies associated with XRCC1 expression and overall survival

Study	Country	Cancer	Outcome	follow-up duration (month)	Treatment	Cut-off value for XRCC1 expression(high/positive)	Sample size (high/low expression)	HR for OS(95%CI)	Association
Ang (2011)	America	Head and neck squamous cancer	OS/PFS	39-87	RCT + surgery	8 score	68(44/24)	6.02(2.36-15.37)	Yes
Ge (2014)	China	Esophageal squamous cell carcinoma	OS	4-82	RCT + surgery	2-6 scores	44(24/20)	1.09(0.43-2.77)	No
Zheng (2015)	China	Esophageal squamous cell carcinoma	OS	4-55	RT	> 4 scores	76(40/36)	1.48(0.84-2.60)	No
Geng (2016)	China	Gastric cancer	OS	24	RT	NM	46(30/16)	1.45(0.36-6.96)	No
Huang (2017)	China	Rectal cancer	OS/DFS	7-54	RCT	50%	86(51/35)	1.80(0.48-6.82)	No
Zhang (2017)	China	Esophageal squamous cell carcinoma	OS	7-78	RT	2-6 scores	76(40/36)	1.08(0.59-1.99)	No

OS: overall survival; **PFS**: progression-free survival; **RT**: Radiotherapy; **RCT**: Radio-chemotherapy **DFS**: disease-free survival; **NM**: not mentioned

Supplementary Table 1c. Characteristics of included studies associated with XRCC1 rs25487 and treatment response

Study	Cancer	Treatment	Evaluation	Cut-off	Case			Control			Association
					GG	GA	AA	GG	GA	AA	
Sakano (2006)	Bladder cancer	RCT	Unknown	No change	12	4		35	21		No
Qing-hua (2007)	Non-small cell lung cancer	RCT	WHO	Stable disease + Progressive disease	41	23	4	35	15	2	No
Warnecke-Eberz (2009)	Esophageal cancer	RCT	Histomorphologic Grading of Tumor Regression	Grade ≥3	18	1	9	10	3	9	No
Xu-sheng (2010)	Esophageal squamous cell carcinoma	RT	Unknown	No remission	4	3	8	43	24	12	Yes
Grimminger (2010)	Rectal cancer	RCT	Histomorphologic Grading of Tumor Regression	Grade ≥3	27	11	17	8	3	15	No
Lamas (2010)	Rectal cancer	RCT	TRG	Grade ≥3	3	29	17	4	13	27	Yes
Balboa (2010)	Rectal cancer	RCT	TRG	Grade ≥3	14	19	1	16	12	3	No
Cecchin (2011)	Rectal cancer	RCT	TRG	Grade ≥3	44	56	14	45	52	24	No
Yoon (2011)	Esophageal adenocarcinoma	RCT	Unknown	No complete pathologic response	21	29		8	2		No
Paez (2011)	Rectal cancer	RCT	Unknown	Stable disease + Progressive disease	25	35	8	24	28	6	No
Yan-yan (2012)	Non-small cell lung cancer	RCT	WHO	Stable disease + Progressive disease	11	15		13	13		No
He (2014)	Esophageal squamous cell carcinoma	RT	RECIST	Stable disease + Progressive disease	13	5	7	19	38	68	Yes
Xiao-mei (2014)	Cervix squamous cell carcinoma	RT	Unknown	Partial remission	8	14	4	18	25	4	No
Wei (2014)	Non-small cell lung cancer	RT	RECIST	Stable disease + Progressive disease	13	8	4	18	10	7	No
Xian (2015)	Esophageal squamous cell carcinoma	RCT	RECIST	Stable disease + Progressive disease	17	13	5	19	17	2	No
Meng-xin (2015)	Nasopharyngeal carcinoma	RCT	RECIST	Partial remission	7	6	2	53	41	5	No
Xiao-Ming (2016)	Nasopharyngeal cancer	RCT	WHO	Stable disease + Progressive disease	2	0	0	31	21	6	No
Xue (2017)	Esophageal squamous cell carcinoma	RCT	RECIST	No complete remission	8	10	2	5	12	13	Yes
Xiujin (2017)	Esophageal squamous cell carcinoma	RT+RCT	RECIST	Stable disease + Progressive disease	11	5	0	39	38	4	No
Zhang (2017)	Nasopharyngeal carcinoma	RCT	RECIST	Partial remission	6	6		40	48		No
Wang (2017)	Nasopharyngeal carcinoma	RT+RCT	RECIST	No complete remission	7	3	0	84	28	6	No
Zhang (2018)	Rectal cancer	RCT	TRG	Grade ≥3	17	13		15	10		No
Nicosia (2018)	Rectal cancer	RCT	TRG	Grade ≥4	12	25	4	18	14	7	No
Yang (2020)	Non-small cell lung cancer	RT	RECIST	Stable disease + Progressive disease	111	98	43	130	81	23	Yes

RT: Radiotherapy; **RCT**: Radio-chemotherapy; **WHO**: World Health Organization; **TRG**: Tumor Regression Grade; **RECIST**: Response Evaluation Criteria In Solid Tumors.

Supplementary Table 1d. Characteristics of included studies associated with XRCC1 rs25487 and side effects

Study	Cancer	Treatment	Side effects	Acute/Late	Evaluation criteria	Cut-off	Case			Control			Association
							GG	GA	AA	GG	GA	AA	
Moullan (2003)	Breast cancer	RT	Adverse reaction	Late	EORTC Late Radiation Morbidity Scoring Scheme	Grade ≥1	85	76	23	24	37	9	No
Chang-Claude (2005)	Breast cancer	RT	Radiation dermatitis	Acute	CTCAE 2.0	Grade ≥2	31	36	10	150	168	51	No
Giotopoulos (2007)	Breast cancer	RCT	Telangiectasia	Late	LENT-SOMA Score	Score ≥2	2	9	4	33	25	9	Yes
Suga (2007)	Breast cancer	RT	Adverse skin reaction	Acute	CTC 2.0	Grade ≥2	168	107	5	59	36	14	Yes
Alsbeih (2008)	Nasopharyngeal carcinoma	RT+RCT	Fibrosis	Late	RTOG/EORTC Late Radiation Morbidity Scoring Scheme	Grade ≥2	21	4	0	17	5	3	Yes
Burri (2008)	Prostate adenocarcinoma	RT+(RT+HT)	Rectal bleeding	Late	RTOG/EORTC	Grade ≥2	3	2	1	63	59	7	No
Burri (2008)	Prostate adenocarcinoma	RT+(RT+HT)	Urinary Morbidity	Late	RTOG/EORTC	Grade ≥2	6	6	1	60	55	7	No
Burri (2008)	Prostate adenocarcinoma	RT+(RT+HT)	Erectile Dysfunction	Late	RTOG/EORTC	Grade ≥2	8	9	0	16	23	4	No
Falvo (2009)	Breast adenocarcinoma	RCT+(RT+HT)	Fibrosis or Fat necrosis	Late	CTCAE 3.0	Grade ≥2	50	63	14	112	120	44	No
Chang-Claude (2009)	Breast cancer	RT	Telangiectasia	Late	LENT-SOMA Score	Score ≥2	50	63	14	112	120	44	No
Popanda (2009)	Prostate cancer	RT	Side effects	Acute	CTC 2.0	Grade ≥3	17	33	4	136	169	46	No
Zschenker (2010)	Breast cancer	RT+RCT+(RT+HT) +(RCT+HT)	Fibrosis	Late	LENT-SOMA Score	Grade ≥2	4	8	5	25	17	10	No
Alsbeih (2010)	Nasopharyngeal carcinoma	RT+RCT	Fibrosis	Late	RTOG/EORTC	Grade ≥2	25	5	0	20	6	4	No
Mangoni (2010)	Breast cancer	RT+RCT	Skin toxicity	Acute	CTCAE	Grade ≥2	2	6		39	40		No
Mangoni (2010)	Breast cancer	RT	Skin toxicity	Acute	CTCAE	Grade ≥2	2	5		28	26		No
Liqing (2010)	Breast cancer	RT	Skin toxicity	Acute	CTCAE 3.0	Grade ≥2	39	24	6	58	34	10	No
Sakano (2010)	Bladder cancer	RCT	Anemia	Acute	CTCAE 3.0	Grade ≥3	6	3		53	33		No
Sakano (2010)	Bladder cancer	RCT	Leukocytopenia	Acute	CTCAE 3.0	Grade ≥3	8	7		51	29		No
Sakano (2010)	Bladder cancer	RCT	Neutropenia	Acute	CTCAE 3.0	Grade ≥3	11	8		48	28		No
Sakano (2010)	Bladder cancer	RCT	Thrombocytopenia	Acute	CTCAE 3.0	Grade ≥3	0	2		59	34		Unknown
Ishikawa (2010)	Cervical cancer	RT	Adverse reaction	Acute	CTC 2.0	Grade ≥2	31	22	5	83	55	12	No
Ming (2011)	Non-small cell lung cancer	RT+RCT+(RT+Other)	Radiation pneumonitis	Unknown	CTCAE 3.0	Grade ≥2	25	28	13	24	44	31	Yes

Pratesi (2011)	Head and neck squamous cell carcinoma	RCT	Dysphagia	Acute	CTCAE	Grade ≥2	4	5	3	39	33	17	No
Pratesi (2011)	Head and neck squamous cell carcinoma	RCT	Erythema	Acute	CTCAE	Grade ≥2	12	18	9	31	20	11	Yes
Pratesi (2011)	Head and neck squamous cell carcinoma	RT	Erythema	Acute	CTCAE	Grade ≥2	4	5	3	18	12	13	Yes
Pratesi (2011)	Head and neck squamous cell carcinoma	RCT	Mucositis	Acute	CTCAE	Grade ≥2	23	31	14	20	7	6	Yes
Pratesi (2011)	Head and neck squamous cell carcinoma	RT	Mucositis	Acute	CTCAE	Grade ≥2	7	11	4	15	6	2	Yes
Langsenlehner (2011)	Prostate cancer	RT+(RT+HT)	Bladder and/or rectal toxicity	Late	RTOG/EORTC	Grade ≥2	39	39	13	191	227	66	No
Terrazzino (2011)	Breast cancer	RT	Fibrosis	Late	LENT-SOMA Score	Grade ≥2	18	23		77	119		No
Yoon (2011)	Esophageal adenocarcinoma	RCT	Radiation-induced toxicity	Acute	CTCAE 2.0	Grade ≥3	5	7		24	24		No
Raabe (2012)	Breast cancer	RT+(RCT+HT)	Erythema	Acute	RTOG Acute Radiation Morbidity Scoring Criteria	Grade ≥2	19	20	7	17	13	7	No
Terrazzino (2012)	Breast cancer	RT+RCT+(RT+HT) +(RCT+HT)	Skin toxicity	Acute	RTOG	Grade ≥2	35	42	12	78	95	23	No
Haijun (2013)	Nasopharyngeal carcinoma	RT+RCT	Radiation dermatitis	Acute	CTCAE 3.0	Grade ≥3	10	14	0	53	28	9	Yes
Haijun (2013)	Nasopharyngeal carcinoma	RT+RCT	Mucositis	Acute	CTCAE 3.0	Grade ≥3	23	23	2	40	19	7	No
Duldulao (2013)	Rectal cancer	RCT	Adverse events	Acute	CTCAE 3.0	Grade ≥3	7	56		93	191		Yes
Tucker (2013)	Non-small cell lung cancer	RT+RCT	Radiation pneumonitis	Unknown	CTCAE 3.0	Grade ≥3	5	11	12	40	63	38	Yes
Zhu (2013)	Esophageal squamous cell carcinoma	RT+RCT	Radiation esophagitis	Acute	CTCAE 4.0	Grade ≥2	23	15	3	90	44	7	No
Zhu (2013)	Esophageal squamous cell carcinoma	RT+RCT	Radiation pneumonitis	Acute	CTCAE 4.0	Grade ≥2	18	7	2	95	52	8	No
Cheuk (2014)	Nasopharyngeal carcinoma	RT+RCT	Fibrosis	Late	RTOG	Grade ≥1	15	13	1	50	35	6	No
Venkatesh (2014)	Head and neck cancer	RT+RCT	Radiation dermatitis	Acute	RTOG	Grade >2	8	14	17	15	62	50	No
Venkatesh (2014)	Head and neck cancer	RT+RCT	Mucositis	Acute	RTOG	Grade >2	6	25	23	9	28	29	No
Alsbeih (2014)	Nasopharyngeal carcinoma	RT+RCT	Fibrosis	Late	RTOG/EORTC	Grade ≥3	38	7	3	58	39	10	Yes
Meng-xin (2015)	Nasopharyngeal squamous cell carcinoma	RT+RCT	Radiation dermatitis	Acute	CTCAE 4.0	Grade ≥3	1	6	2	59	41	5	Yes
Meng-xin (2015)	Nasopharyngeal squamous cell carcinoma	RT+RCT	Mucositis	Acute	CTCAE 4.0	Grade ≥3	8	11	2	52	36	5	No
Wei (2015)	Non-small cell lung cancer	RT	Skin toxicity	Late	RTOG/EORTC	Grade ≥2	15	7	5	16	11	6	No
Wei (2015)	Non-small cell lung cancer	RT	Radiation esophagitis	Late	RTOG/EORTC	Grade ≥2	20	11	8	11	7	3	No
Wei (2015)	Non-small cell lung cancer	RT	Radiation pneumonitis	Late	RTOG/EORTC	Grade ≥2	21	13	4	10	5	7	Yes

Wei (2015)	Non-small cell lung cancer	RT	Heart injury	Late	RTOG/EORTC	Grade ≥2	6	5	1	25	13	10	Yes
Wei (2015)	Non-small cell lung cancer	RT	Spinal cord injury	Late	RTOG/EORTC	Grade ≥2	2	2	0	29	16	11	Unknown
Mei-ling (2016)	Cervical cancer	RT	Cystitis	Acute	RTOG	Grade ≥2	12	23	12	66	33	6	Yes
Mei-ling (2016)	Cervical cancer	RT	Proctitis	Acute	RTOG	Grade ≥2	38	46	16	40	10	2	Yes
Mumbrekar (2016)	Breast cancer	RT+RCT	Skin toxicity	Acute	RTOG	Grade ≥2	20	18	5	32	35	9	No
Smith (2016)	Rectal cancer	RCT	Adverse events	Acute	CTCAE 3.0	Grade ≥3	2	12		62	89		No
Xiao-Ming (2016)	Nasopharyngeal squamous cell carcinoma	RCT	Radiation dermatitis	Late	RTOG	Grade ≥2	12	10	4	21	11	2	No
Xiao-Ming (2016)	Nasopharyngeal squamous cell carcinoma	RCT	Mucositis	Late	RTOG	Grade ≥2	2	4	0	31	17	6	No
Xiao-Ming (2016)	Nasopharyngeal squamous cell carcinoma	RCT	Salivary gland injury	Late	RTOG	Grade ≥2	2	4	0	31	17	6	No
Xiao-Ming (2016)	Nasopharyngeal squamous cell carcinoma	RCT	subcutaneous tissue injury	Late	RTOG	Grade ≥2	19	13	4	14	8	2	No
Xiao-Ming (2016)	Nasopharyngeal squamous cell carcinoma	RCT	Radiation dermatitis	Acute	RTOG	Grade ≥2	14	10	4	19	11	2	No
Xiao-Ming (2016)	Nasopharyngeal squamous cell carcinoma	RCT	Mucositis	Acute	RTOG	Grade ≥2	17	13	4	16	8	2	No
Xiao-Ming (2016)	Nasopharyngeal squamous cell carcinoma	RCT	Salivary gland injury	Acute	RTOG	Grade ≥2	21	14	6	12	7	0	No
Chen (2017)	Nasopharyngeal carcinoma	RCT	Dermatitis	Acute	RTOG	Grade ≥2	28	13	1	32	32	8	Yes
Chen (2017)	Nasopharyngeal carcinoma	RCT	Mucositis	Acute	RTOG	Grade ≥2	42	27	6	18	18	3	N0
Wang (2017)	Nasopharyngeal carcinoma	RT+RCT	Mucositis	Acute	RTOG/EORTC	Grade ≥3	61	20	2	68	19	4	N0
Wang (2017)	Nasopharyngeal carcinoma	RT+RCT	Dermatitis	Acute	RTOG/EORTC	Grade ≥2	33	12	1	96	27	5	N0
Du (2018)	Lung cancer	RT	Radiation pneumonitis	Unknown	CTCAE4.0	Grade ≥2	36	18	3	51	34	7	N0
Yang (2020)	Non-small cell lung cancer	RT	Adverse reaction	Unknown	RTOG/EORTC	Grade ≥2	74	69	18	188	114	23	Yes
Xie (2020)	Non-small cell lung caner	RCT	Lymphopenia	Acute	CTCAE5.0	ALC < 0.3×10*9 /L	32	37	33	24	43	9	Yes

RT: Radiotherapy; **RCT**: Radio-chemotherapy; **HT**: Hormonal therapy; **EORTC**: European Organization for Research and Treatment; **RTOG**: Radiation Therapy Oncology Group; **CTCAE**: Common Terminology Criteria for Adverse Events ; **CTC**: Common Terminology Criteria; **LENT-SOMA**: Late Effects Normal Tissue/Subjective Objective Management Analytic; **ALC**: Absolute Lymphocyte Count

Supplementary File 1

Overall and subgroup analysis for association between XRCC1 expression and treatment response.

The subgroup includes types of cancer and treatment

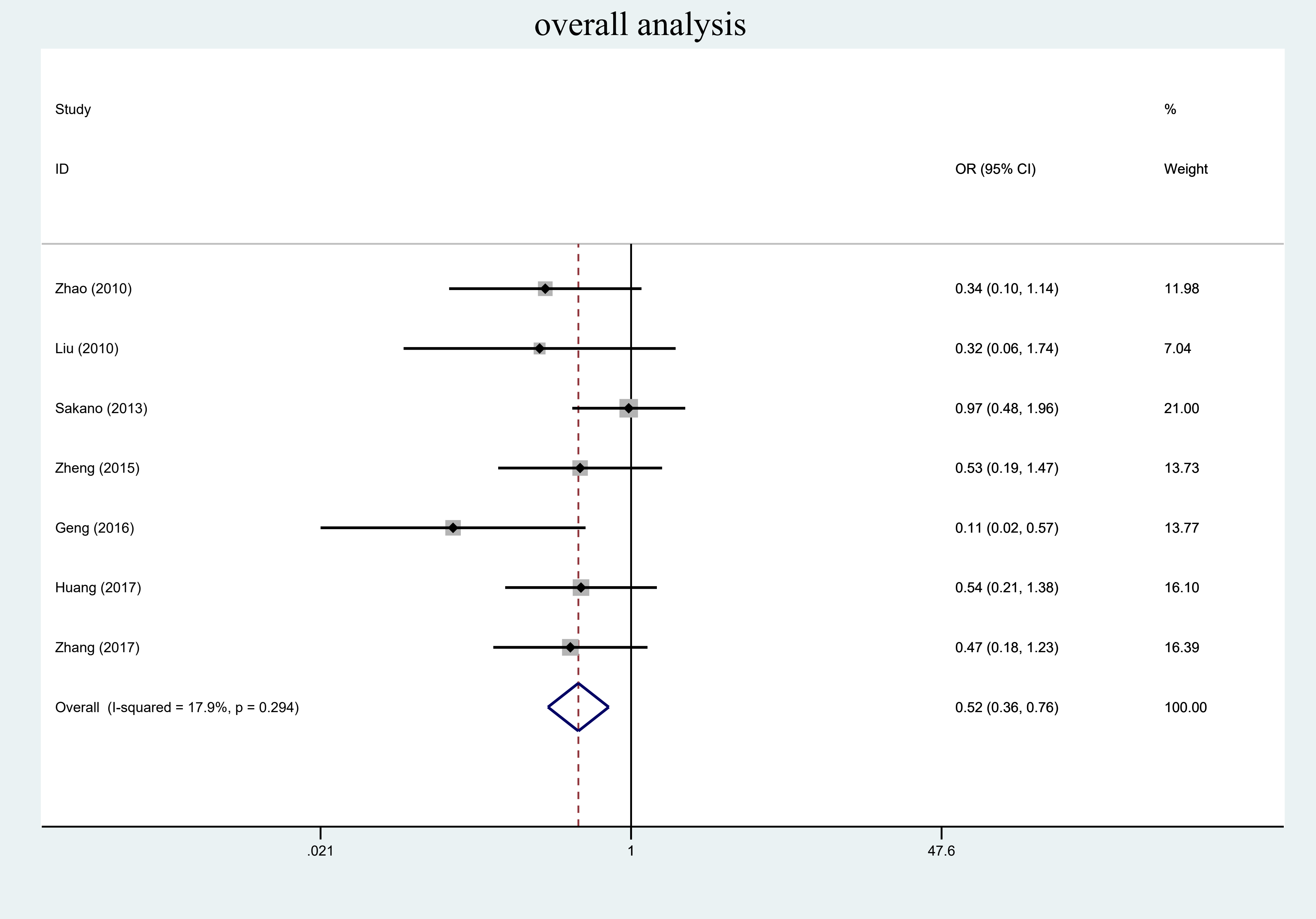

overall analysis
Study
ID
OR (95% CI)
%
Weight
Zhao (2010)
0.34 (0.10, 1.14)
11.98
Liu (2010)
0.32 (0.06, 1.74)
7.04
Sakano (2013)
0.97 (0.48, 1.96)
21.00
Zheng (2015)
0.53 (0.19, 1.47)
13.73
Geng (2016)
0.11 (0.02, 0.57)
13.77
Huang (2017)
0.54 (0.21, 1.38)
16.10
Zhang (2017)
0.47 (0.18, 1.23)
16.39
Overall (I-squared = 17.9%, p = 0.294)
0.52 (0.36, 0.76)
100.00
.021
1
47.6

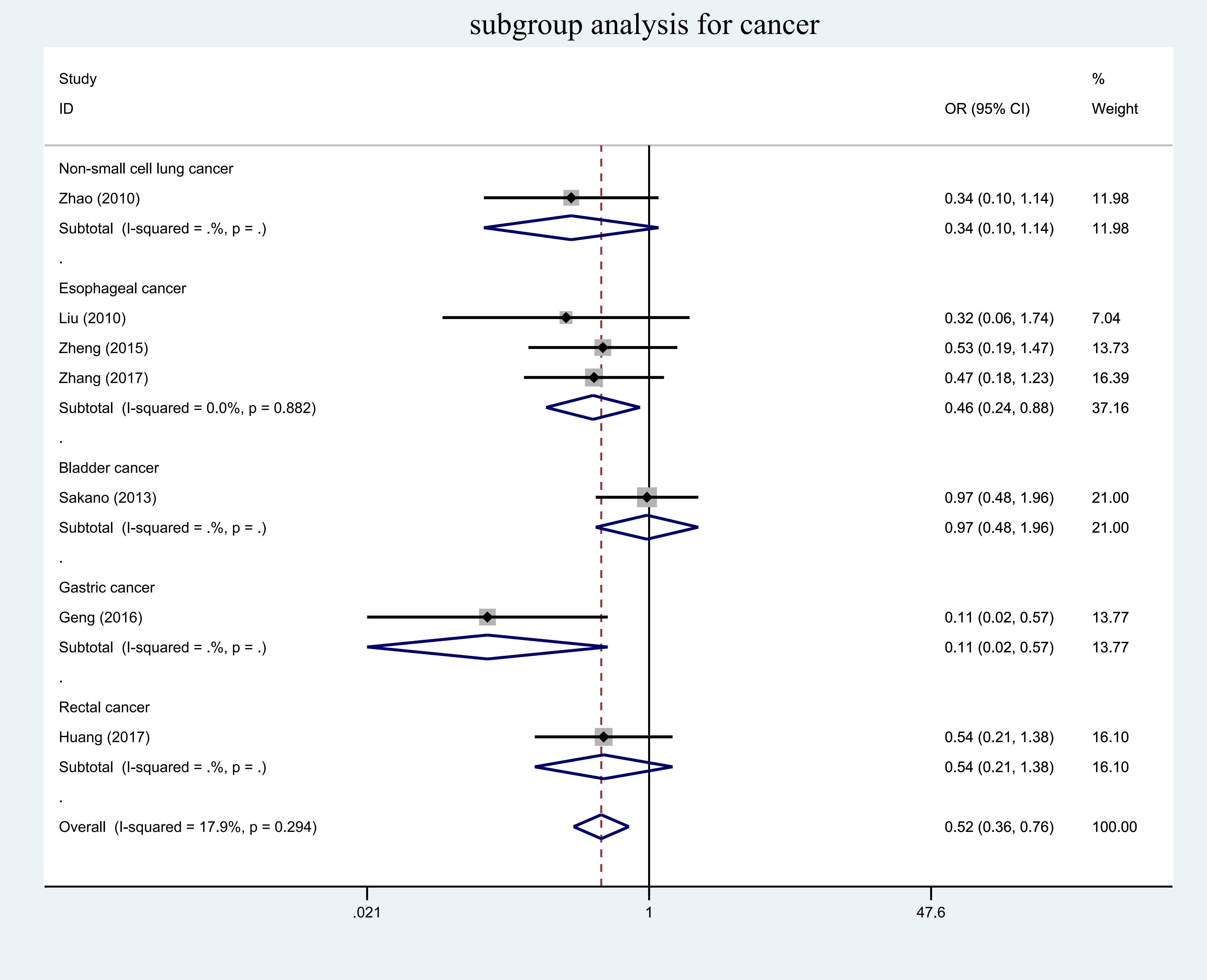
subgroup analysis for cancer
Study
ID
OR (95% CI)
%
Weight
Non-small cell lung cancer
Zhao (2010)
0.34 (0.10, 1.14)
11.98
Subtotal (I-squared = .%, p = .)
0.34 (0.10, 1.14)
11.98
.
Esophageal cancer
Liu (2010)
0.32 (0.06, 1.74)
7.04
Zheng (2015)
0.53 (0.19, 1.47)
13.73
Zhang (2017)
0.47 (0.18, 1.23)
16.39
Subtotal (I-squared = 0.0%, p = 0.882)
0.46 (0.24, 0.88)
37.16
.
Bladder cancer
Sakano (2013)
0.97 (0.48, 1.96)
21.00
Subtotal (I-squared = .%, p = .)
0.97 (0.48, 1.96)
21.00
.
Gastric cancer
Geng (2016)
0.11 (0.02, 0.57)
13.77
Subtotal (I-squared = .%, p = .)
0.11 (0.02, 0.57)
13.77
.
Rectal cancer
Huang (2017)
0.54 (0.21, 1.38)
16.10
Subtotal (I-squared = .%, p = .)
0.54 (0.21, 1.38)
16.10
.
Overall (I-squared = 17.9%, p = 0.294)
0.52 (0.36, 0.76)
100.00
.021
1
47.6

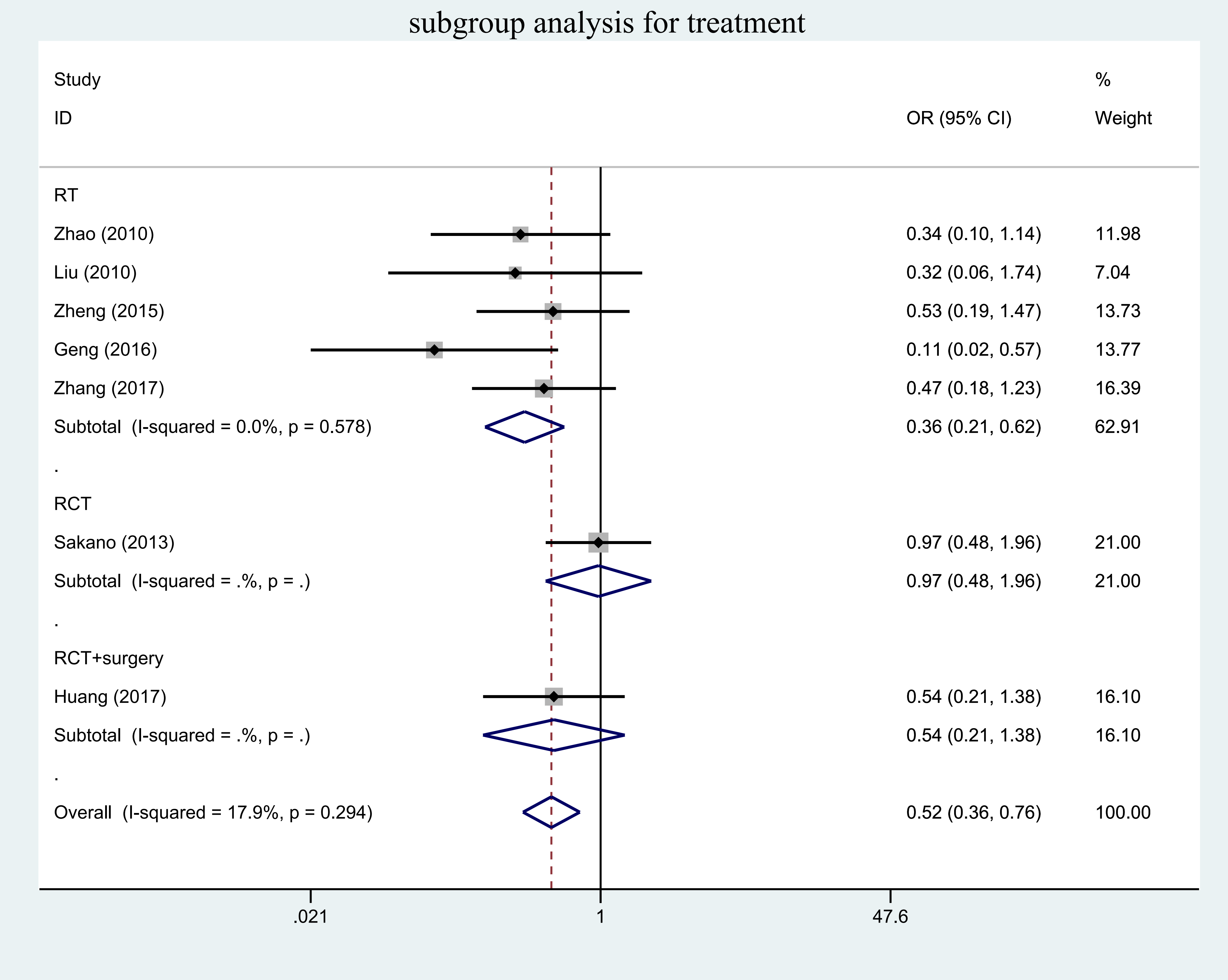
subgroup analysis for treatment
Study
ID
OR (95% CI)
%
Weight
RT
Zhao (2010) 0.34 (0.10, 1.14) 11.98
Liu (2010) 0.32 (0.06, 1.74) 7.04
Zheng (2015) 0.53 (0.19, 1.47) 13.73
Geng (2016) 0.11 (0.02, 0.57) 13.77
Zhang (2017) 0.47 (0.18, 1.23) 16.39
Subtotal (I-squared = 0.0%, p = 0.578) 0.36 (0.21, 0.62) 62.91
.
RCT
Sakano (2013) 0.97 (0.48, 1.96) 21.00
Subtotal (I-squared = .%, p = .) 0.97 (0.48, 1.96) 21.00
.
RCT+surgery
Huang (2017) 0.54 (0.21, 1.38) 16.10
Subtotal (I-squared = .%, p = .) 0.54 (0.21, 1.38) 16.10
.
Overall (I-squared = 17.9%, p = 0.294) 0.52 (0.36, 0.76) 100.00
.021
1
47.6

Supplementary File 2

Overall and subgroup analysis for association between XRCC1 expression and overall survival. The subgroup includes types of cancer and treatment.

TCGA data analysis for association between XRCC1 expression and overall survival.

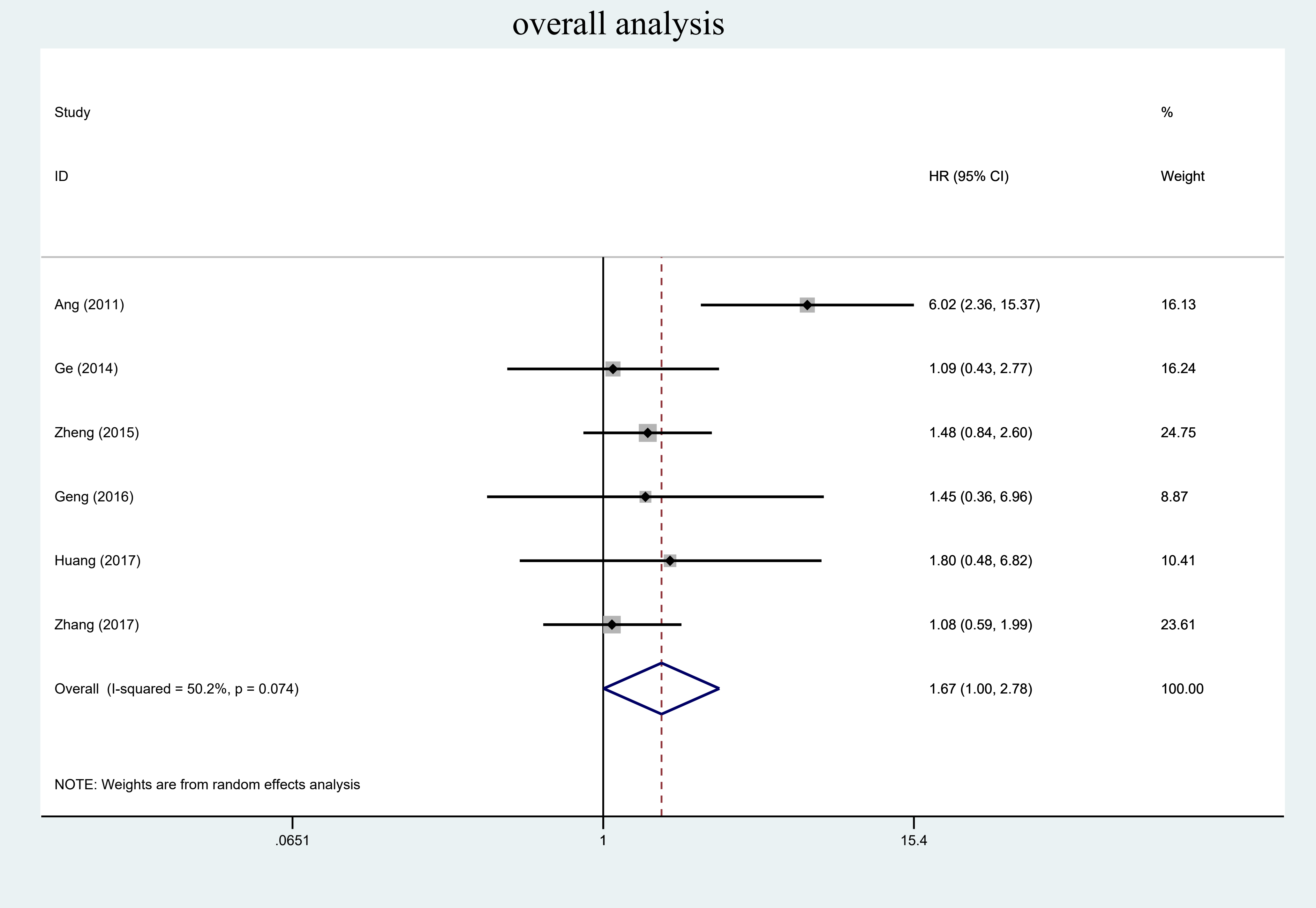
overall analysis
Study
ID
HR (95% CI)
%
Weight
Ang (2011)
6.02 (2.36, 15.37)
16.13
Ge (2014)
1.09 (0.43, 2.77)
16.24
Zheng (2015)
1.48 (0.84, 2.60)
24.75
Geng (2016)
1.45 (0.36, 6.96)
8.87
Huang (2017)
1.80 (0.48, 6.82)
10.41
Zhang (2017)
1.08 (0.59, 1.99)
23.61
Overall (I-squared = 50.2%, p = 0.074)
1.67 (1.00, 2.78)
100.00
NOTE: Weights are from random effects analysis
.0651
1
15.4

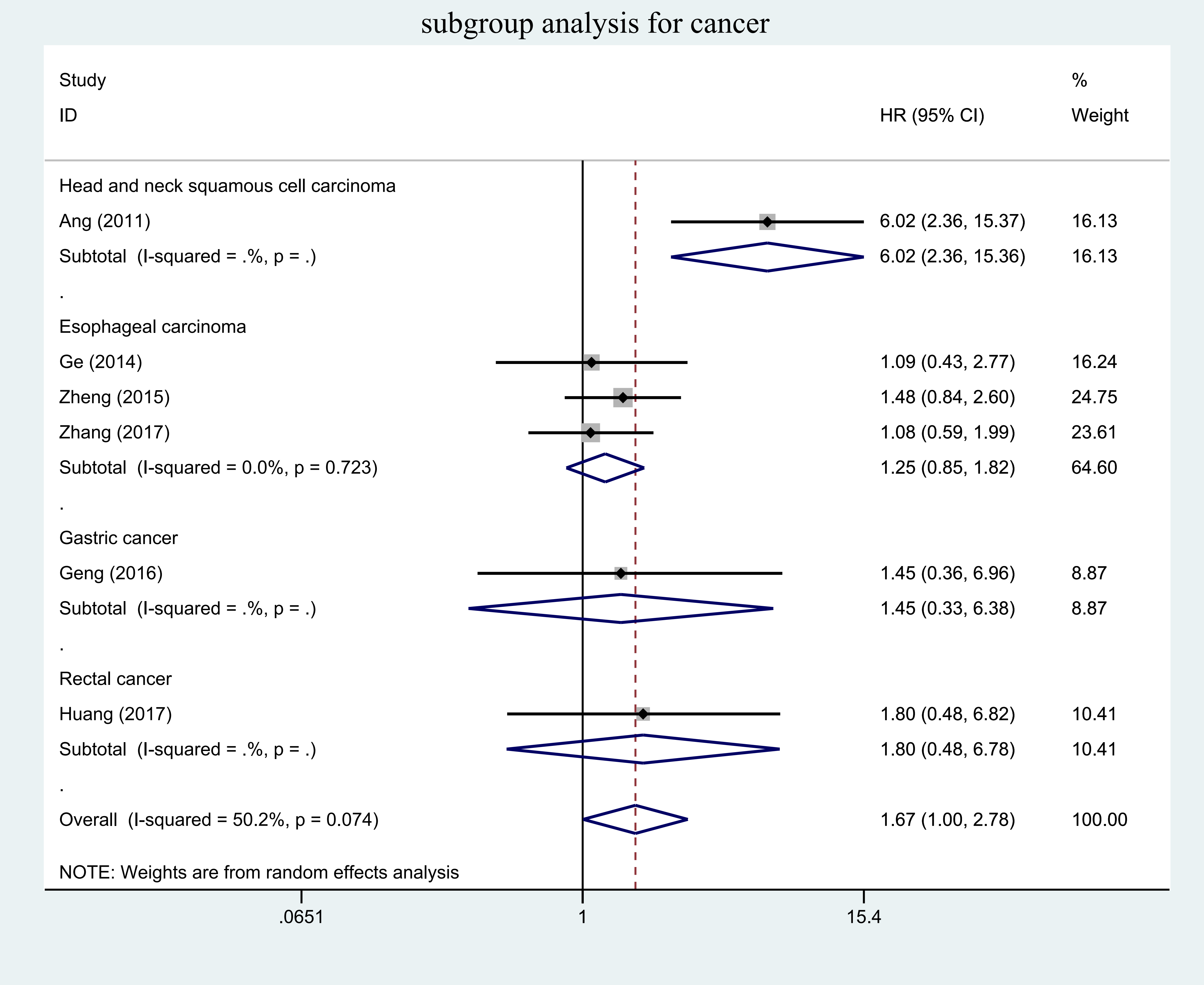
subgroup analysis for cancer
Study
ID
HR (95% CI)
%
Weight
Head and neck squamous cell carcinoma
Ang (2011)
6.02 (2.36, 15.37)
16.13
Subtotal (I-squared = .%, p = .)
6.02 (2.36, 15.36)
16.13
.
Esophageal carcinoma
Ge (2014)
1.09 (0.43, 2.77)
16.24
Zheng (2015)
1.48 (0.84, 2.60)
24.75
Zhang (2017)
1.08 (0.59, 1.99)
23.61
Subtotal (I-squared = 0.0%, p = 0.723)
1.25 (0.85, 1.82)
64.60
.
Gastric cancer
Geng (2016)
1.45 (0.36, 6.96)
8.87
Subtotal (I-squared = .%, p = .)
1.45 (0.33, 6.38)
8.87
.
Rectal cancer
Huang (2017)
1.80 (0.48, 6.82)
10.41
Subtotal (I-squared = .%, p = .)
1.80 (0.48, 6.78)
10.41
.
Overall (I-squared = 50.2%, p = 0.074)
1.67 (1.00, 2.78)
100.00
NOTE: Weights are from random effects analysis
.0651
1
15.4

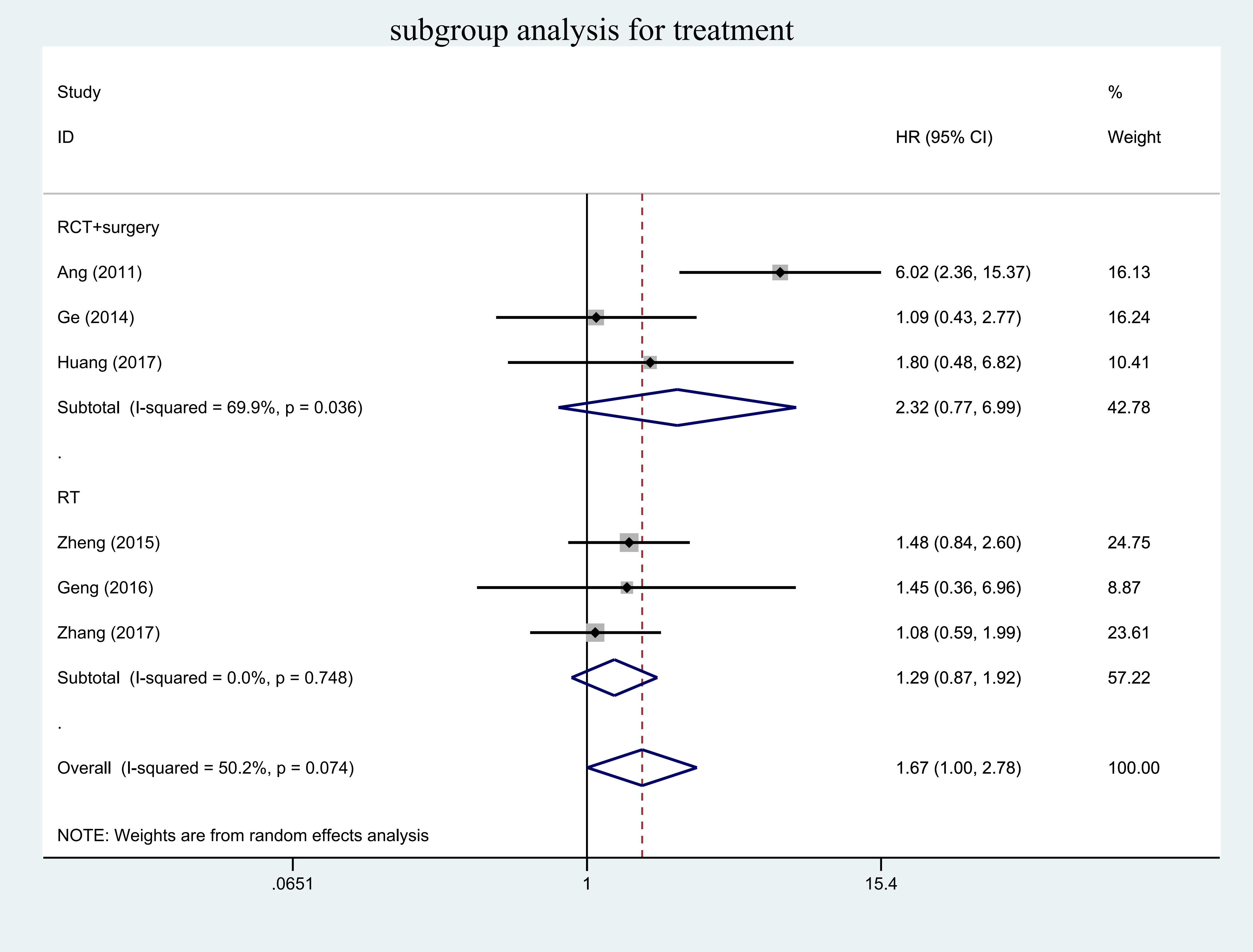
subgroup analysis for treatment
Study
ID
HR (95% CI)
%
Weight
RCT+surgery
Ang (2011)
6.02 (2.36, 15.37)
16.13
Ge (2014)
1.09 (0.43, 2.77)
16.24
Huang (2017)
1.80 (0.48, 6.82)
10.41
Subtotal (I-squared = 69.9%, p = 0.036)
2.32 (0.77, 6.99)
42.78
.
RT
Zheng (2015)
1.48 (0.84, 2.60)
24.75
Geng (2016)
1.45 (0.36, 6.96)
8.87
Zhang (2017)
1.08 (0.59, 1.99)
23.61
Subtotal (I-squared = 0.0%, p = 0.748)
1.29 (0.87, 1.92)
57.22
.
Overall (I-squared = 50.2%, p = 0.074)
1.67 (1.00, 2.78)
100.00
NOTE: Weights are from random effects analysis
.0651
1
15.4

TCGA data analysis

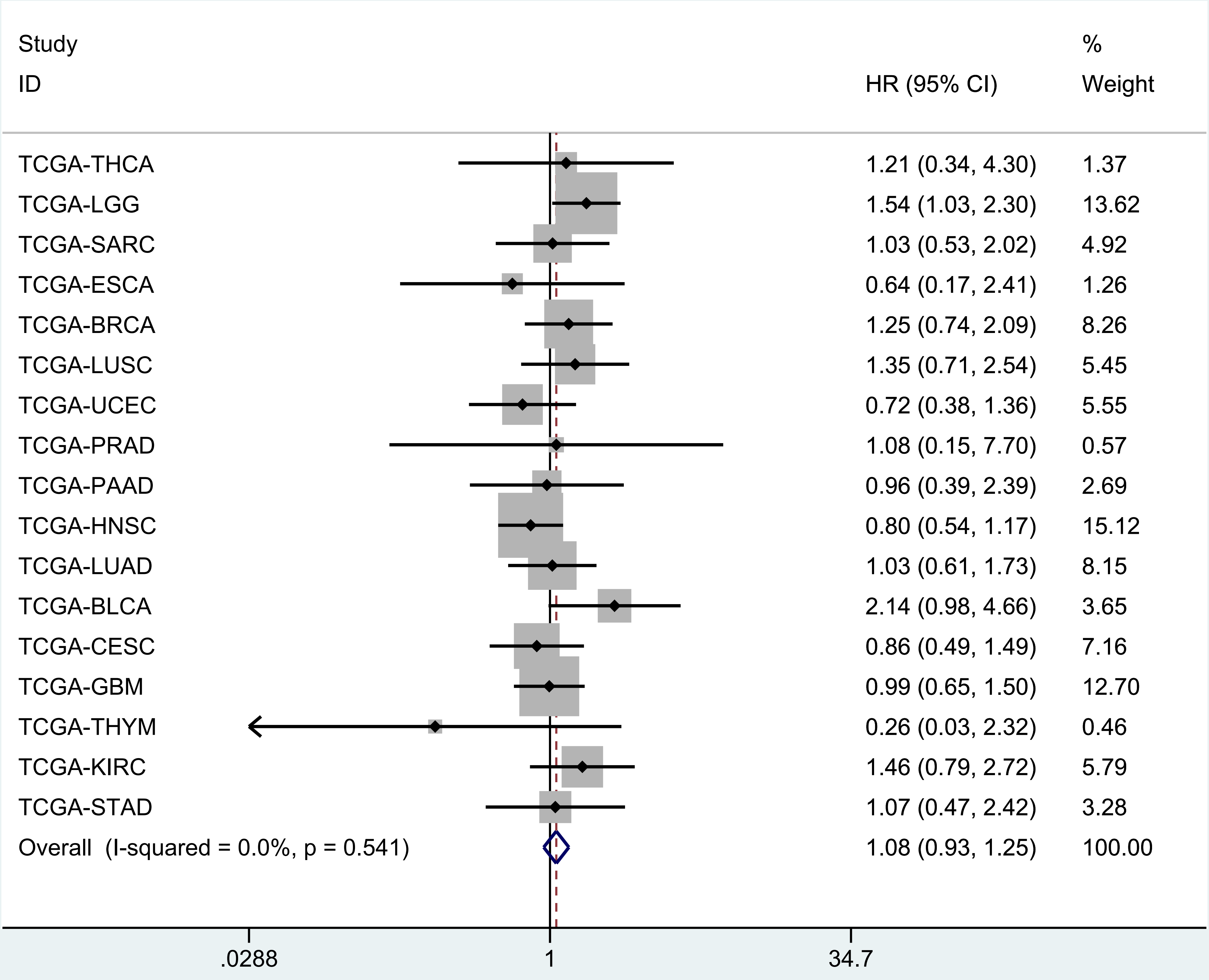

Supplementary File 3

Overall and subgroup analyses for association between XRCC1 rs25487 and treatment response. The genetic model includes heterozygote model (GA vs GG), homozygote model (AA vs GG), and dominant model (GA+AA vs GG). The subgroup includes types of cancer, cut-off, and treatment.

GA VS GG overall analysis

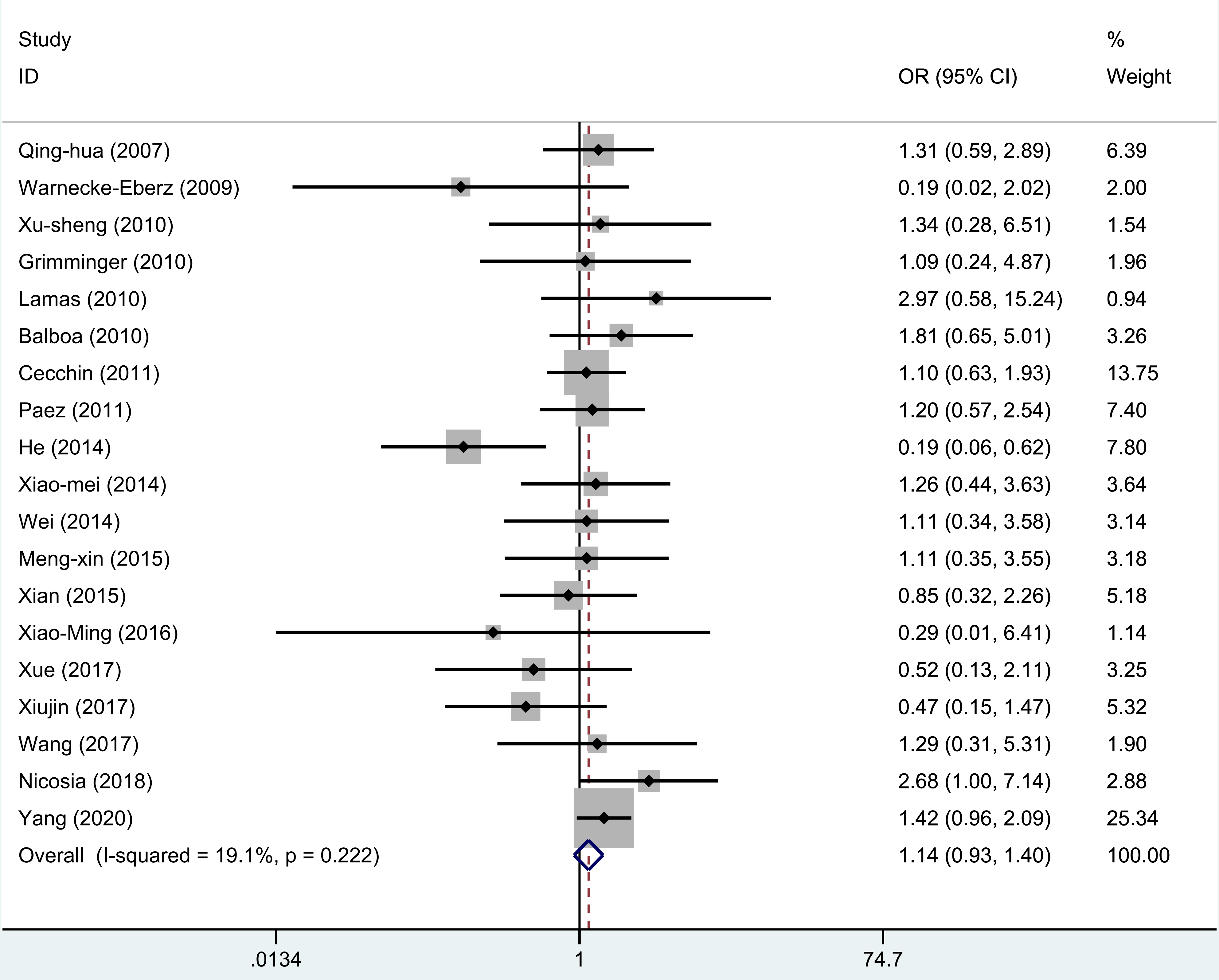

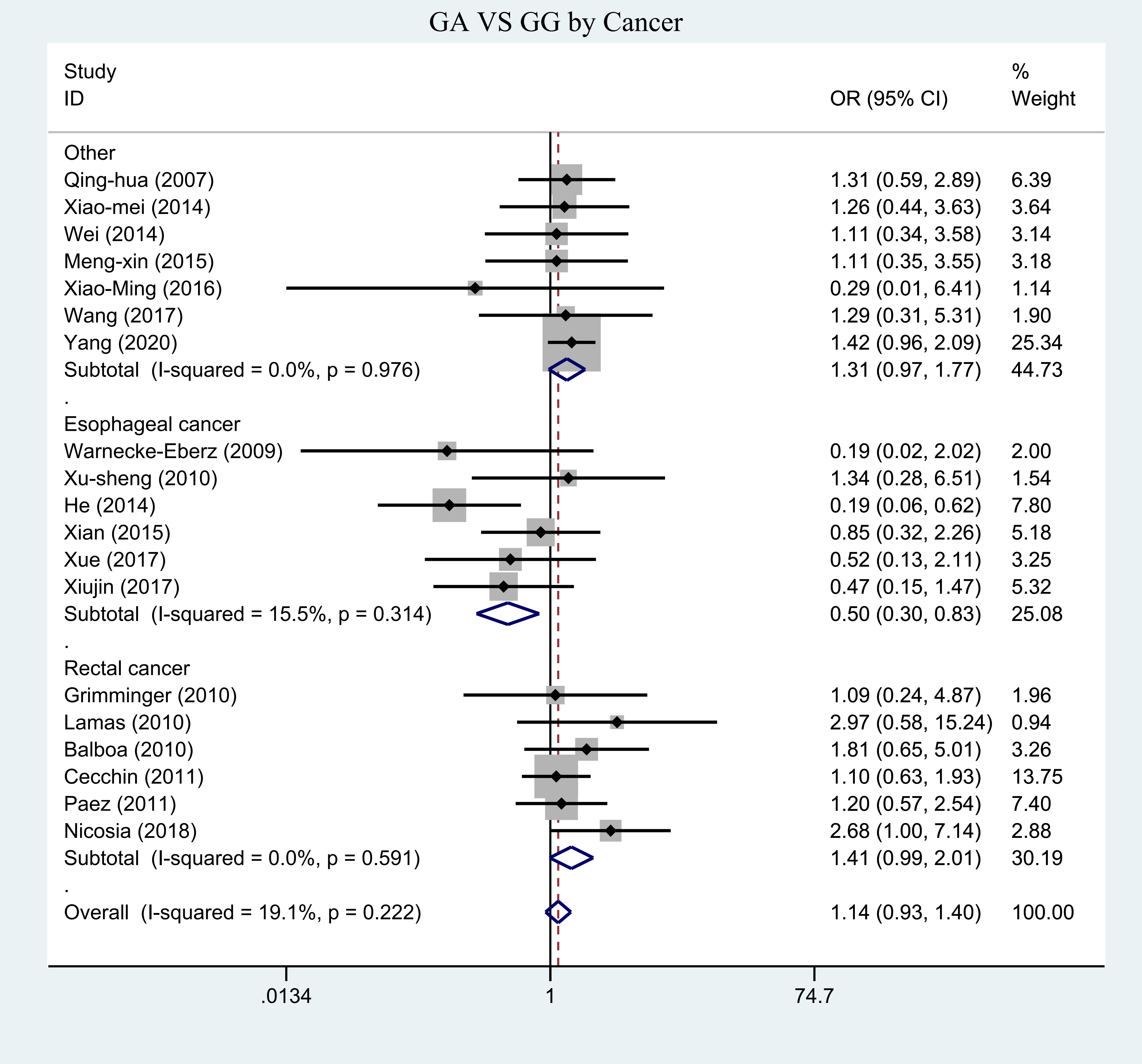
GA VS GG by Cancer
Study
ID
OR (95% CI)
%
Weight
Other
Qing-hua (2007) 1.31 (0.59, 2.89) 6.39
Xiao-mei (2014) 1.26 (0.44, 3.63) 3.64
Wei (2014) 1.11 (0.34, 3.58) 3.14
Meng-xin (2015) 1.11 (0.35, 3.55) 3.18
Xiao-Ming (2016) 0.29 (0.01, 6.41) 1.14
Wang (2017) 1.29 (0.31, 5.31) 1.90
Yang (2020) 1.42 (0.96, 2.09) 25.34
Subtotal (I-squared = 0.0%, p = 0.976) 1.31 (0.97, 1.77) 44.73
.
Esophageal cancer
Warnecke-Eberz (2009) 0.19 (0.02, 2.02) 2.00
Xu-sheng (2010) 1.34 (0.28, 6.51) 1.54
He (2014) 0.19 (0.06, 0.62) 7.80
Xian (2015) 0.85 (0.32, 2.26) 5.18
Xue (2017) 0.52 (0.13, 2.11) 3.25
Xiujin (2017) 0.47 (0.15, 1.47) 5.32
Subtotal (I-squared = 15.5%, p = 0.314) 0.50 (0.30, 0.83) 25.08
.
Rectal cancer
Grimminger (2010) 1.09 (0.24, 4.87) 1.96
Lamas (2010) 2.97 (0.58, 15.24) 0.94
Balboa (2010) 1.81 (0.65, 5.01) 3.26
Cecchin (2011) 1.10 (0.63, 1.93) 13.75
Paez (2011) 1.20 (0.57, 2.54) 7.40
Nicosia (2018) 2.68 (1.00, 7.14) 2.88
Subtotal (I-squared = 0.0%, p = 0.591) 1.41 (0.99, 2.01) 30.19
.
Overall (I-squared = 19.1%, p = 0.222) 1.14 (0.93, 1.40) 100.00
.0134
1
74.7

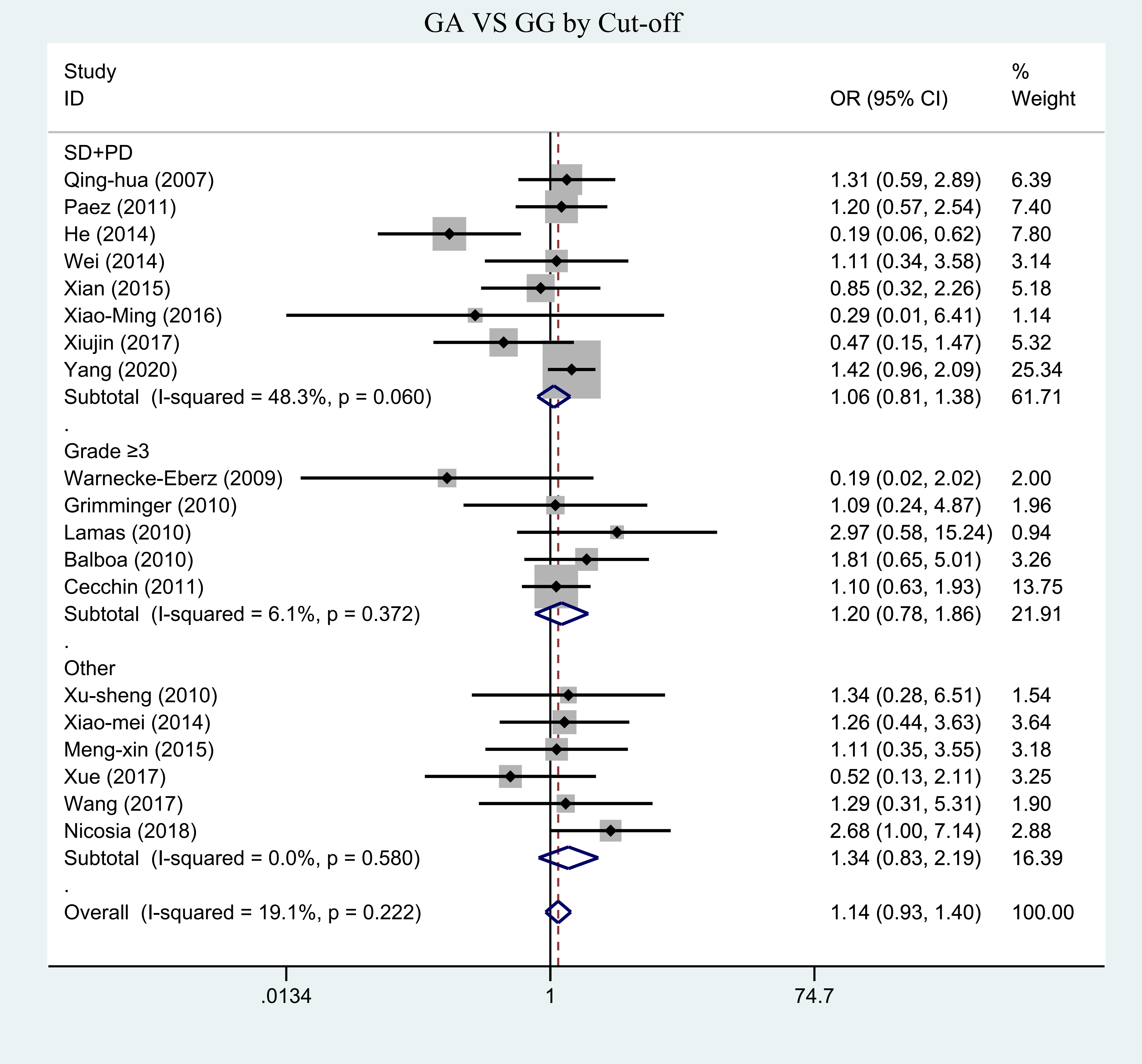
GA VS GG by Cut-off
Study
ID
OR (95% CI)
%
Weight
SD+PD
Qing-hua (2007) 1.31 (0.59, 2.89) 6.39
Paez (2011) 1.20 (0.57, 2.54) 7.40
He (2014) 0.19 (0.06, 0.62) 7.80
Wei (2014) 1.11 (0.34, 3.58) 3.14
Xian (2015) 0.85 (0.32, 2.26) 5.18
Xiao-Ming (2016) 0.29 (0.01, 6.41) 1.14
Xiujin (2017) 0.47 (0.15, 1.47) 5.32
Yang (2020) 1.42 (0.96, 2.09) 25.34
Subtotal (I-squared = 48.3%, p = 0.060) 1.06 (0.81, 1.38) 61.71
.
Grade ≥3
Warnecke-Eberz (2009) 0.19 (0.02, 2.02) 2.00
Grimminger (2010) 1.09 (0.24, 4.87) 1.96
Lamas (2010) 2.97 (0.58, 15.24) 0.94
Balboa (2010) 1.81 (0.65, 5.01) 3.26
Cecchin (2011) 1.10 (0.63, 1.93) 13.75
Subtotal (I-squared = 6.1%, p = 0.372) 1.20 (0.78, 1.86) 21.91
.
Other
Xu-sheng (2010) 1.34 (0.28, 6.51) 1.54
Xiao-mei (2014) 1.26 (0.44, 3.63) 3.64
Meng-xin (2015) 1.11 (0.35, 3.55) 3.18
Xue (2017) 0.52 (0.13, 2.11) 3.25
Wang (2017) 1.29 (0.31, 5.31) 1.90
Nicosia (2018) 2.68 (1.00, 7.14) 2.88
Subtotal (I-squared = 0.0%, p = 0.580) 1.34 (0.83, 2.19) 16.39
.
Overall (I-squared = 19.1%, p = 0.222) 1.14 (0.93, 1.40) 100.00
.0134
1
74.7

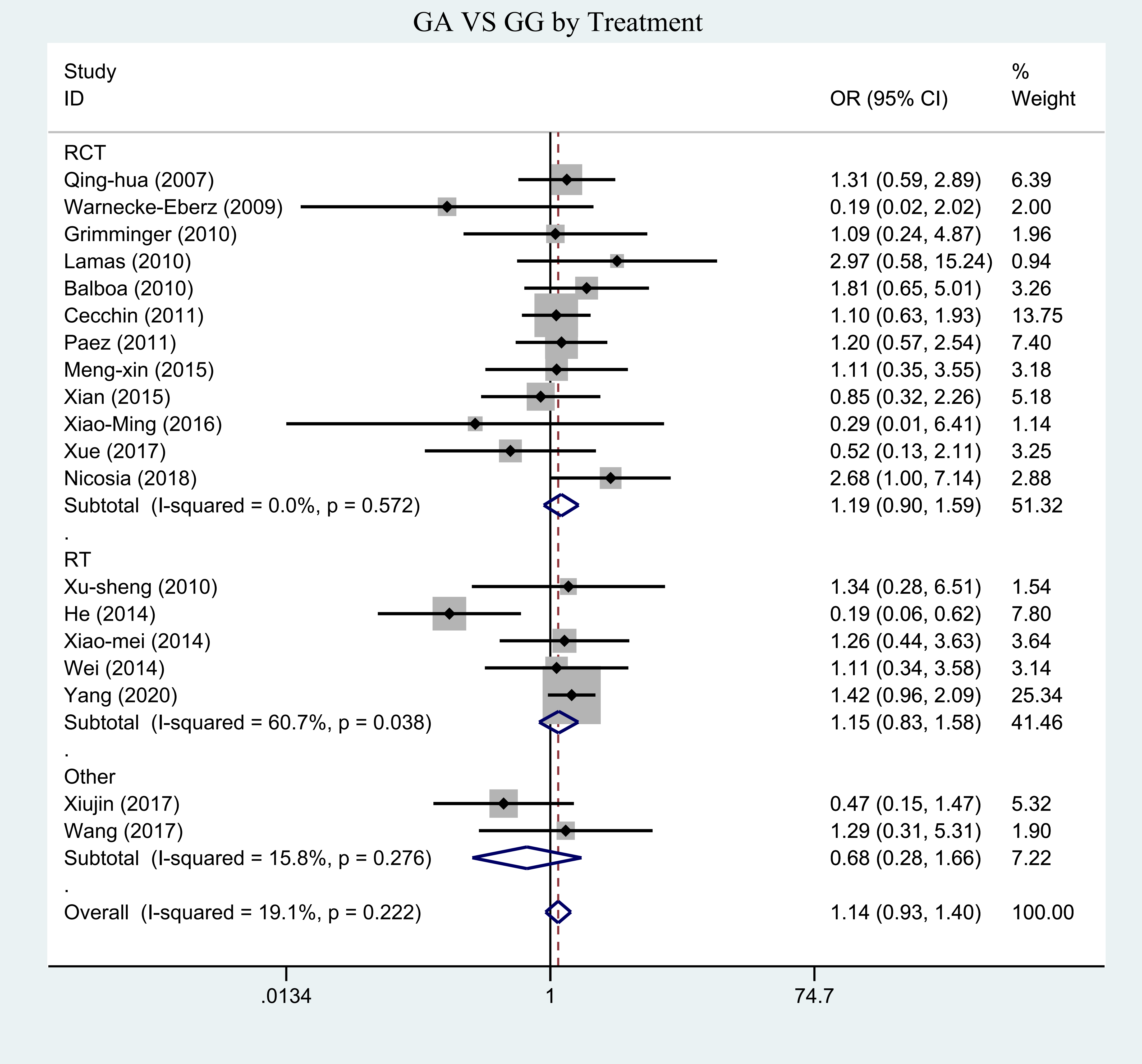
GA VS GG by Treatment
Study
ID
OR (95% CI)
%
Weight
RCT
Qing-hua (2007) 1.31 (0.59, 2.89) 6.39
Warnecke-Eberz (2009) 0.19 (0.02, 2.02) 2.00
Grimminger (2010) 1.09 (0.24, 4.87) 1.96
Lamas (2010) 2.97 (0.58, 15.24) 0.94
Balboa (2010) 1.81 (0.65, 5.01) 3.26
Cecchin (2011) 1.10 (0.63, 1.93) 13.75
Paez (2011) 1.20 (0.57, 2.54) 7.40
Meng-xin (2015) 1.11 (0.35, 3.55) 3.18
Xian (2015) 0.85 (0.32, 2.26) 5.18
Xiao-Ming (2016) 0.29 (0.01, 6.41) 1.14
Xue (2017) 0.52 (0.13, 2.11) 3.25
Nicosia (2018) 2.68 (1.00, 7.14) 2.88
Subtotal (I-squared = 0.0%, p = 0.572) 1.19 (0.90, 1.59) 51.32
.
RT
Xu-sheng (2010) 1.34 (0.28, 6.51) 1.54
He (2014) 0.19 (0.06, 0.62) 7.80
Xiao-mei (2014) 1.26 (0.44, 3.63) 3.64
Wei (2014) 1.11 (0.34, 3.58) 3.14
Yang (2020) 1.42 (0.96, 2.09) 25.34
Subtotal (I-squared = 60.7%, p = 0.038) 1.15 (0.83, 1.58) 41.46
.
Other
Xiujin (2017) 0.47 (0.15, 1.47) 5.32
Wang (2017) 1.29 (0.31, 5.31) 1.90
Subtotal (I-squared = 15.8%, p = 0.276) 0.68 (0.28, 1.66) 7.22
.
Overall (I-squared = 19.1%, p = 0.222) 1.14 (0.93, 1.40) 100.00
.0134
1
74.7

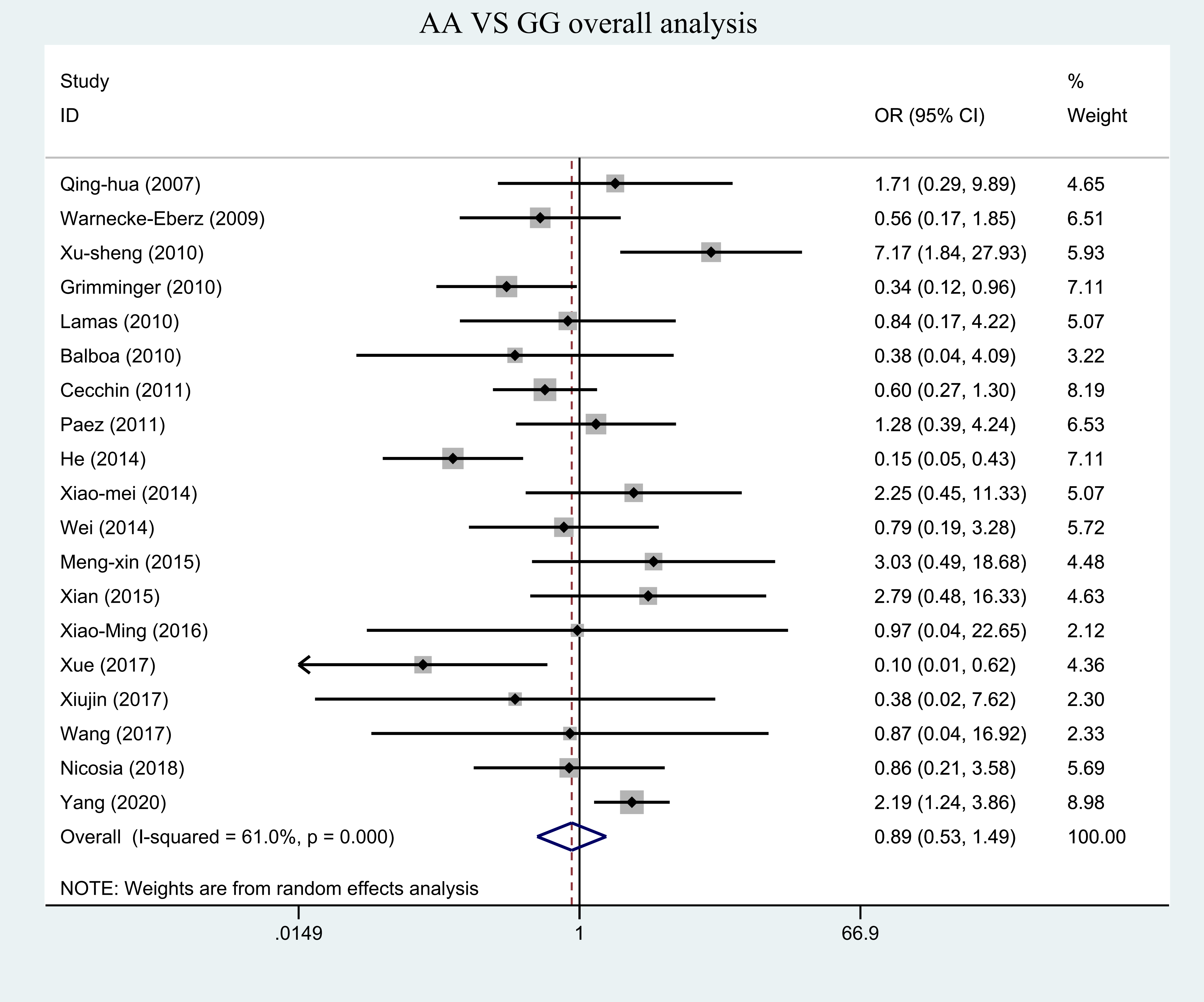
AA VS GG overall analysis
Study
ID
OR (95% CI)
%
Weight
Qing-hua (2007)
1.71 (0.29, 9.89)
4.65
Warnecke-Eberz (2009)
0.56 (0.17, 1.85)
6.51
Xu-sheng (2010)
7.17 (1.84, 27.93)
5.93
Grimminger (2010)
0.34 (0.12, 0.96)
7.11
Lamas (2010)
0.84 (0.17, 4.22)
5.07
Balboa (2010)
0.38 (0.04, 4.09)
3.22
Cecchin (2011)
0.60 (0.27, 1.30)
8.19
Paez (2011)
1.28 (0.39, 4.24)
6.53
He (2014)
0.15 (0.05, 0.43)
7.11
Xiao-mei (2014)
2.25 (0.45, 11.33)
5.07
Wei (2014)
0.79 (0.19, 3.28)
5.72
Meng-xin (2015)
3.03 (0.49, 18.68)
4.48
Xian (2015)
2.79 (0.48, 16.33)
4.63
Xiao-Ming (2016)
0.97 (0.04, 22.65)
2.12
Xue (2017)
0.10 (0.01, 0.62)
4.36
Xiujin (2017)
0.38 (0.02, 7.62)
2.30
Wang (2017)
0.87 (0.04, 16.92)
2.33
Nicosia (2018)
0.86 (0.21, 3.58)
5.69
Yang (2020)
2.19 (1.24, 3.86)
8.98
Overall (I-squared = 61.0%, p = 0.000)
0.89 (0.53, 1.49)
100.00
NOTE: Weights are from random effects analysis
.0149
1
66.9

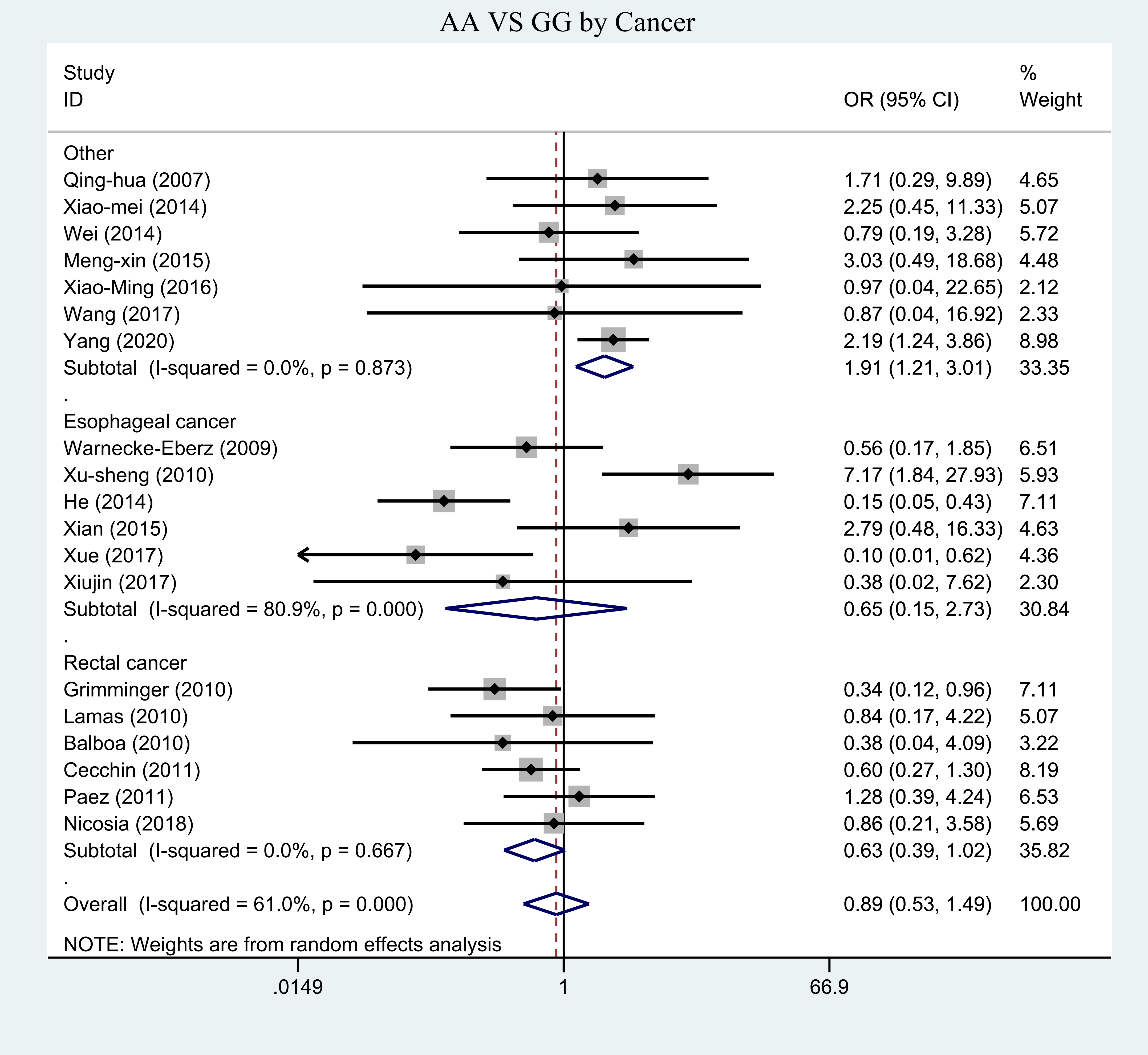
AA VS GG by Cancer
Study
ID
OR (95% CI)
%
Weight
Other
Qing-hua (2007) 1.71 (0.29, 9.89) 4.65
Xiao-mei (2014) 2.25 (0.45, 11.33) 5.07
Wei (2014) 0.79 (0.19, 3.28) 5.72
Meng-xin (2015) 3.03 (0.49, 18.68) 4.48
Xiao-Ming (2016) 0.97 (0.04, 22.65) 2.12
Wang (2017) 0.87 (0.04, 16.92) 2.33
Yang (2020) 2.19 (1.24, 3.86) 8.98
Subtotal (I-squared = 0.0%, p = 0.873) 1.91 (1.21, 3.01) 33.35
.
Esophageal cancer
Warnecke-Eberz (2009) 0.56 (0.17, 1.85) 6.51
Xu-sheng (2010) 7.17 (1.84, 27.93) 5.93
He (2014) 0.15 (0.05, 0.43) 7.11
Xian (2015) 2.79 (0.48, 16.33) 4.63
Xue (2017) 0.10 (0.01, 0.62) 4.36
Xiujin (2017) 0.38 (0.02, 7.62) 2.30
Subtotal (I-squared = 80.9%, p = 0.000) 0.65 (0.15, 2.73) 30.84
.
Rectal cancer
Grimminger (2010) 0.34 (0.12, 0.96) 7.11
Lamas (2010) 0.84 (0.17, 4.22) 5.07
Balboa (2010) 0.38 (0.04, 4.09) 3.22
Cecchin (2011) 0.60 (0.27, 1.30) 8.19
Paez (2011) 1.28 (0.39, 4.24) 6.53
Nicosia (2018) 0.86 (0.21, 3.58) 5.69
Subtotal (I-squared = 0.0%, p = 0.667) 0.63 (0.39, 1.02) 35.82
.
Overall (I-squared = 61.0%, p = 0.000) 0.89 (0.53, 1.49) 100.00
NOTE: Weights are from random effects analysis
.0149
1
66.9

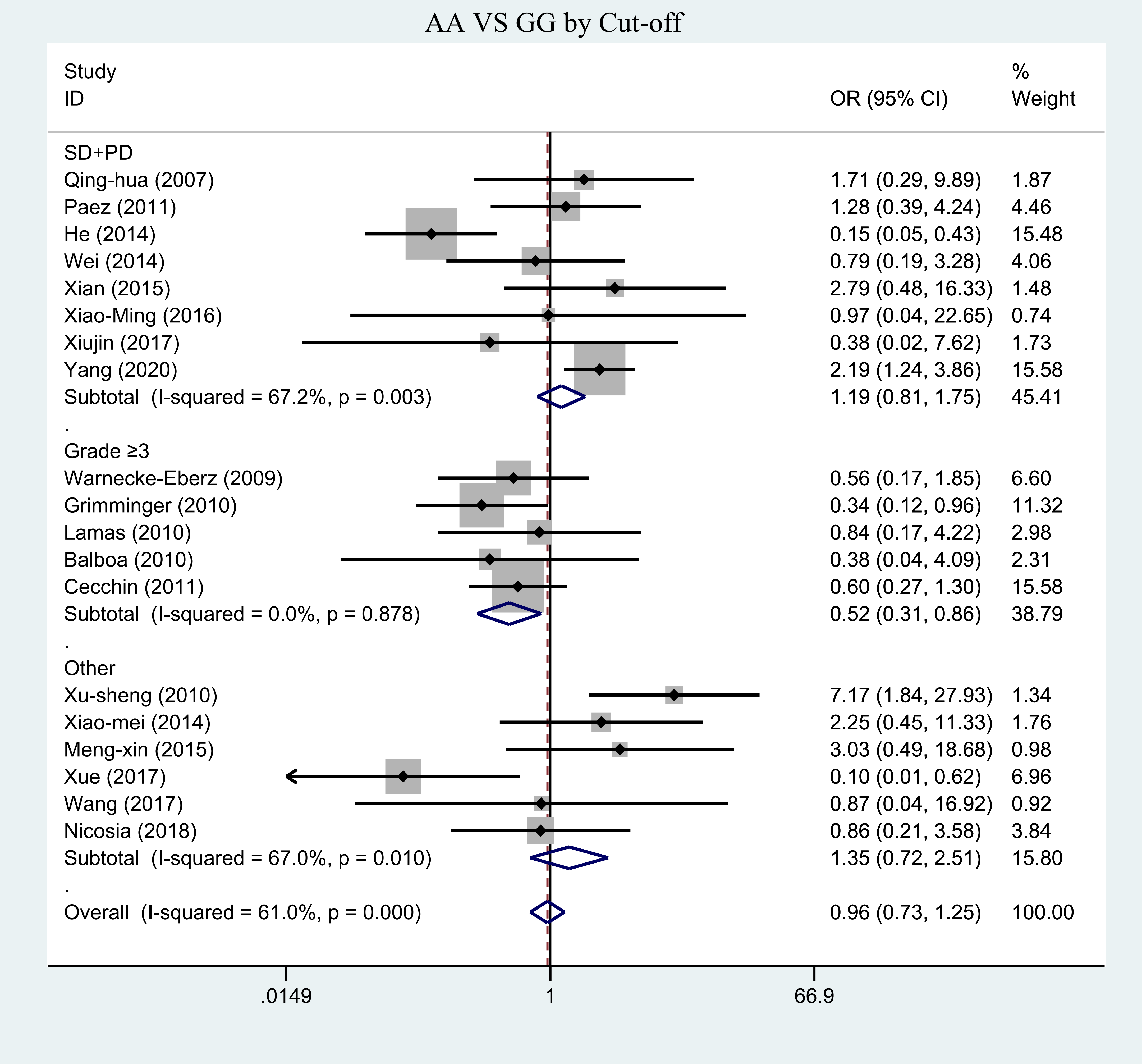
AA VS GG by Cut-off
Study
ID
OR (95% CI)
%
Weight
SD+PD
Qing-hua (2007) 1.71 (0.29, 9.89) 1.87
Paez (2011) 1.28 (0.39, 4.24) 4.46
He (2014) 0.15 (0.05, 0.43) 15.48
Wei (2014) 0.79 (0.19, 3.28) 4.06
Xian (2015) 2.79 (0.48, 16.33) 1.48
Xiao-Ming (2016) 0.97 (0.04, 22.65) 0.74
Xiujin (2017) 0.38 (0.02, 7.62) 1.73
Yang (2020) 2.19 (1.24, 3.86) 15.58
Subtotal (I-squared = 67.2%, p = 0.003) 1.19 (0.81, 1.75) 45.41
.
Grade ≥3
Warnecke-Eberz (2009) 0.56 (0.17, 1.85) 6.60
Grimminger (2010) 0.34 (0.12, 0.96) 11.32
Lamas (2010) 0.84 (0.17, 4.22) 2.98
Balboa (2010) 0.38 (0.04, 4.09) 2.31
Cecchin (2011) 0.60 (0.27, 1.30) 15.58
Subtotal (I-squared = 0.0%, p = 0.878) 0.52 (0.31, 0.86) 38.79
.
Other
Xu-sheng (2010) 7.17 (1.84, 27.93) 1.34
Xiao-mei (2014) 2.25 (0.45, 11.33) 1.76
Meng-xin (2015) 3.03 (0.49, 18.68) 0.98
Xue (2017) 0.10 (0.01, 0.62) 6.96
Wang (2017) 0.87 (0.04, 16.92) 0.92
Nicosia (2018) 0.86 (0.21, 3.58) 3.84
Subtotal (I-squared = 67.0%, p = 0.010) 1.35 (0.72, 2.51) 15.80
.
Overall (I-squared = 61.0%, p = 0.000) 0.96 (0.73, 1.25) 100.00
.0149
1
66.9

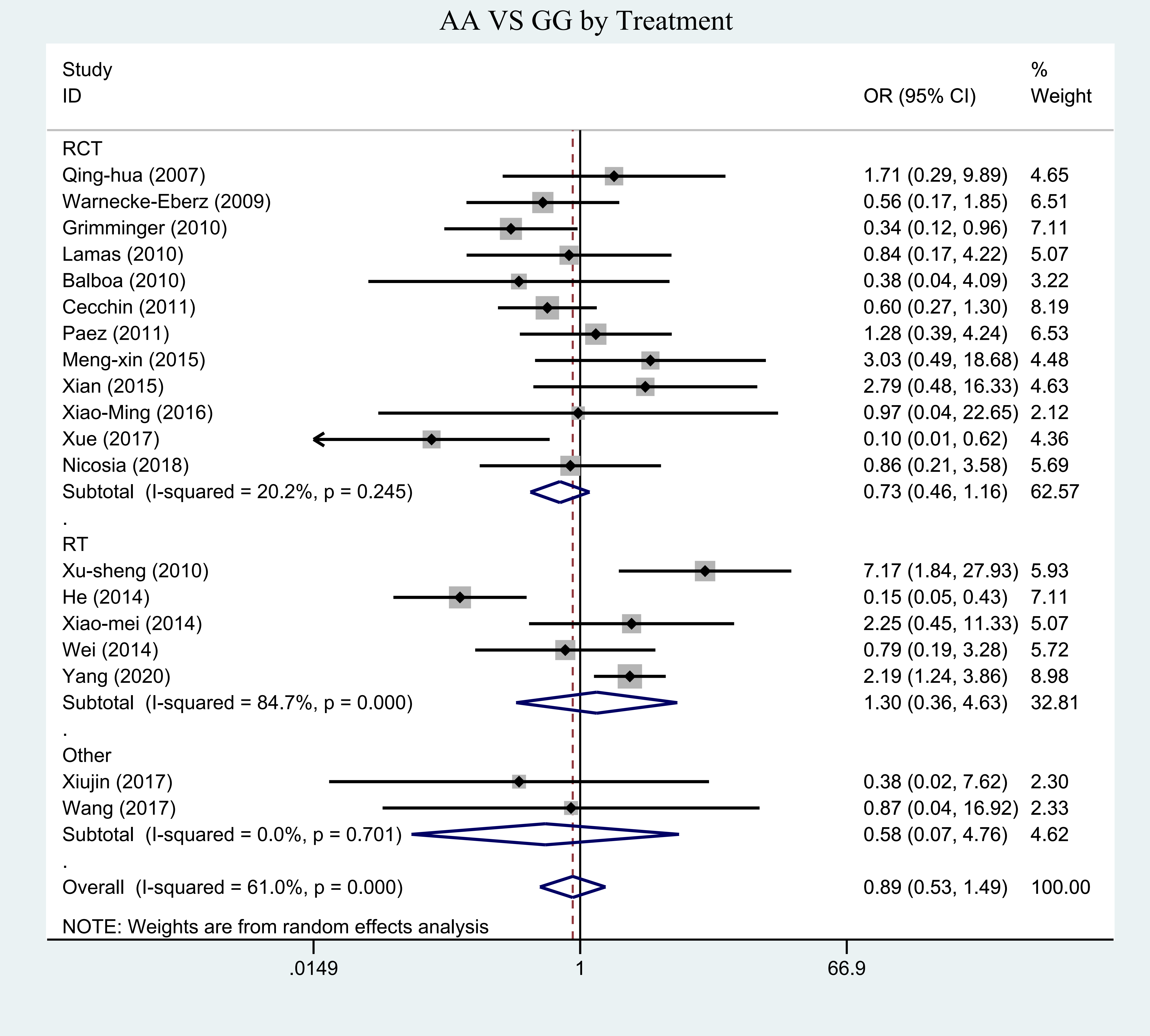
AA VS GG by Treatment
Study
ID
OR (95% CI)
%
Weight
RCT
Qing-hua (2007) 1.71 (0.29, 9.89) 4.65
Warnecke-Eberz (2009) 0.56 (0.17, 1.85) 6.51
Grimminger (2010) 0.34 (0.12, 0.96) 7.11
Lamas (2010) 0.84 (0.17, 4.22) 5.07
Balboa (2010) 0.38 (0.04, 4.09) 3.22
Cecchin (2011) 0.60 (0.27, 1.30) 8.19
Paez (2011) 1.28 (0.39, 4.24) 6.53
Meng-xin (2015) 3.03 (0.49, 18.68) 4.48
Xian (2015) 2.79 (0.48, 16.33) 4.63
Xiao-Ming (2016) 0.97 (0.04, 22.65) 2.12
Xue (2017) 0.10 (0.01, 0.62) 4.36
Nicosia (2018) 0.86 (0.21, 3.58) 5.69
Subtotal (I-squared = 20.2%, p = 0.245) 0.73 (0.46, 1.16) 62.57
RT
Xu-sheng (2010) 7.17 (1.84, 27.93) 5.93
He (2014) 0.15 (0.05, 0.43) 7.11
Xiao-mei (2014) 2.25 (0.45, 11.33) 5.07
Wei (2014) 0.79 (0.19, 3.28) 5.72
Yang (2020) 2.19 (1.24, 3.86) 8.98
Subtotal (I-squared = 84.7%, p = 0.000) 1.30 (0.36, 4.63) 32.81
Other
Xiujin (2017) 0.38 (0.02, 7.62) 2.30
Wang (2017) 0.87 (0.04, 16.92) 2.33
Subtotal (I-squared = 0.0%, p = 0.701) 0.58 (0.07, 4.76) 4.62
Overall (I-squared = 61.0%, p = 0.000) 0.89 (0.53, 1.49) 100.00
NOTE: Weights are from random effects analysis
.0149
1
66.9

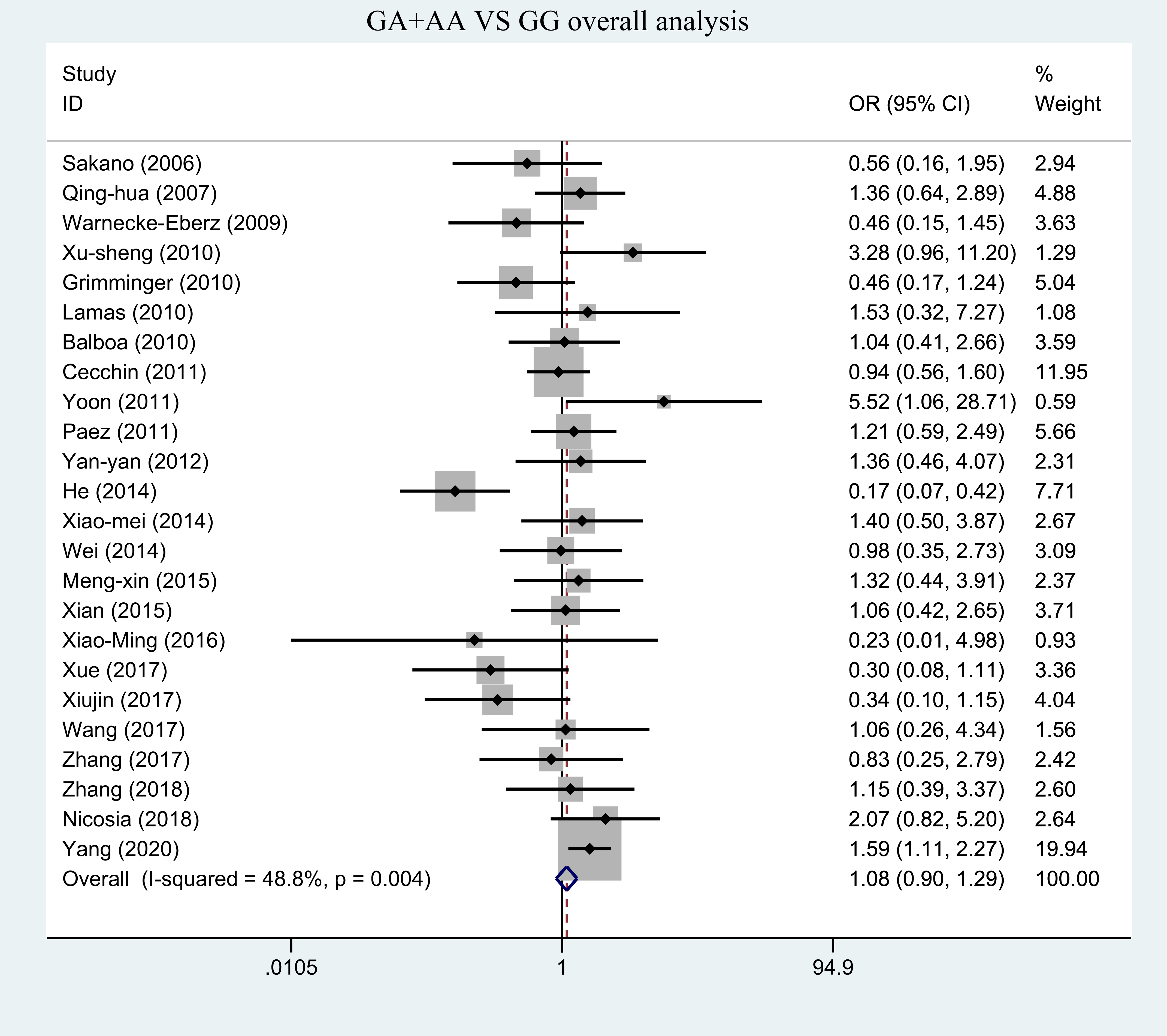

GA+AA VS GG overall analysis
Study ID
OR (95% CI)
% Weight
Sakano (2006) 0.56 (0.16, 1.95) 2.94
Qing-hua (2007) 1.36 (0.64, 2.89) 4.88
Warnecke-Eberz (2009) 0.46 (0.15, 1.45) 3.63
Xu-sheng (2010) 3.28 (0.96, 11.20) 1.29
Grimminger (2010) 0.46 (0.17, 1.24) 5.04
Lamas (2010) 1.53 (0.32, 7.27) 1.08
Balboa (2010) 1.04 (0.41, 2.66) 3.59
Cecchin (2011) 0.94 (0.56, 1.60) 11.95
Yoon (2011) 5.52 (1.06, 28.71) 0.59
Paez (2011) 1.21 (0.59, 2.49) 5.66
Yan-yan (2012) 1.36 (0.46, 4.07) 2.31
He (2014) 0.17 (0.07, 0.42) 7.71
Xiao-mei (2014) 1.40 (0.50, 3.87) 2.67
Wei (2014) 0.98 (0.35, 2.73) 3.09
Meng-xin (2015) 1.32 (0.44, 3.91) 2.37
Xian (2015) 1.06 (0.42, 2.65) 3.71
Xiao-Ming (2016) 0.23 (0.01, 4.98) 0.93
Xue (2017) 0.30 (0.08, 1.11) 3.36
Xiujin (2017) 0.34 (0.10, 1.15) 4.04
Wang (2017) 1.06 (0.26, 4.34) 1.56
Zhang (2017) 0.83 (0.25, 2.79) 2.42
Zhang (2018) 1.15 (0.39, 3.37) 2.60
Nicosia (2018) 2.07 (0.82, 5.20) 2.64
Yang (2020) 1.59 (1.11, 2.27) 19.94
Overall (I-squared = 48.8%, p = 0.004) 1.08 (0.90, 1.29) 100.00
.0105
1
94.9

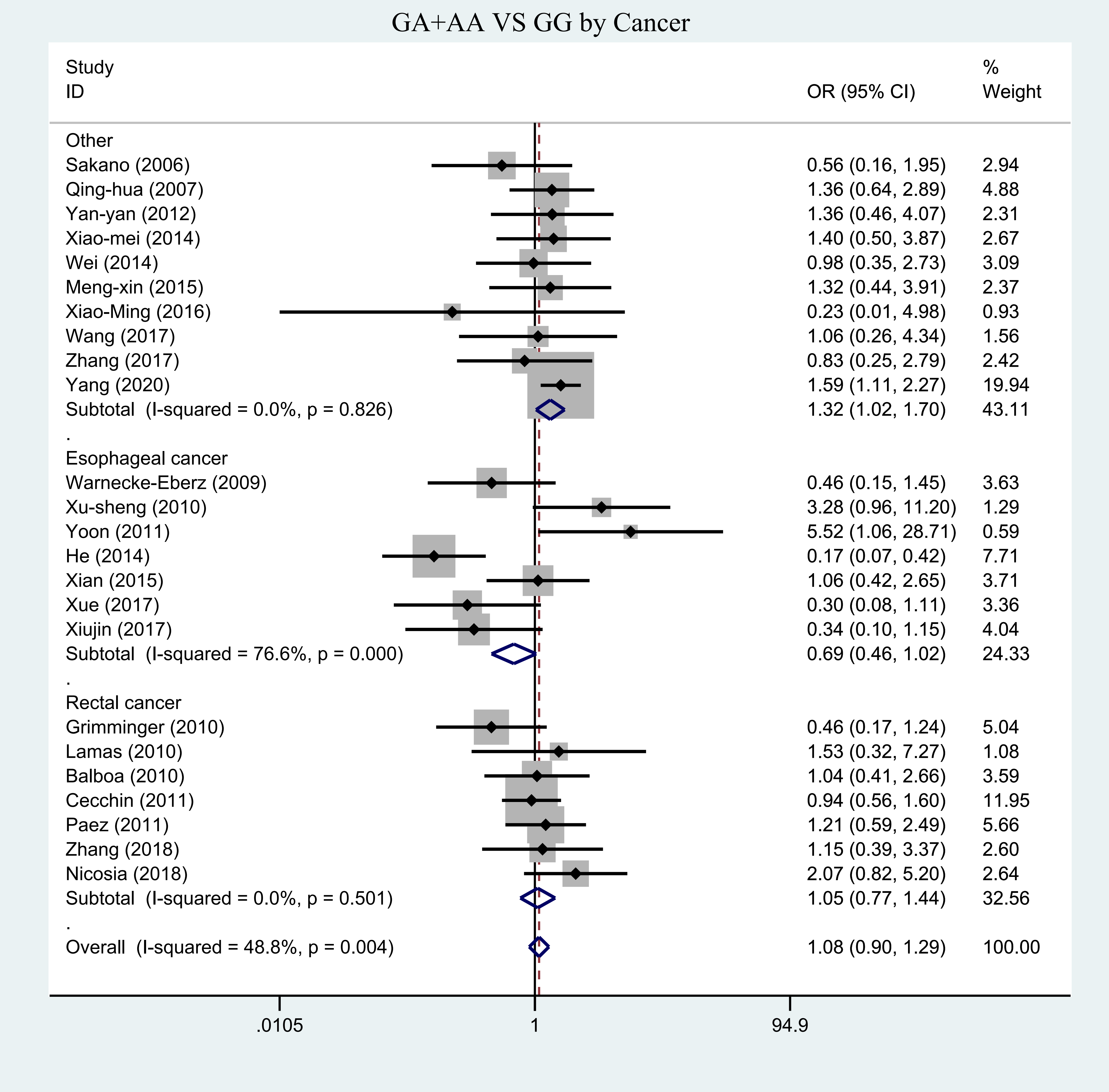
GA+AA VS GG by Cancer
Study ID
OR (95% CI)
% Weight
Other
Sakano (2006) 0.56 (0.16, 1.95) 2.94
Qing-hua (2007) 1.36 (0.64, 2.89) 4.88
Yan-yan (2012) 1.36 (0.46, 4.07) 2.31
Xiao-mei (2014) 1.40 (0.50, 3.87) 2.67
Wei (2014) 0.98 (0.35, 2.73) 3.09
Meng-xin (2015) 1.32 (0.44, 3.91) 2.37
Xiao-Ming (2016) 0.23 (0.01, 4.98) 0.93
Wang (2017) 1.06 (0.26, 4.34) 1.56
Zhang (2017) 0.83 (0.25, 2.79) 2.42
Yang (2020) 1.59 (1.11, 2.27) 19.94
Subtotal (I-squared = 0.0%, p = 0.826) 1.32 (1.02, 1.70) 43.11
.
Esophageal cancer
Warnecke-Eberz (2009) 0.46 (0.15, 1.45) 3.63
Xu-sheng (2010) 3.28 (0.96, 11.20) 1.29
Yoon (2011) 5.52 (1.06, 28.71) 0.59
He (2014) 0.17 (0.07, 0.42) 7.71
Xian (2015) 1.06 (0.42, 2.65) 3.71
Xue (2017) 0.30 (0.08, 1.11) 3.36
Xiujin (2017) 0.34 (0.10, 1.15) 4.04
Subtotal (I-squared = 76.6%, p = 0.000) 0.69 (0.46, 1.02) 24.33
.
Rectal cancer
Grimminger (2010) 0.46 (0.17, 1.24) 5.04
Lamas (2010) 1.53 (0.32, 7.27) 1.08
Balboa (2010) 1.04 (0.41, 2.66) 3.59
Cecchin (2011) 0.94 (0.56, 1.60) 11.95
Paez (2011) 1.21 (0.59, 2.49) 5.66
Zhang (2018) 1.15 (0.39, 3.37) 2.60
Nicosia (2018) 2.07 (0.82, 5.20) 2.64
Subtotal (I-squared = 0.0%, p = 0.501) 1.05 (0.77, 1.44) 32.56
.
Overall (I-squared = 48.8%, p = 0.004) 1.08 (0.90, 1.29) 100.00
.0105
1
94.9

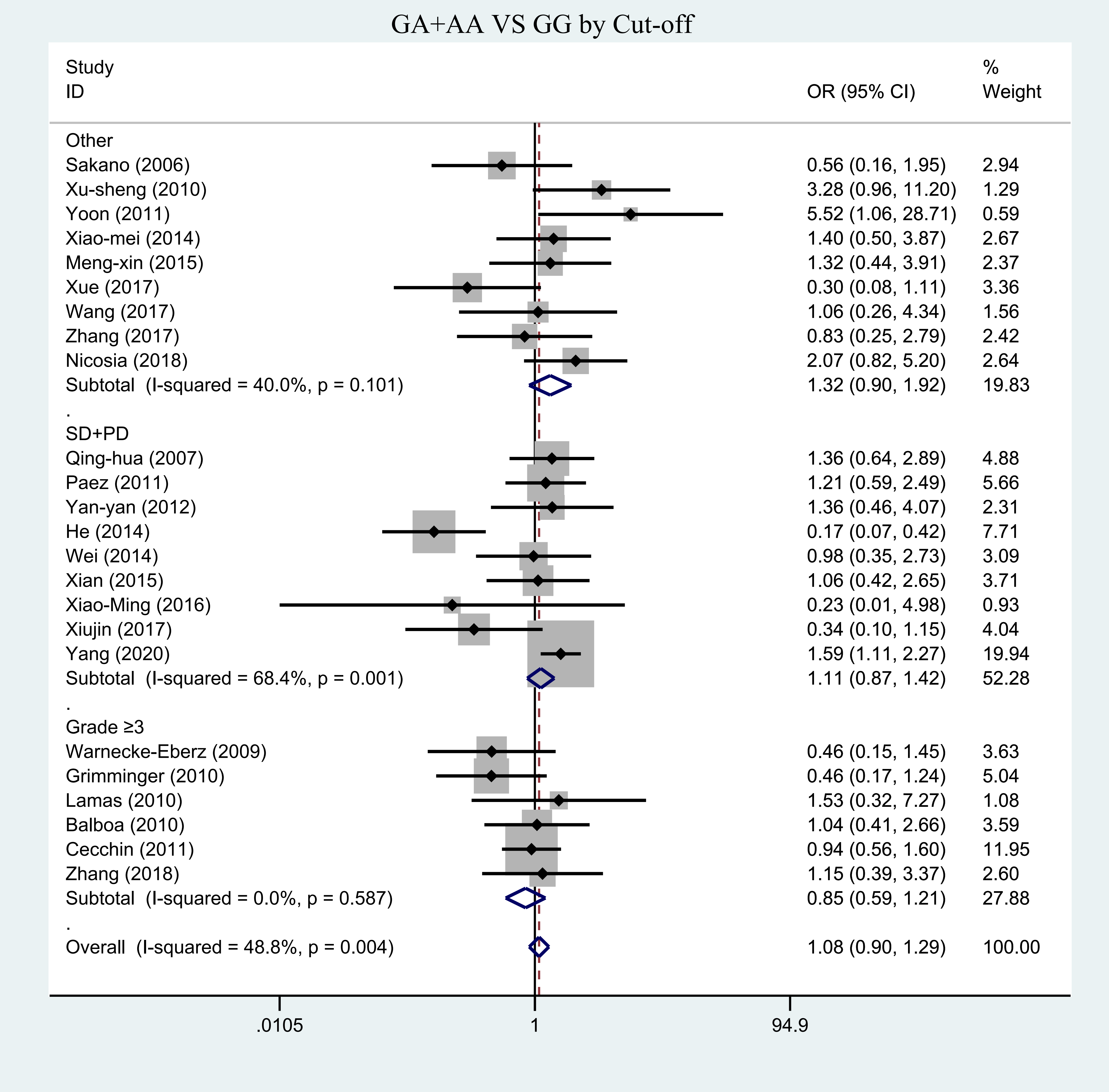
GA+AA VS GG by Cut-off
Study
ID
OR (95% CI)
%
Weight
Other
Sakano (2006) 0.56 (0.16, 1.95) 2.94
Xu-sheng (2010) 3.28 (0.96, 11.20) 1.29
Yoon (2011) 5.52 (1.06, 28.71) 0.59
Xiao-mei (2014) 1.40 (0.50, 3.87) 2.67
Meng-xin (2015) 1.32 (0.44, 3.91) 2.37
Xue (2017) 0.30 (0.08, 1.11) 3.36
Wang (2017) 1.06 (0.26, 4.34) 1.56
Zhang (2017) 0.83 (0.25, 2.79) 2.42
Nicosia (2018) 2.07 (0.82, 5.20) 2.64
Subtotal (I-squared = 40.0%, p = 0.101) 1.32 (0.90, 1.92) 19.83
.
SD+PD
Qing-hua (2007) 1.36 (0.64, 2.89) 4.88
Paez (2011) 1.21 (0.59, 2.49) 5.66
Yan-yan (2012) 1.36 (0.46, 4.07) 2.31
He (2014) 0.17 (0.07, 0.42) 7.71
Wei (2014) 0.98 (0.35, 2.73) 3.09
Xian (2015) 1.06 (0.42, 2.65) 3.71
Xiao-Ming (2016) 0.23 (0.01, 4.98) 0.93
Xiujin (2017) 0.34 (0.10, 1.15) 4.04
Yang (2020) 1.59 (1.11, 2.27) 19.94
Subtotal (I-squared = 68.4%, p = 0.001) 1.11 (0.87, 1.42) 52.28
.
Grade ≥3
Warnecke-Eberz (2009) 0.46 (0.15, 1.45) 3.63
Grimminger (2010) 0.46 (0.17, 1.24) 5.04
Lamas (2010) 1.53 (0.32, 7.27) 1.08
Balboa (2010) 1.04 (0.41, 2.66) 3.59
Cecchin (2011) 0.94 (0.56, 1.60) 11.95
Zhang (2018) 1.15 (0.39, 3.37) 2.60
Subtotal (I-squared = 0.0%, p = 0.587) 0.85 (0.59, 1.21) 27.88
.
Overall (I-squared = 48.8%, p = 0.004) 1.08 (0.90, 1.29) 100.00
.0105
1
94.9

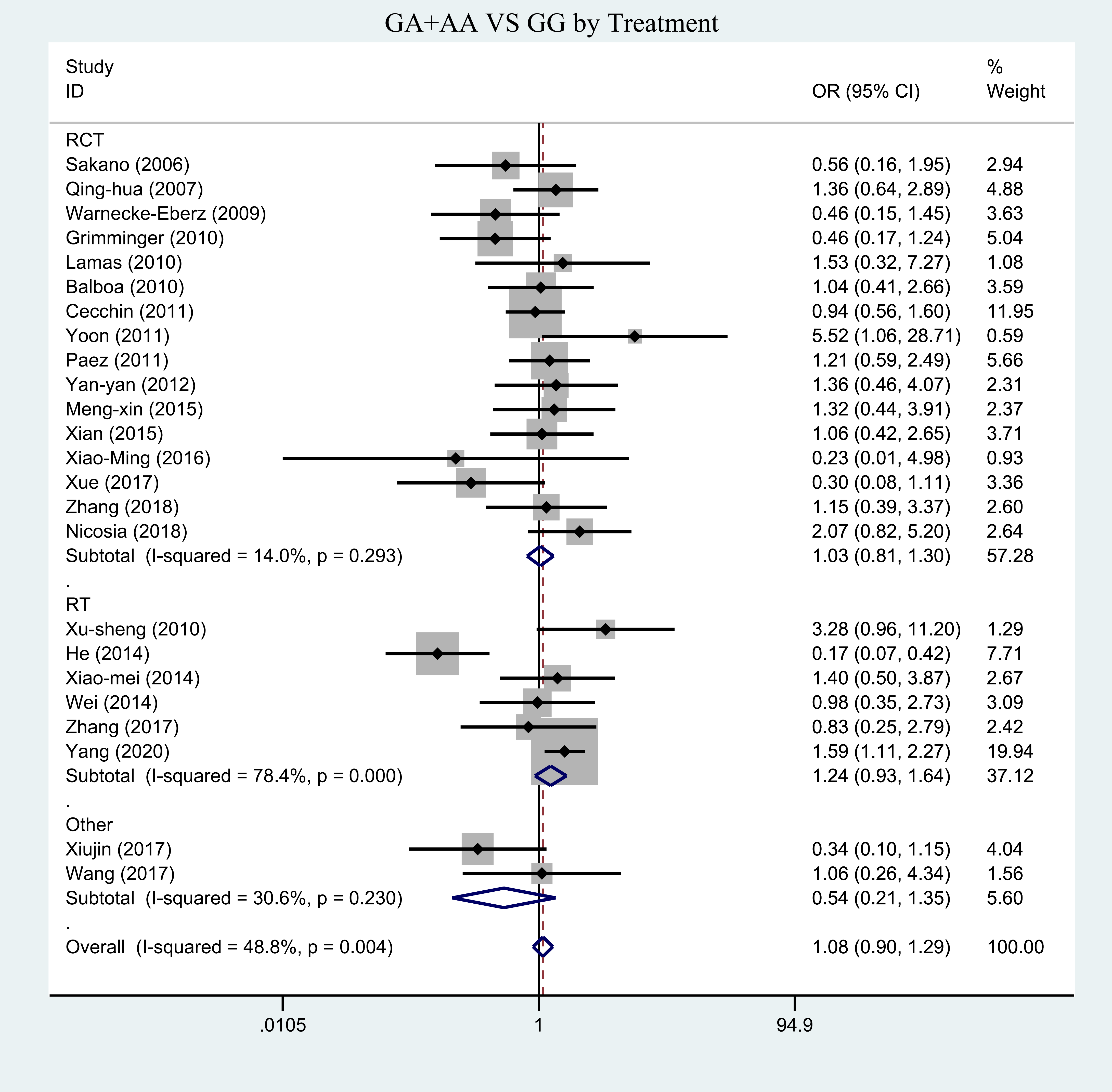
GA+AA VS GG by Treatment
Study
ID
OR (95% CI)
%
Weight
RCT
Sakano (2006) 0.56 (0.16, 1.95) 2.94
Qing-hua (2007) 1.36 (0.64, 2.89) 4.88
Warnecke-Eberz (2009) 0.46 (0.15, 1.45) 3.63
Grimminger (2010) 0.46 (0.17, 1.24) 5.04
Lamas (2010) 1.53 (0.32, 7.27) 1.08
Balboa (2010) 1.04 (0.41, 2.66) 3.59
Cecchin (2011) 0.94 (0.56, 1.60) 11.95
Yoon (2011) 5.52 (1.06, 28.71) 0.59
Paez (2011) 1.21 (0.59, 2.49) 5.66
Yan-yan (2012) 1.36 (0.46, 4.07) 2.31
Meng-xin (2015) 1.32 (0.44, 3.91) 2.37
Xian (2015) 1.06 (0.42, 2.65) 3.71
Xiao-Ming (2016) 0.23 (0.01, 4.98) 0.93
Xue (2017) 0.30 (0.08, 1.11) 3.36
Zhang (2018) 1.15 (0.39, 3.37) 2.60
Nicosia (2018) 2.07 (0.82, 5.20) 2.64
Subtotal (I-squared = 14.0%, p = 0.293) 1.03 (0.81, 1.30) 57.28
.
RT
Xu-sheng (2010) 3.28 (0.96, 11.20) 1.29
He (2014) 0.17 (0.07, 0.42) 7.71
Xiao-mei (2014) 1.40 (0.50, 3.87) 2.67
Wei (2014) 0.98 (0.35, 2.73) 3.09
Zhang (2017) 0.83 (0.25, 2.79) 2.42
Yang (2020) 1.59 (1.11, 2.27) 19.94
Subtotal (I-squared = 78.4%, p = 0.000) 1.24 (0.93, 1.64) 37.12
.
Other
Xiujin (2017) 0.34 (0.10, 1.15) 4.04
Wang (2017) 1.06 (0.26, 4.34) 1.56
Subtotal (I-squared = 30.6%, p = 0.230) 0.54 (0.21, 1.35) 5.60
.
Overall (I-squared = 48.8%, p = 0.004) 1.08 (0.90, 1.29) 100.00
.0105
1
94.9

Supplementary File 4

Overall and subgroup analyses for association between XRCC1 rs25487 and side effects. The genetic model includes heterozygote model (GA vs GG), homozygote model (AA vs GG), and dominant model (GA+AA vs GG). The subgroup includes types of acute/late, cancer, cut-off, side effects, and treatment.

GA VS GG overall analysis

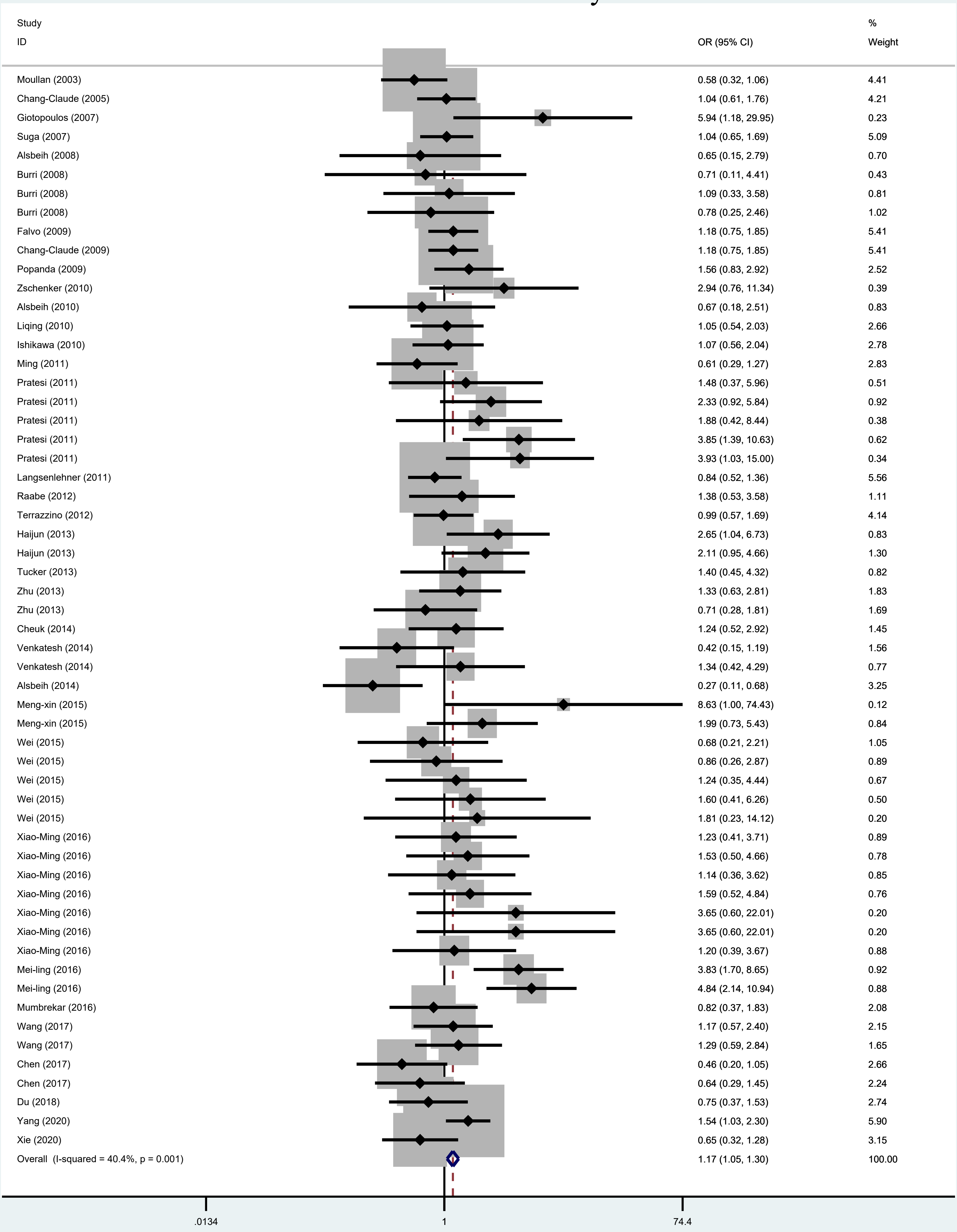

GA VS GG by Acute/Late

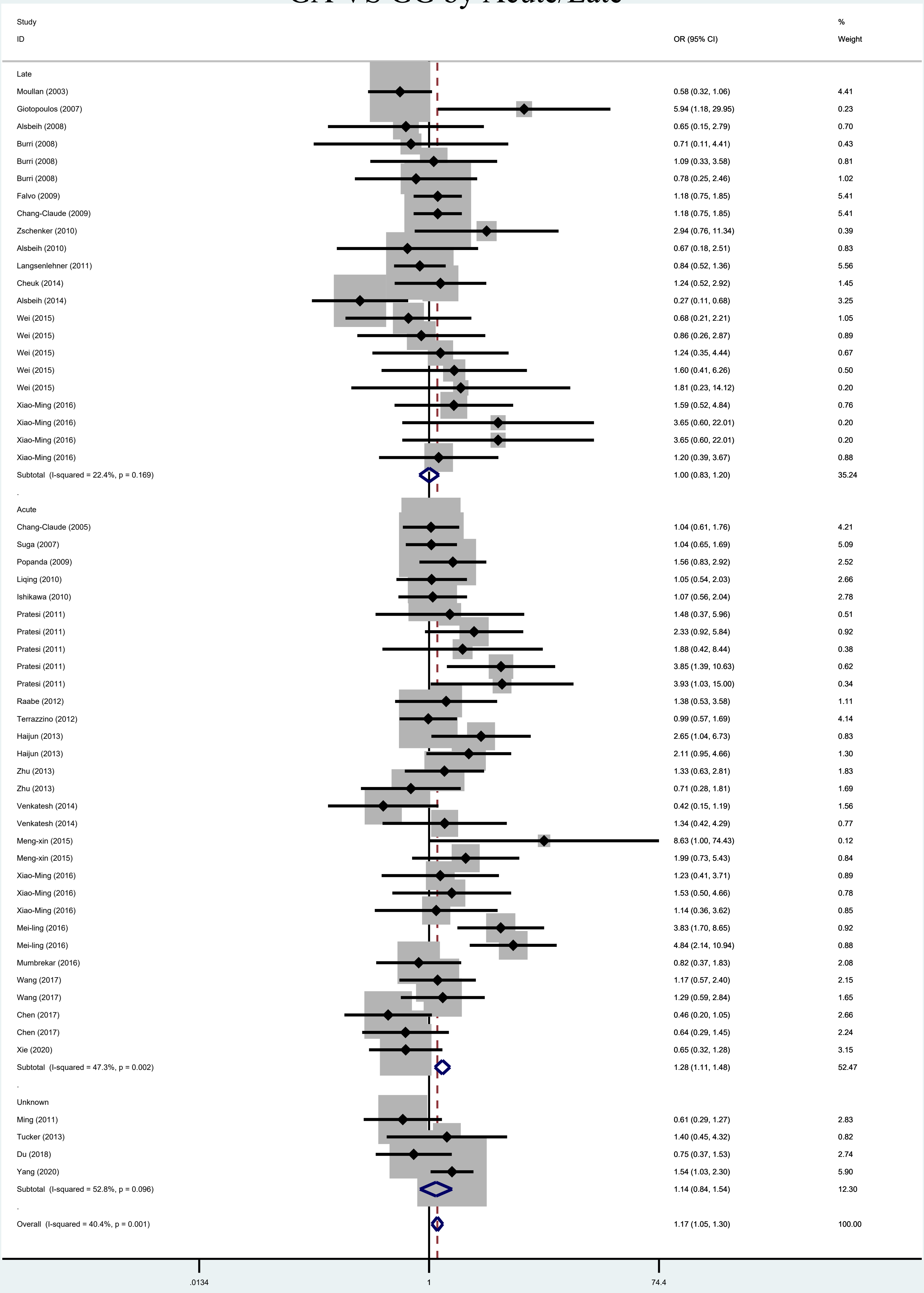

GA VS GG by Cancer

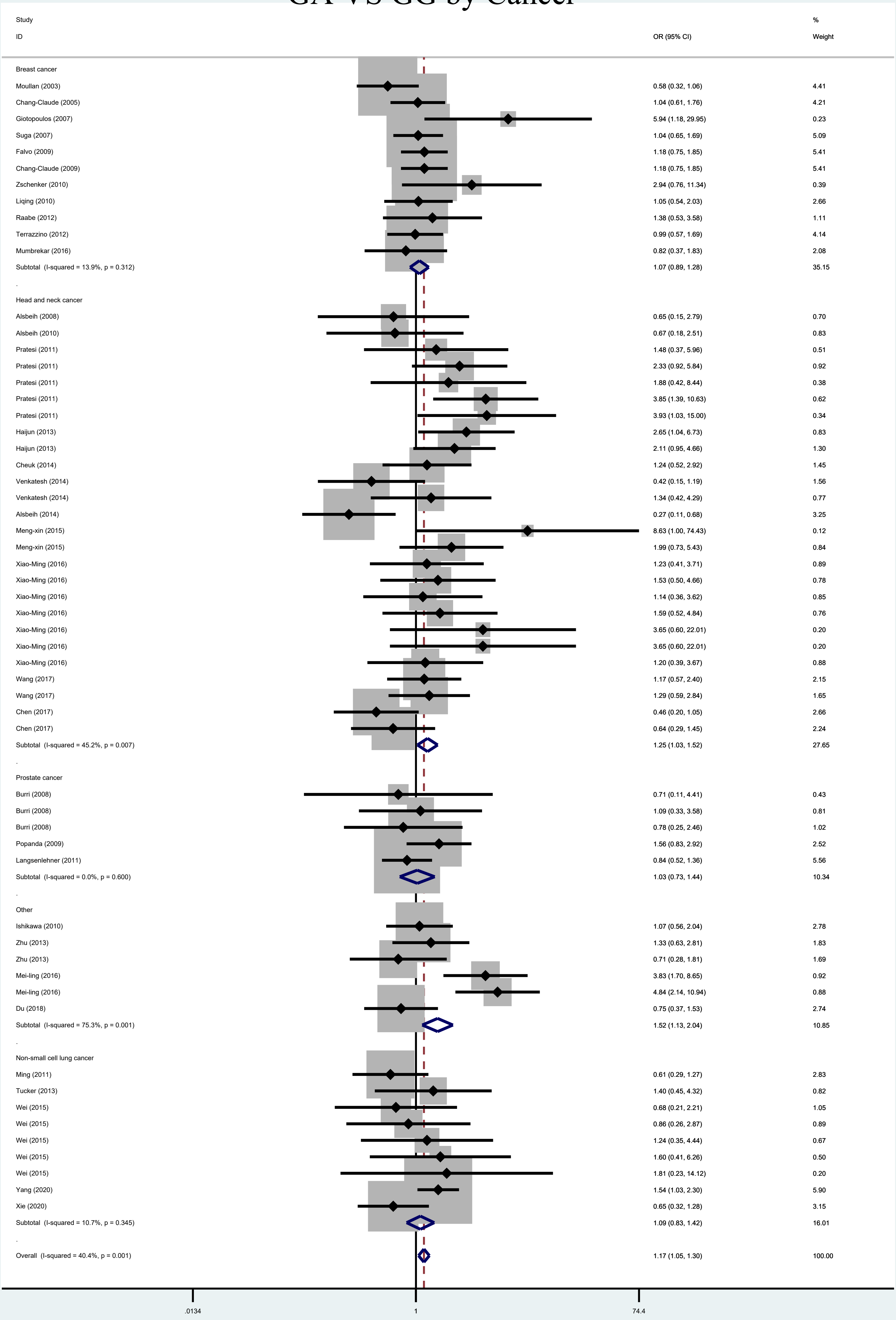

GA VS GG by Cut-off

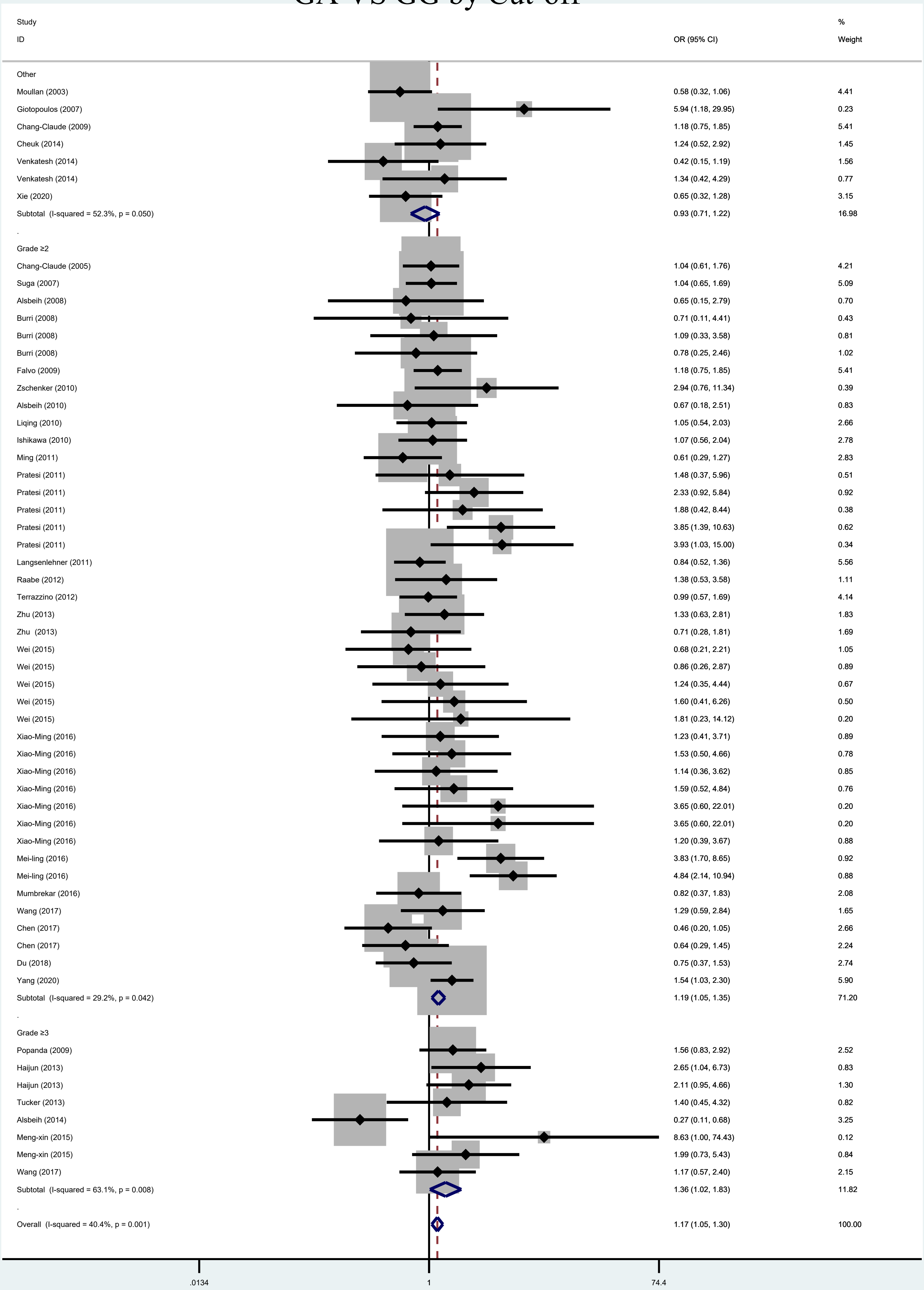

GA VS GG by Side effects

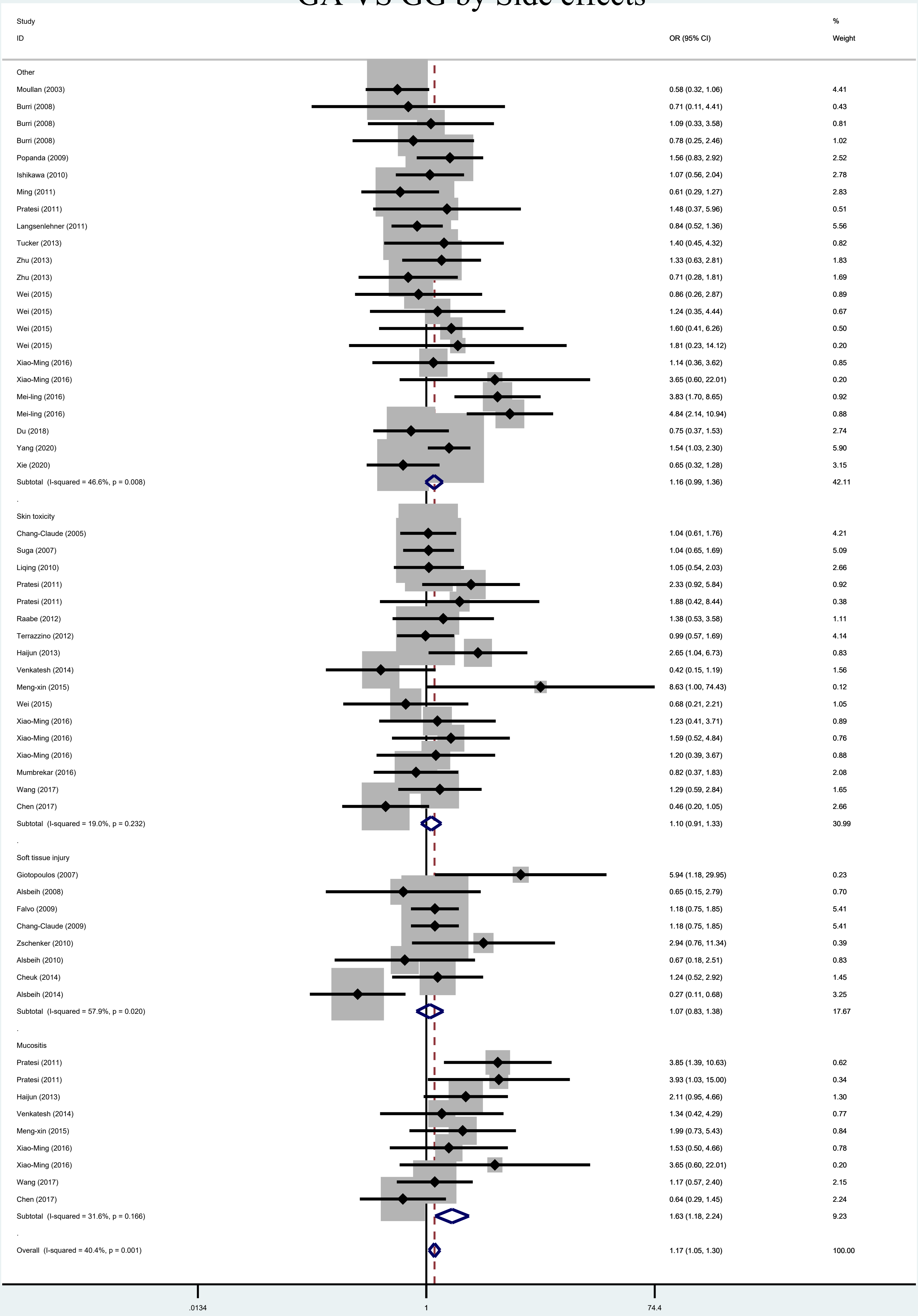

GA VS GG by Treatment

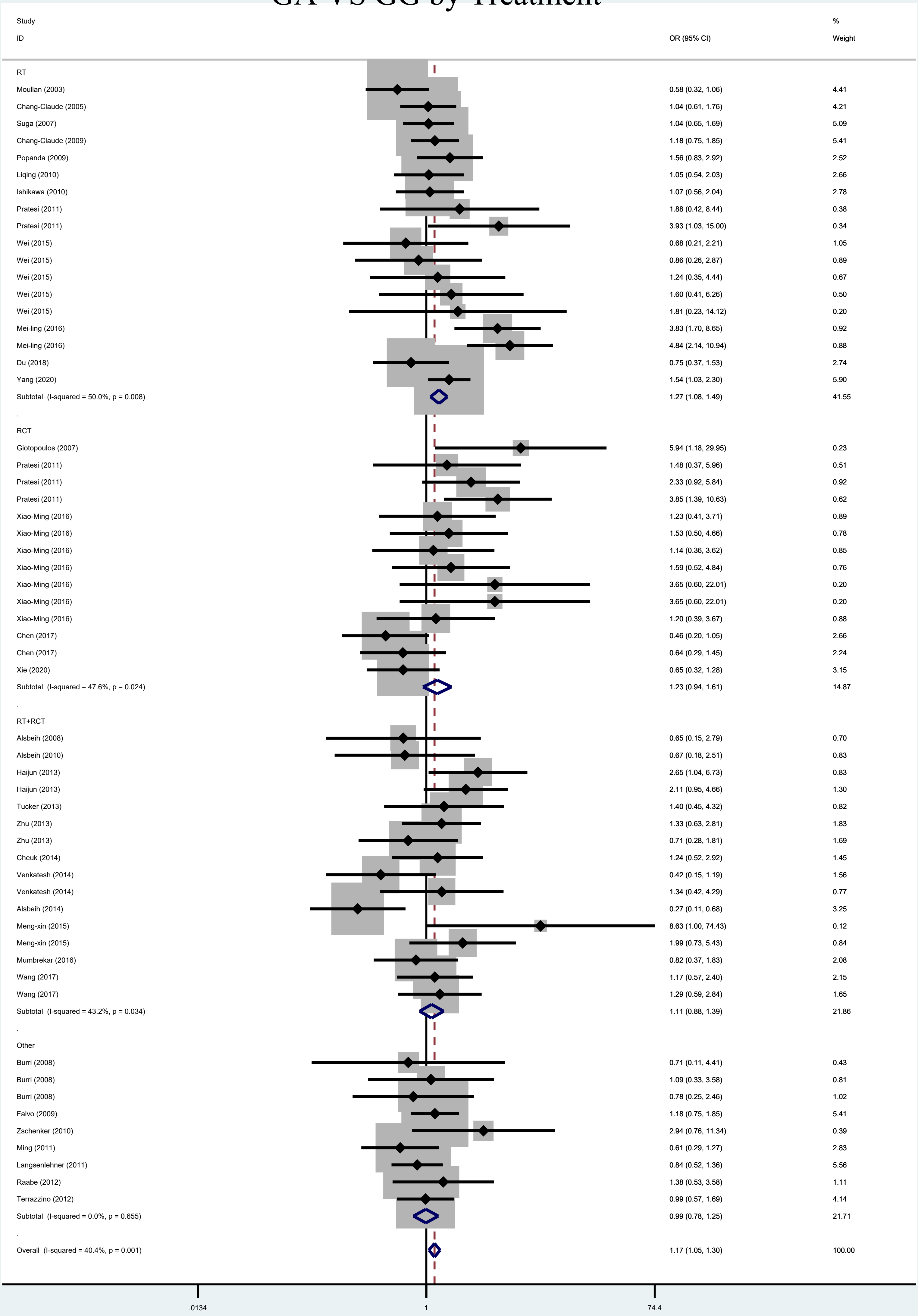

AA VS GG overall analysis

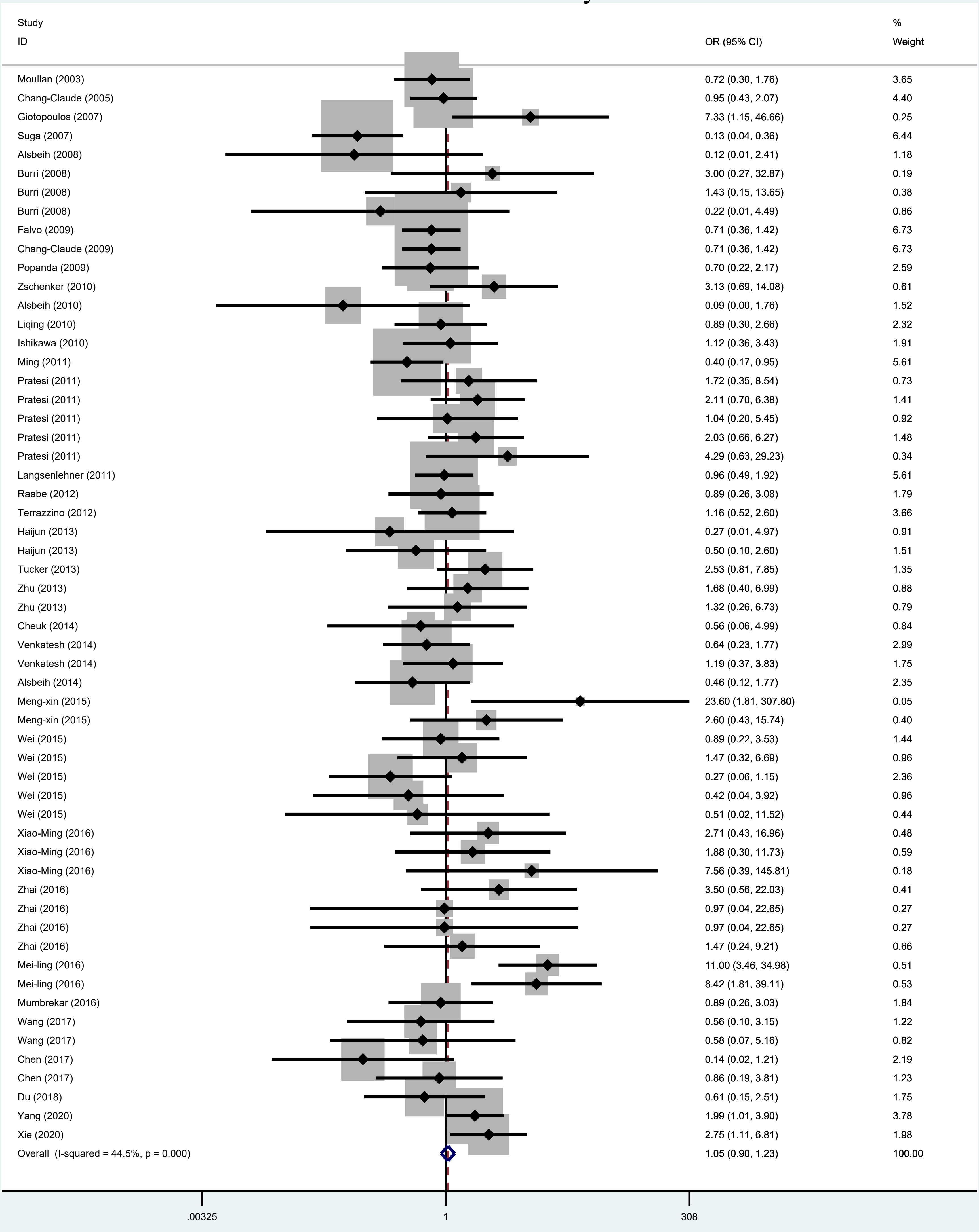

AA VS GG by Acute/Late

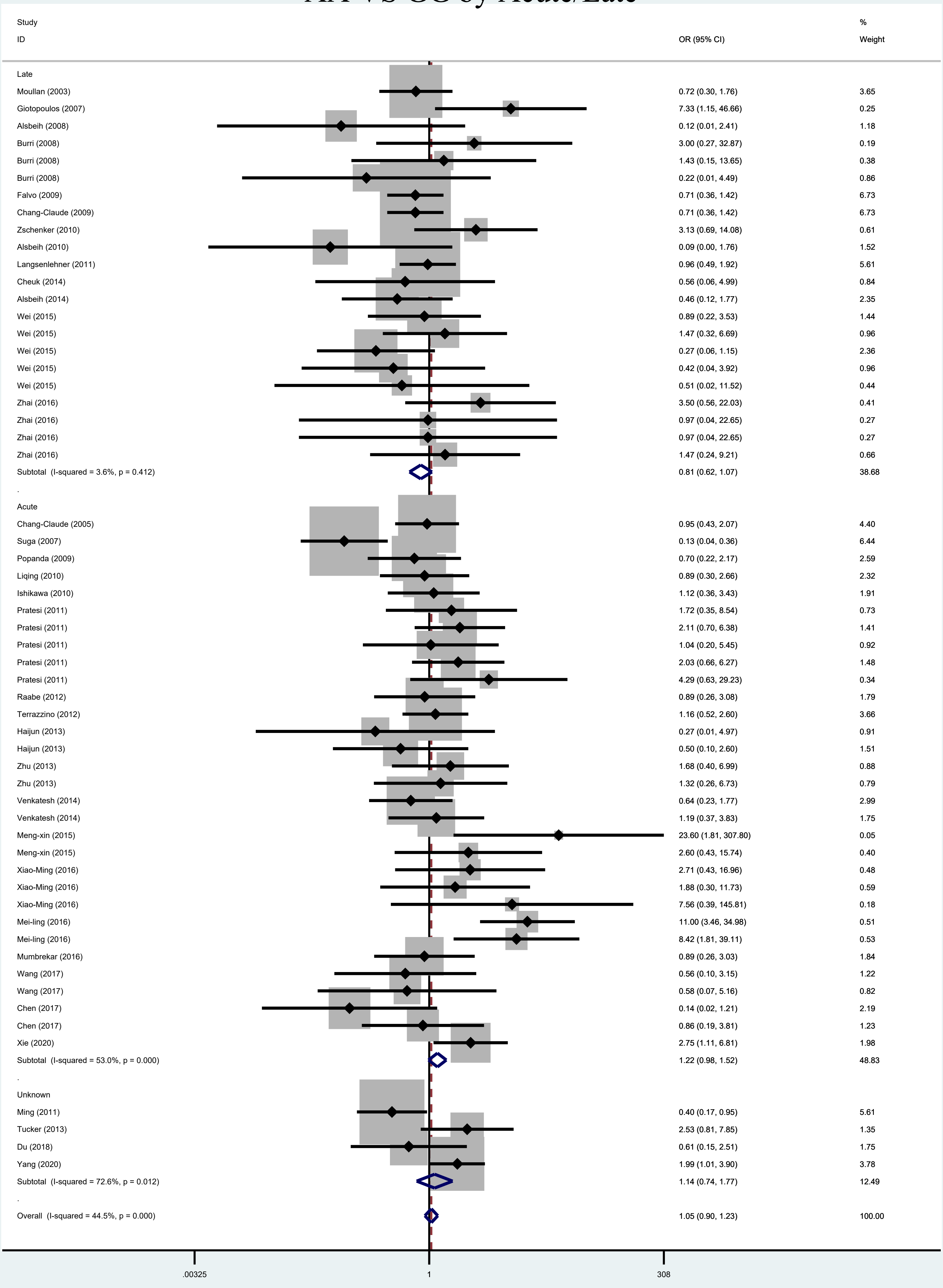

AA VS GG by Cancer

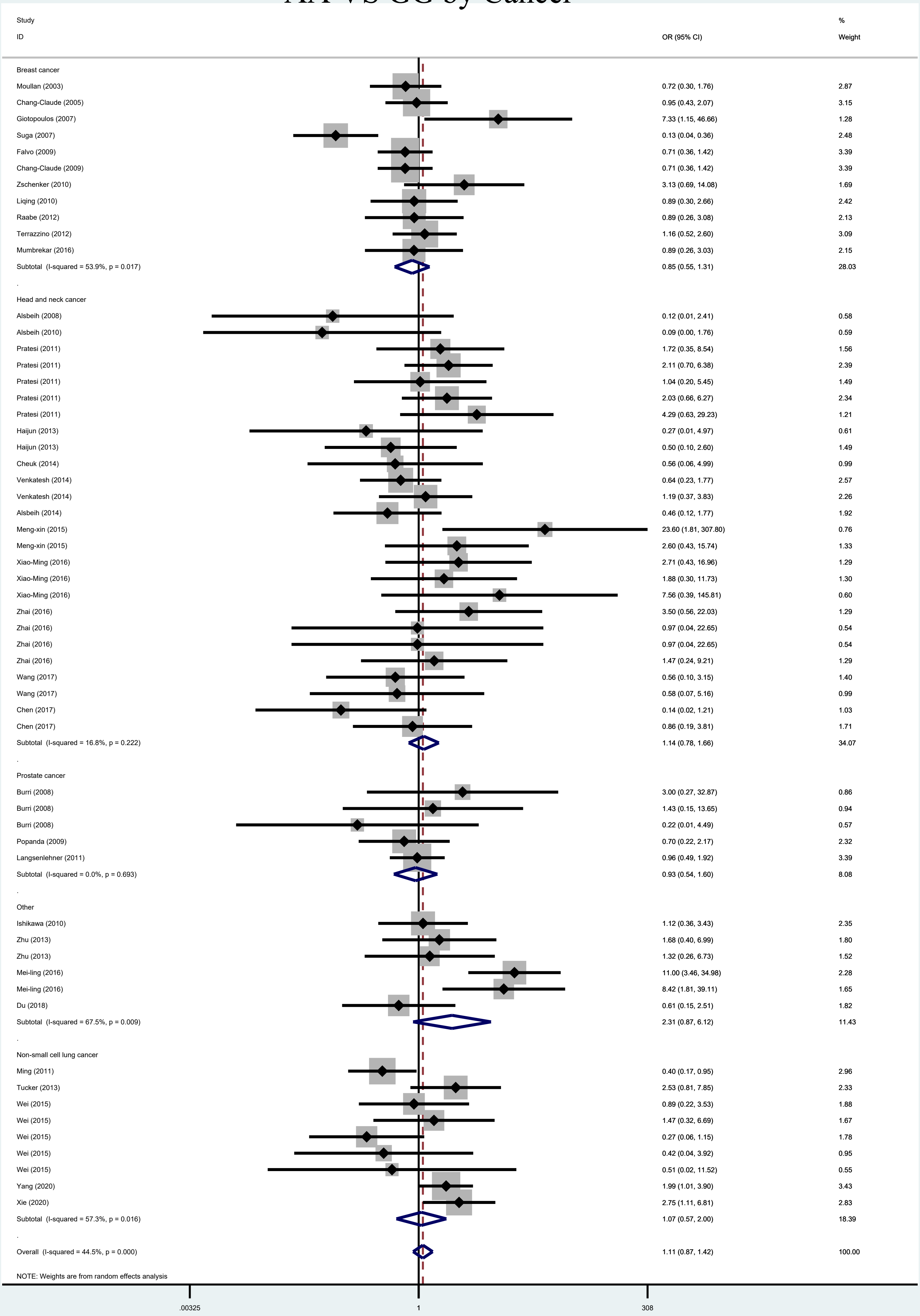

AA VS GG by Cut-off

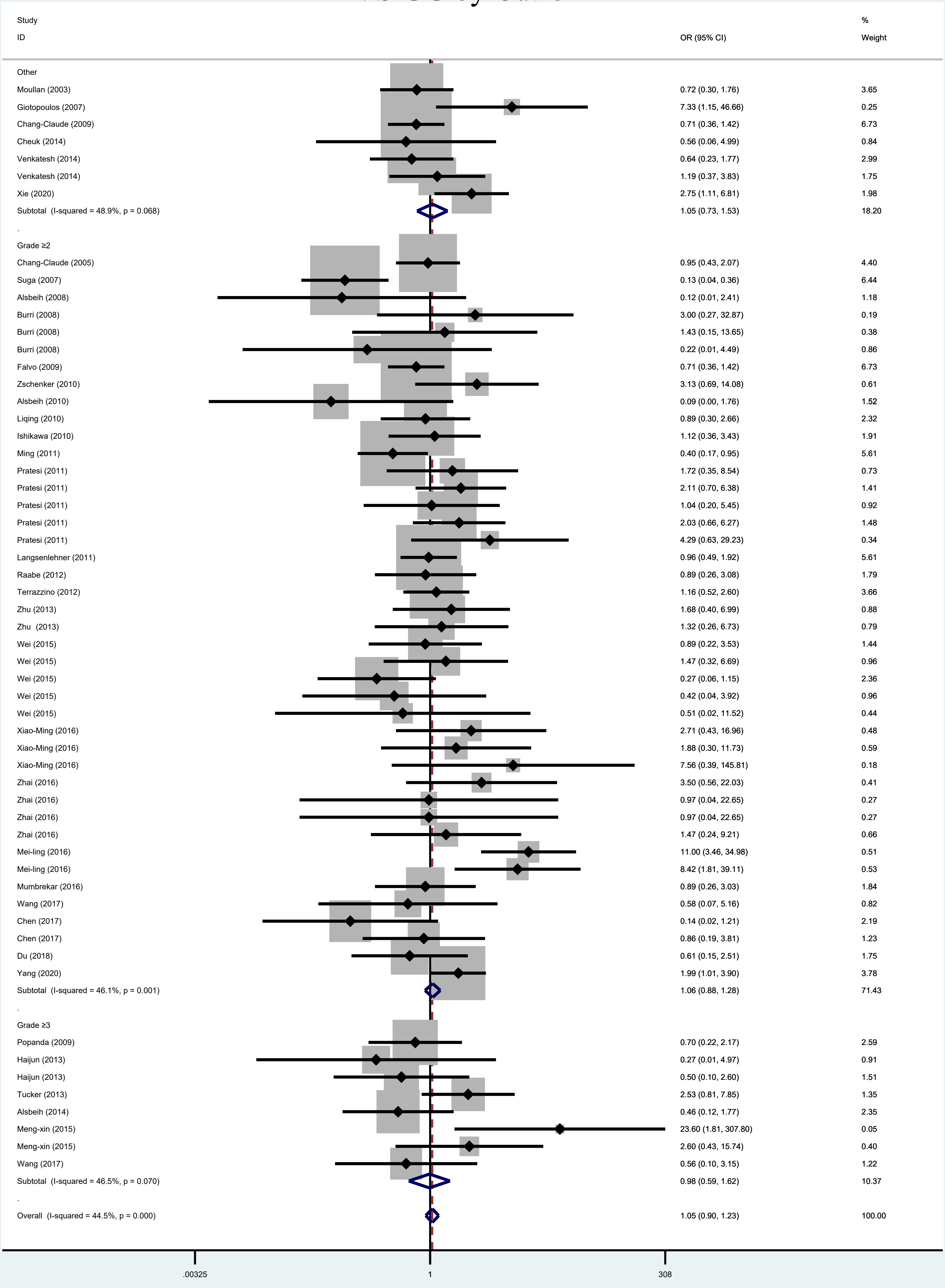

AA VS GG by Side effects

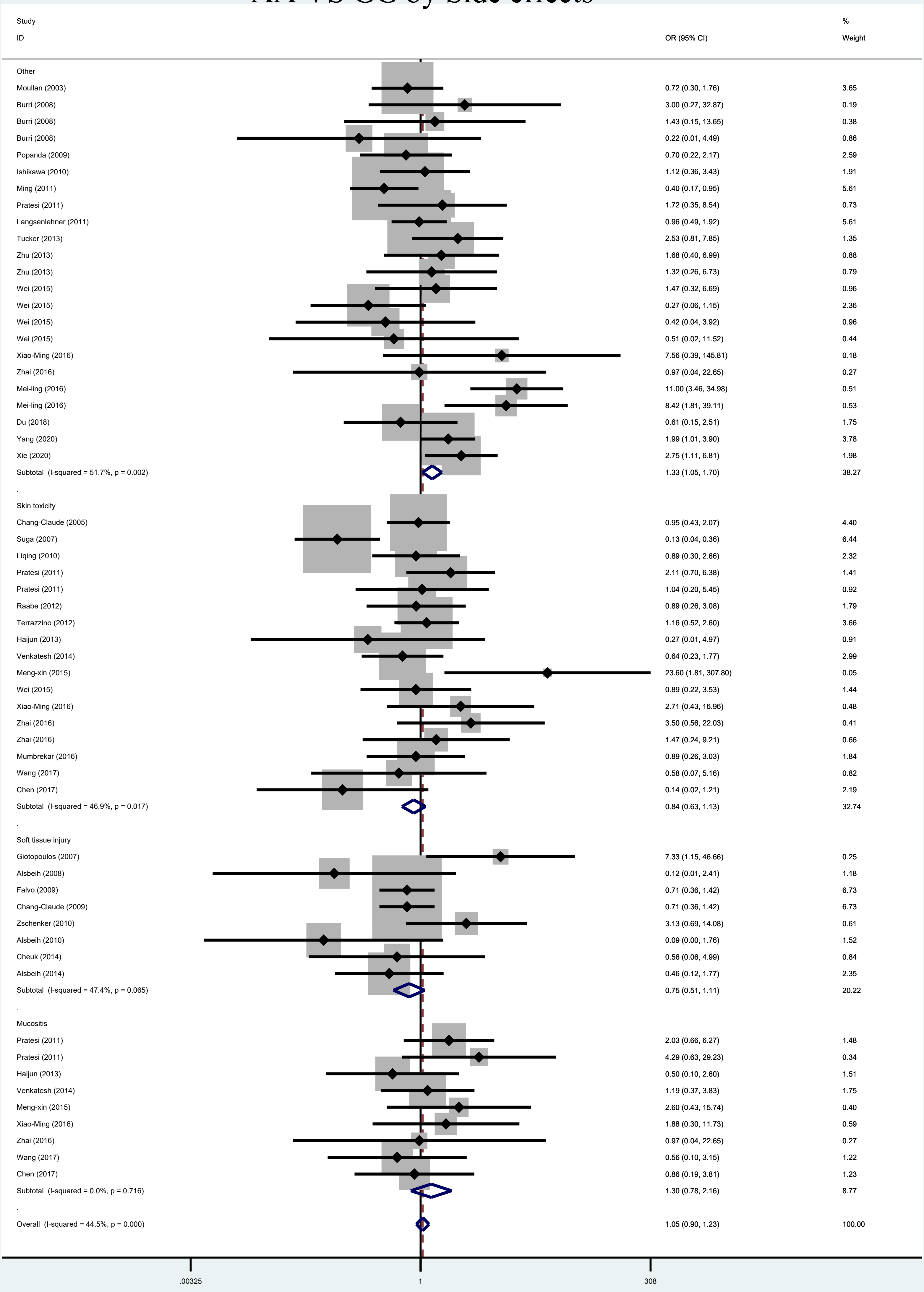

AA VS GG by Treatment

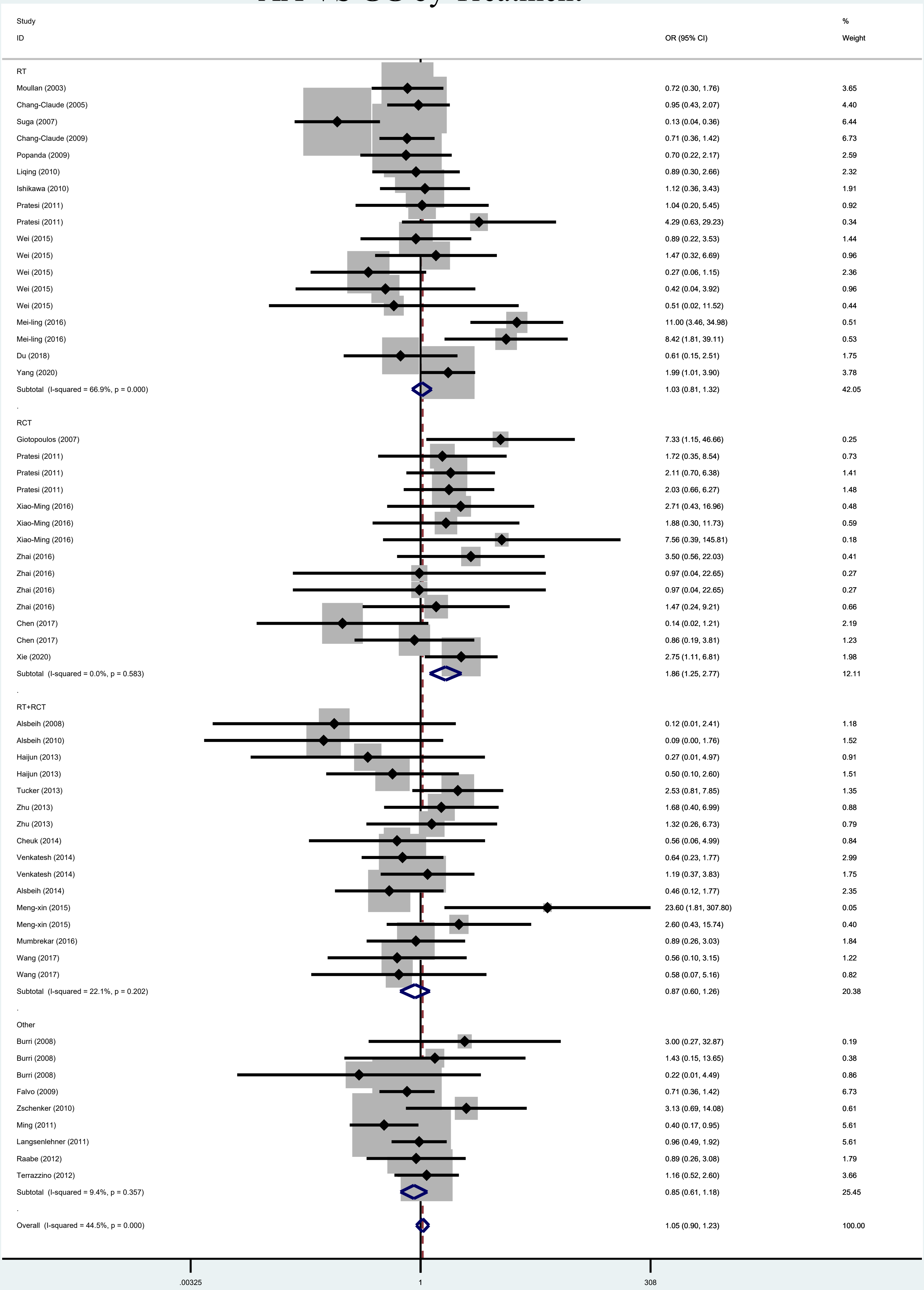

GA+AA VS GG overall analysis

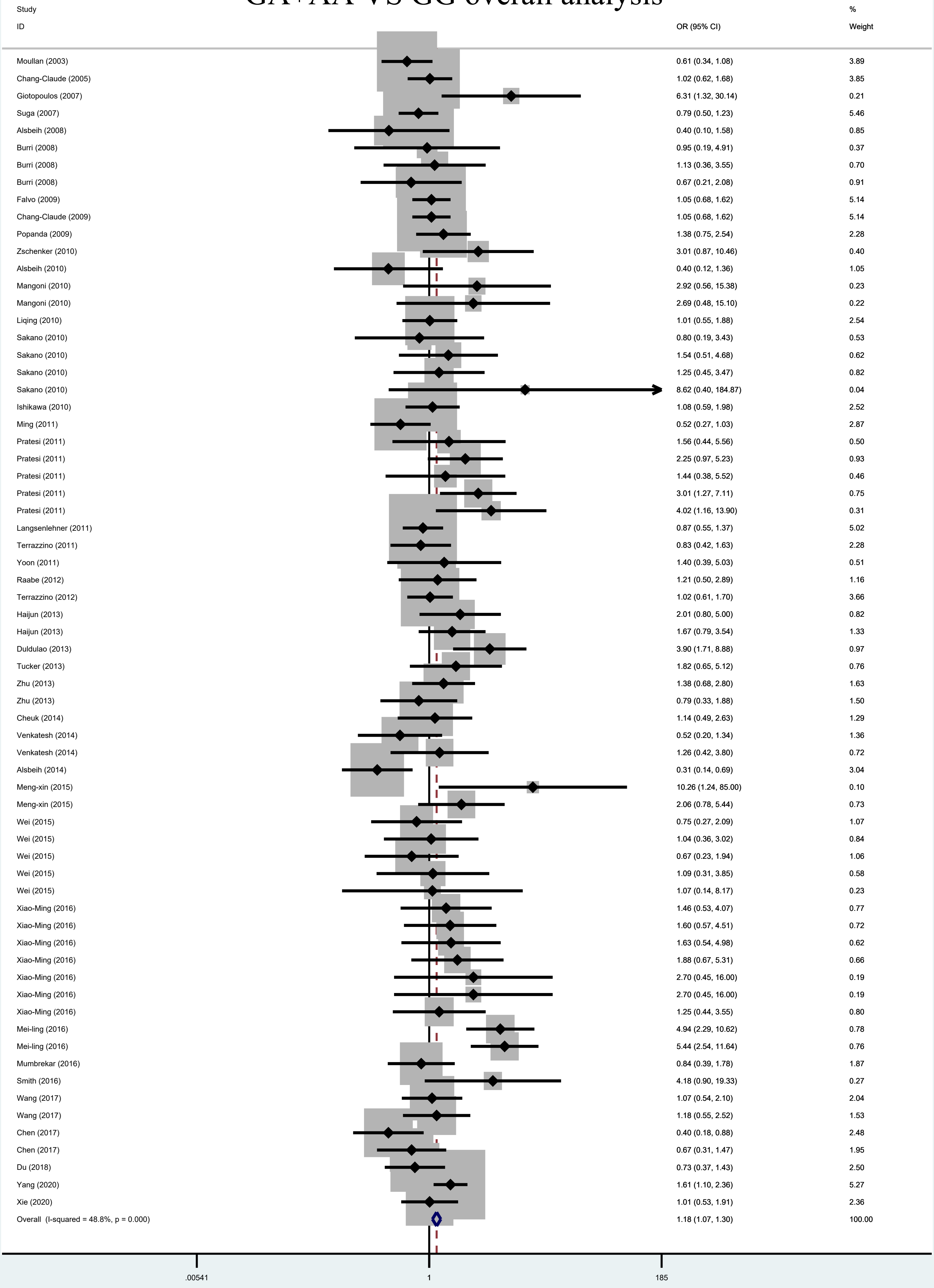

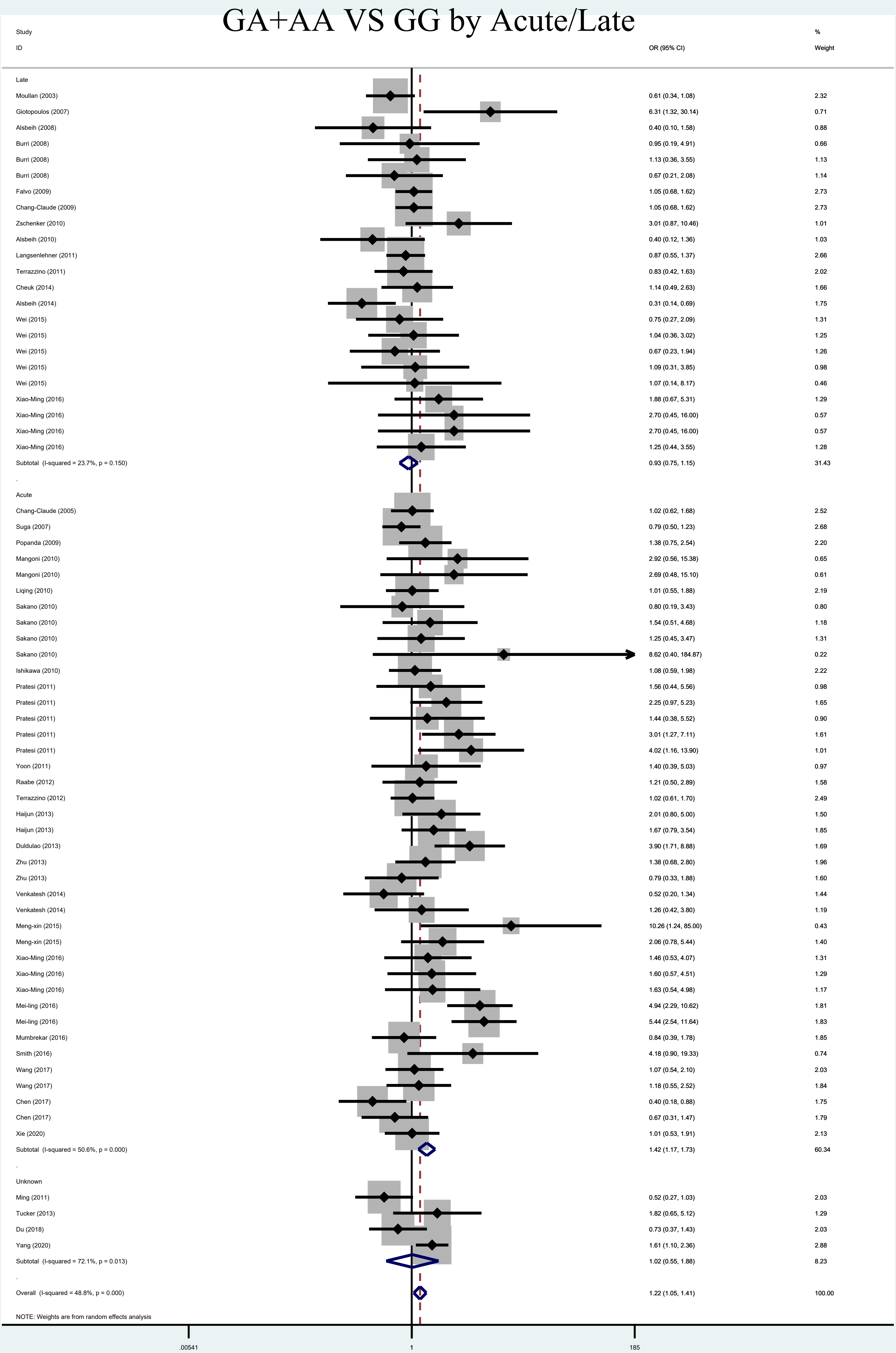
GA+AA VS GG by Acute/Late
Study
ID
OR (95% CI)
%
Weight
Late
Moullan (2003) 0.61 (0.34, 1.08) 2.32
Giotopoulos (2007) 6.31 (1.32, 30.14) 0.71
Alsbeih (2008) 0.40 (0.10, 1.58) 0.88
Burri (2008) 0.95 (0.19, 4.91) 0.66
Burri (2008) 1.13 (0.36, 3.55) 1.13
Burri (2008) 0.67 (0.21, 2.08) 1.14
Falvo (2009) 1.05 (0.68, 1.62) 2.73
Chang-Claude (2009) 1.05 (0.68, 1.62) 2.73
Zschenker (2010) 3.01 (0.87, 10.46) 1.01
Alsbeih (2010) 0.40 (0.12, 1.36) 1.03
Langsenlehner (2011) 0.87 (0.55, 1.37) 2.66
Terrazzino (2011) 0.83 (0.42, 1.63) 2.02
Cheuk (2014) 1.14 (0.49, 2.63) 1.66
Alsbeih (2014) 0.31 (0.14, 0.69) 1.75
Wei (2015) 0.75 (0.27, 2.09) 1.31
Wei (2015) 1.04 (0.36, 3.02) 1.25
Wei (2015) 0.67 (0.23, 1.94) 1.26
Wei (2015) 1.09 (0.31, 3.85) 0.98
Wei (2015) 1.07 (0.14, 8.17) 0.46
Xiao-Ming (2016) 1.88 (0.67, 5.31) 1.29
Xiao-Ming (2016) 2.70 (0.45, 16.00) 0.57
Xiao-Ming (2016) 2.70 (0.45, 16.00) 0.57
Xiao-Ming (2016) 1.25 (0.44, 3.55) 1.28
Subtotal (I-squared = 23.7%, p = 0.150) 0.93 (0.75, 1.15) 31.43
.
Acute
Chang-Claude (2005) 1.02 (0.62, 1.68) 2.52
Suga (2007) 0.79 (0.50, 1.23) 2.68
Popanda (2009) 1.38 (0.75, 2.54) 2.20
Mangoni (2010) 2.92 (0.56, 15.38) 0.65
Mangoni (2010) 2.69 (0.48, 15.10) 0.61
Liqing (2010) 1.01 (0.55, 1.88) 2.19
Sakano (2010) 0.80 (0.19, 3.43) 0.80
Sakano (2010) 1.54 (0.51, 4.68) 1.18
Sakano (2010) 1.25 (0.45, 3.47) 1.31
Sakano (2010) 8.62 (0.40, 184.87) 0.22
Ishikawa (2010) 1.08 (0.59, 1.98) 2.22
Pratesi (2011) 1.56 (0.44, 5.56) 0.98
Pratesi (2011) 2.25 (0.97, 5.23) 1.65
Pratesi (2011) 1.44 (0.38, 5.52) 0.90
Pratesi (2011) 3.01 (1.27, 7.11) 1.61
Pratesi (2011) 4.02 (1.16, 13.90) 1.01
Yoon (2011) 1.40 (0.39, 5.03) 0.97
Raabe (2012) 1.21 (0.50, 2.89) 1.58
Terrazzino (2012) 1.02 (0.61, 1.70) 2.49
Haijun (2013) 2.01 (0.80, 5.00) 1.50
Haijun (2013) 1.67 (0.79, 3.54) 1.85
Duldulao (2013) 3.90 (1.71, 8.88) 1.69
Zhu (2013) 1.38 (0.68, 2.80) 1.96
Zhu (2013) 0.79 (0.33, 1.88) 1.60
Venkatesh (2014) 0.52 (0.20, 1.34) 1.44
Venkatesh (2014) 1.26 (0.42, 3.80) 1.19
Meng-xin (2015) 10.26 (1.24, 85.00) 0.43
Meng-xin (2015) 2.06 (0.78, 5.44) 1.40
Xiao-Ming (2016) 1.46 (0.53, 4.07) 1.31
Xiao-Ming (2016) 1.60 (0.57, 4.51) 1.29
Xiao-Ming (2016) 1.63 (0.54, 4.98) 1.17
Mei-ling (2016) 4.94 (2.29, 10.62) 1.81
Mei-ling (2016) 5.44 (2.54, 11.64) 1.83
Mumbrekar (2016) 0.84 (0.39, 1.78) 1.85
Smith (2016) 4.18 (0.90, 19.33) 0.74
Wang (2017) 1.07 (0.54, 2.10) 2.03
Wang (2017) 1.18 (0.55, 2.52) 1.84
Chen (2017) 0.40 (0.18, 0.88) 1.75
Chen (2017) 0.67 (0.31, 1.47) 1.79
Xie (2020) 1.01 (0.53, 1.91) 2.13
Subtotal (I-squared = 50.6%, p = 0.000) 1.42 (1.17, 1.73) 60.34
.
Unknown
Ming (2011) 0.52 (0.27, 1.03) 2.03
Tucker (2013) 1.82 (0.65, 5.12) 1.29
Du (2018) 0.73 (0.37, 1.43) 2.03
Yang (2020) 1.61 (1.10, 2.36) 2.88
Subtotal (I-squared = 72.1%, p = 0.013) 1.02 (0.55, 1.88) 8.23
.
Overall (I-squared = 48.8%, p = 0.000) 1.22 (1.05, 1.41) 100.00
NOTE: Weights are from random effects analysis
.00541
1
185

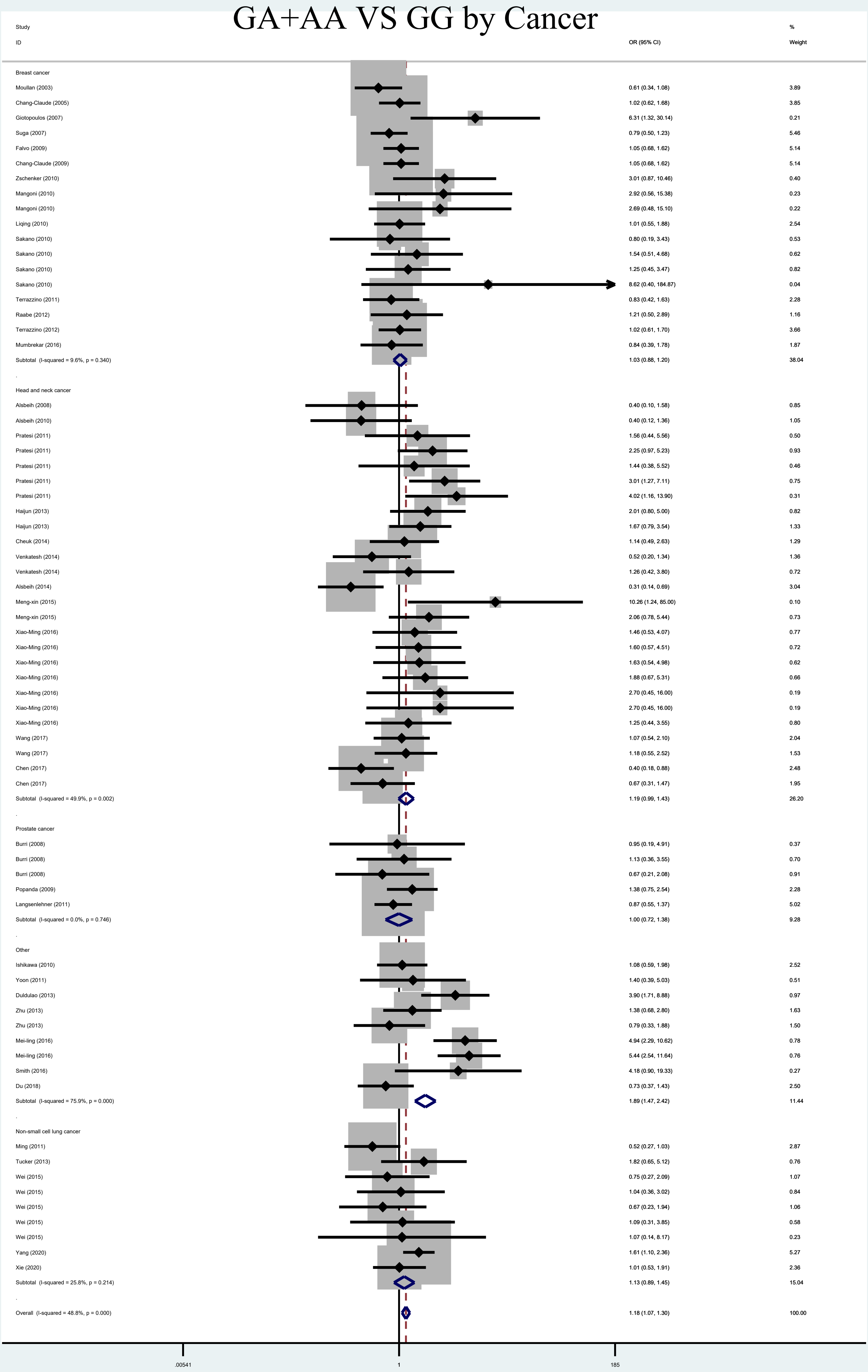
GA+AA VS GG by Cancer
Study ID
OR (95% CI)
% Weight
Breast cancer
Moullan (2003) 0.61 (0.34, 1.08) 3.89
Chang-Claude (2005) 1.02 (0.62, 1.68) 3.85
Giotopoulos (2007) 6.31 (1.32, 30.14) 0.21
Suga (2007) 0.79 (0.50, 1.23) 5.46
Falvo (2009) 1.05 (0.68, 1.62) 5.14
Chang-Claude (2009) 1.05 (0.68, 1.62) 5.14
Zschenker (2010) 3.01 (0.87, 10.46) 0.40
Mangoni (2010) 2.92 (0.56, 15.38) 0.23
Mangoni (2010) 2.69 (0.48, 15.10) 0.22
Liqing (2010) 1.01 (0.55, 1.88) 2.54
Sakano (2010) 0.80 (0.19, 3.43) 0.53
Sakano (2010) 1.54 (0.51, 4.68) 0.62
Sakano (2010) 1.25 (0.45, 3.47) 0.82
Sakano (2010) 8.62 (0.40, 184.87) 0.04
Terrazzino (2011) 0.83 (0.42, 1.63) 2.28
Raabe (2012) 1.21 (0.50, 2.89) 1.16
Terrazzino (2012) 1.02 (0.61, 1.70) 3.66
Mumbrekar (2016) 0.84 (0.39, 1.78) 1.87
Subtotal (I-squared = 9.6%, p = 0.340) 1.03 (0.88, 1.20) 38.04
.
Head and neck cancer
Alsbeih (2008) 0.40 (0.10, 1.58) 0.85
Alsbeih (2010) 0.40 (0.12, 1.36) 1.05
Pratesi (2011) 1.56 (0.44, 5.56) 0.50
Pratesi (2011) 2.25 (0.97, 5.23) 0.93
Pratesi (2011) 1.44 (0.38, 5.52) 0.46
Pratesi (2011) 3.01 (1.27, 7.11) 0.75
Pratesi (2011) 4.02 (1.16, 13.90) 0.31
Haijun (2013) 2.01 (0.80, 5.00) 0.82
Haijun (2013) 1.67 (0.79, 3.54) 1.33
Cheuk (2014) 1.14 (0.49, 2.63) 1.29
Venkatesh (2014) 0.52 (0.20, 1.34) 1.36
Venkatesh (2014) 1.26 (0.42, 3.80) 0.72
Alsbeih (2014) 0.31 (0.14, 0.69) 3.04
Meng-xin (2015) 10.26 (1.24, 85.00) 0.10
Meng-xin (2015) 2.06 (0.78, 5.44) 0.73
Xiao-Ming (2016) 1.46 (0.53, 4.07) 0.77
Xiao-Ming (2016) 1.60 (0.57, 4.51) 0.72
Xiao-Ming (2016) 1.63 (0.54, 4.98) 0.62
Xiao-Ming (2016) 1.88 (0.67, 5.31) 0.66
Xiao-Ming (2016) 2.70 (0.45, 16.00) 0.19
Xiao-Ming (2016) 2.70 (0.45, 16.00) 0.19
Xiao-Ming (2016) 1.25 (0.44, 3.55) 0.80
Wang (2017) 1.07 (0.54, 2.10) 2.04
Wang (2017) 1.18 (0.55, 2.52) 1.53
Chen (2017) 0.40 (0.18, 0.88) 2.48
Chen (2017) 0.67 (0.31, 1.47) 1.95
Subtotal (I-squared = 49.9%, p = 0.002) 1.19 (0.99, 1.43) 26.20
.
Prostate cancer
Burri (2008) 0.95 (0.19, 4.91) 0.37
Burri (2008) 1.13 (0.36, 3.55) 0.70
Burri (2008) 0.67 (0.21, 2.08) 0.91
Popanda (2009) 1.38 (0.75, 2.54) 2.28
Langsenlehner (2011) 0.87 (0.55, 1.37) 5.02
Subtotal (I-squared = 0.0%, p = 0.746) 1.00 (0.72, 1.38) 9.28
.
Other
Ishikawa (2010) 1.08 (0.59, 1.98) 2.52
Yoon (2011) 1.40 (0.39, 5.03) 0.51
Duldulao (2013) 3.90 (1.71, 8.88) 0.97
Zhu (2013) 1.38 (0.68, 2.80) 1.63
Zhu (2013) 0.79 (0.33, 1.88) 1.50
Mei-ling (2016) 4.94 (2.29, 10.62) 0.78
Mei-ling (2016) 5.44 (2.54, 11.64) 0.76
Smith (2016) 4.18 (0.90, 19.33) 0.27
Du (2018) 0.73 (0.37, 1.43) 2.50
Subtotal (I-squared = 75.9%, p = 0.000) 1.89 (1.47, 2.42) 11.44
.
Non-small cell lung cancer
Ming (2011) 0.52 (0.27, 1.03) 2.87
Tucker (2013) 1.82 (0.65, 5.12) 0.76
Wei (2015) 0.75 (0.27, 2.09) 1.07
Wei (2015) 1.04 (0.36, 3.02) 0.84
Wei (2015) 0.67 (0.23, 1.94) 1.06
Wei (2015) 1.09 (0.31, 3.85) 0.58
Wei (2015) 1.07 (0.14, 8.17) 0.23
Yang (2020) 1.61 (1.10, 2.36) 5.27
Xie (2020) 1.01 (0.53, 1.91) 2.36
Subtotal (I-squared = 25.8%, p = 0.214) 1.13 (0.89, 1.45) 15.04
.
Overall (I-squared = 48.8%, p = 0.000) 1.18 (1.07, 1.30) 100.00
.00541
1
185

GA+AA VS GG by Cut-off

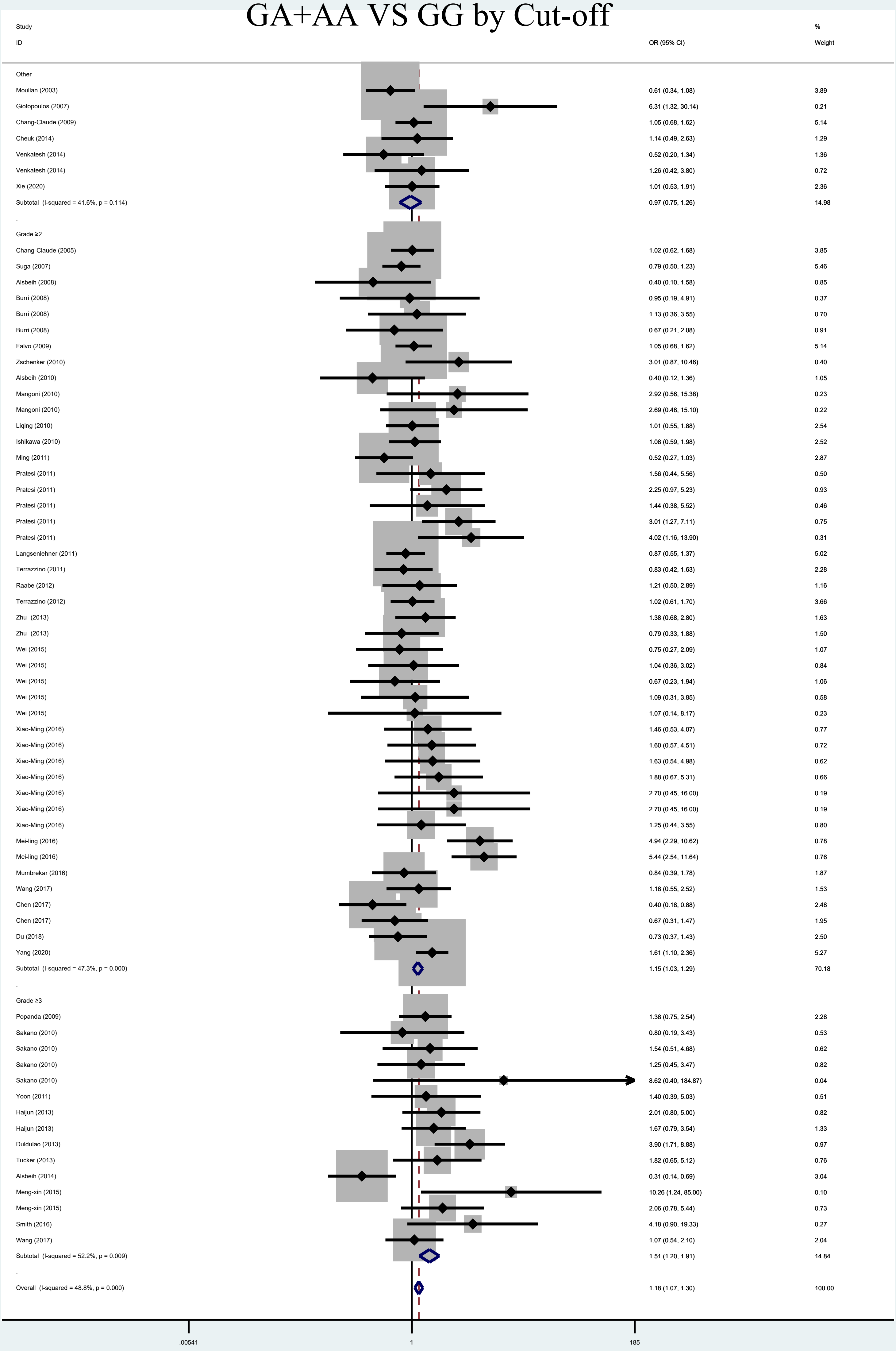

GA+AA VS GG by Side effects

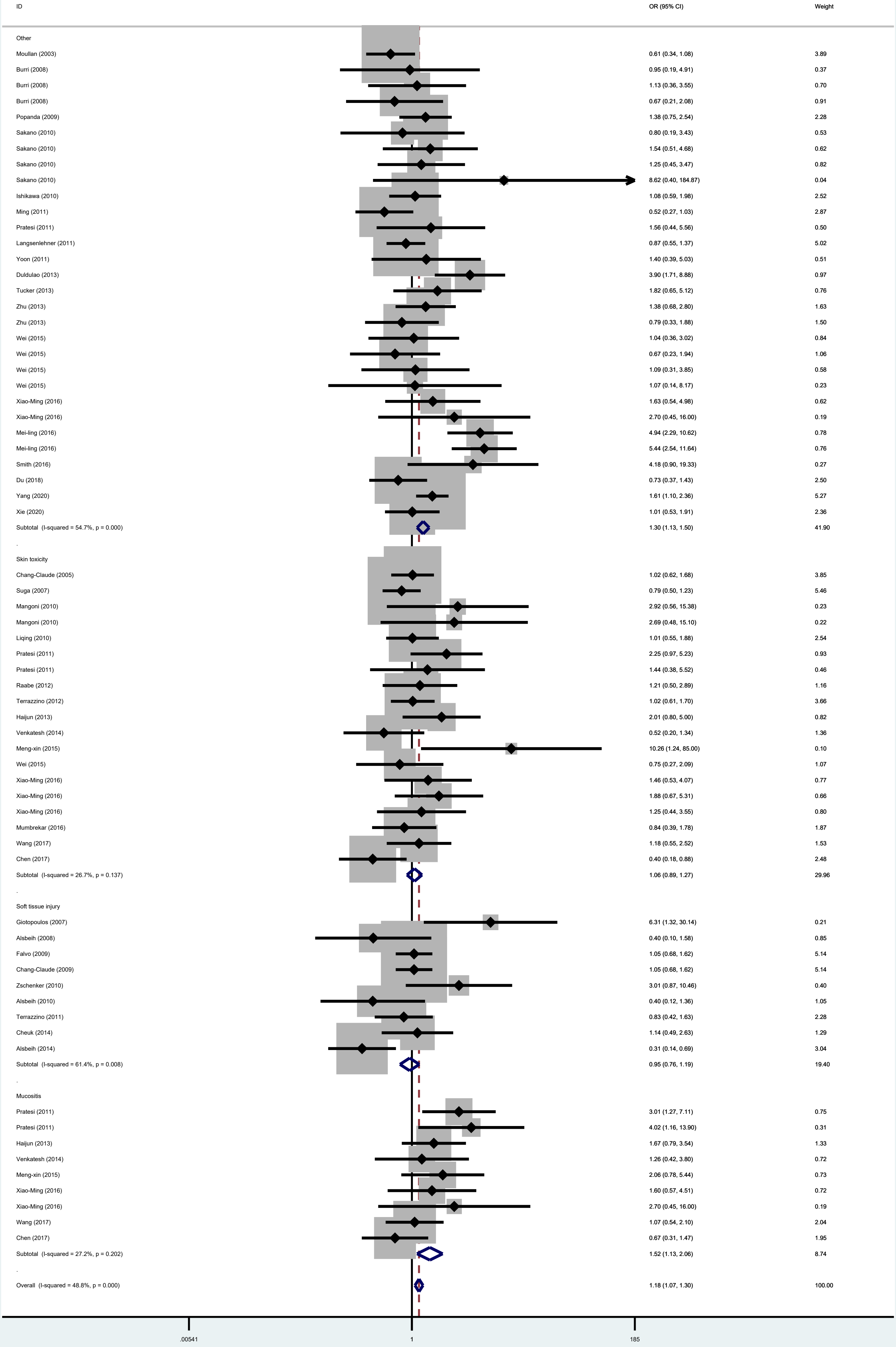

GA+AA VS GG by Treatment

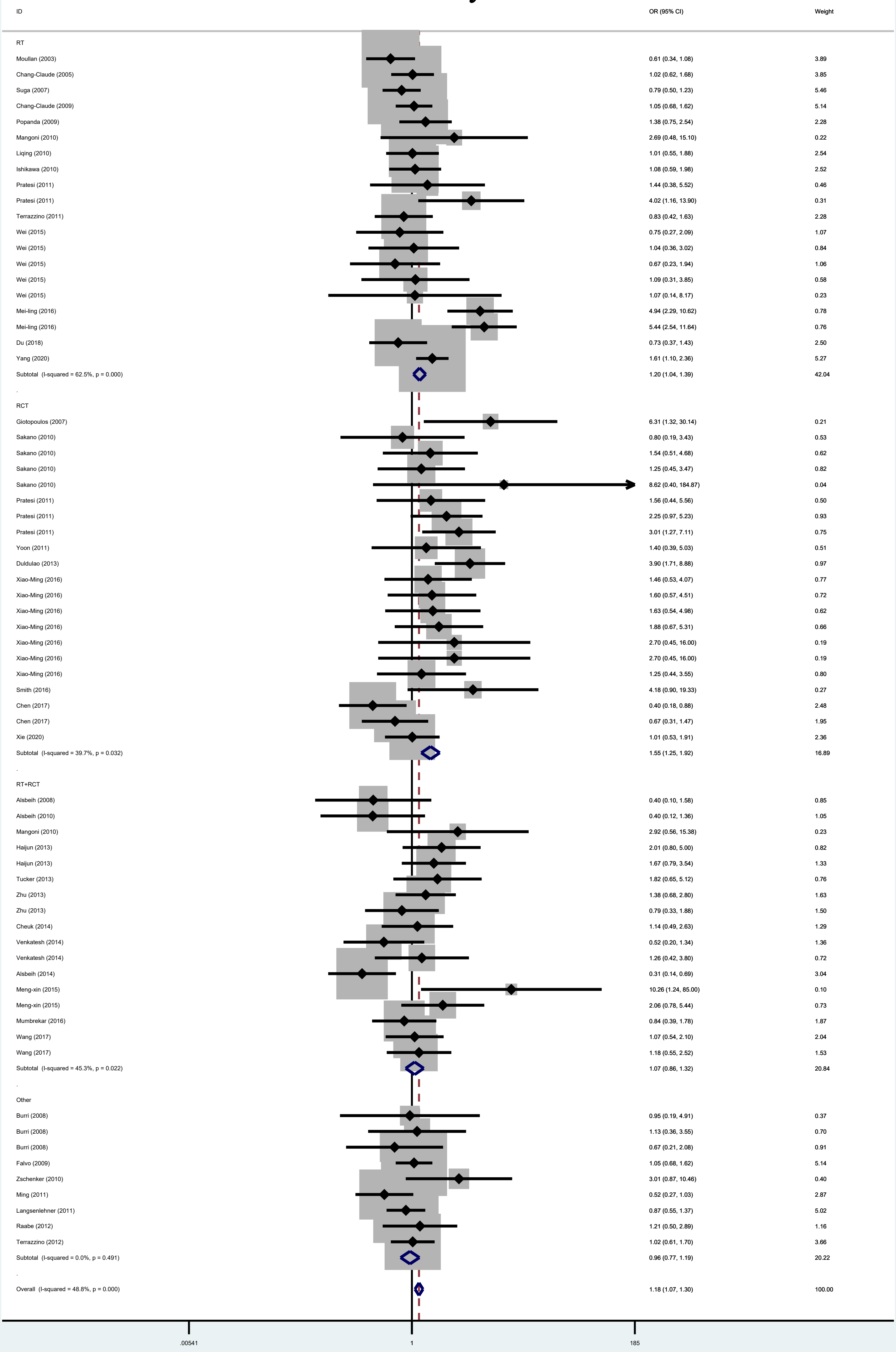

Supplementary File 5

Sensitivity analysis for association between XRCC1 expression and treatment response/ overall survival. The analysis indicate the results are stable.

Sensitivity analysis for association between XRCC1 expression and treatment response

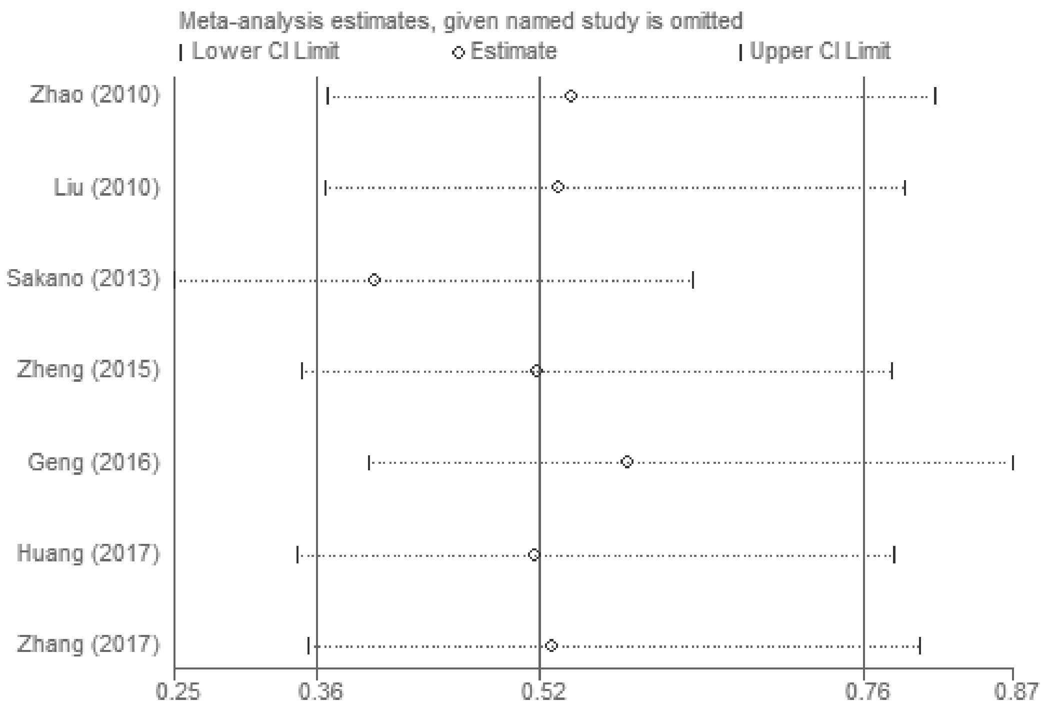

Sensitivity analyses for association between XRCC1 expression and overall survival

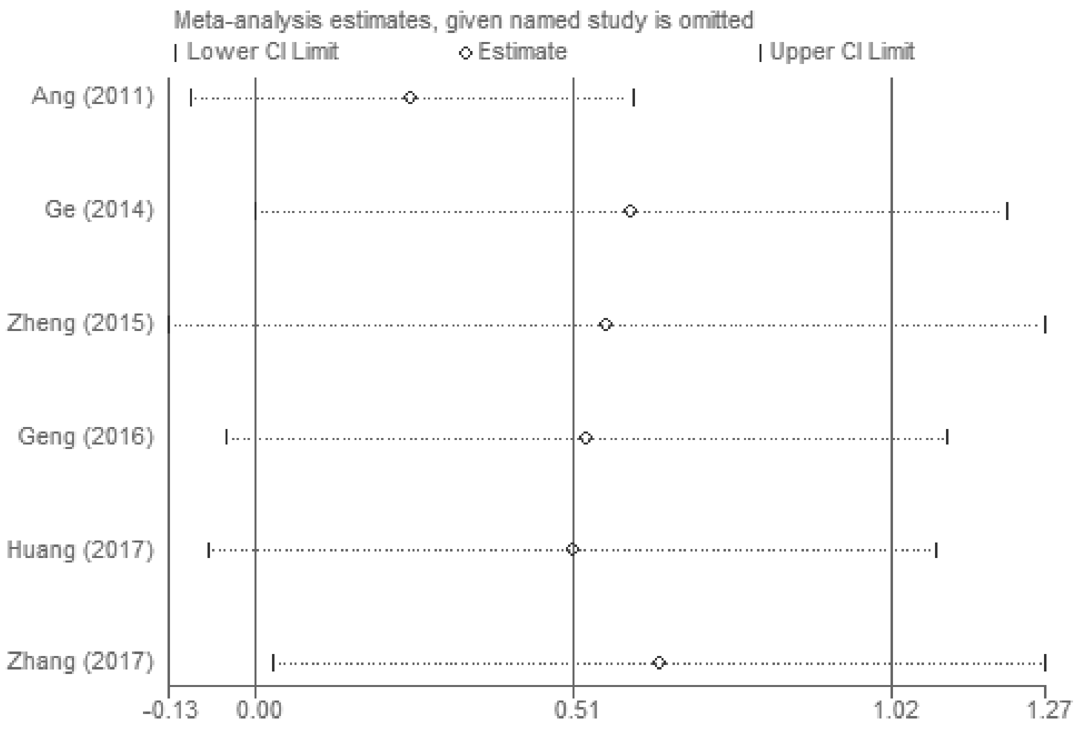

Supplementary File 6

Sensitivity analysis for association between XRCC1 rs25487 and treatment response.

The analysis indicate the results are stable.

AA VS GG

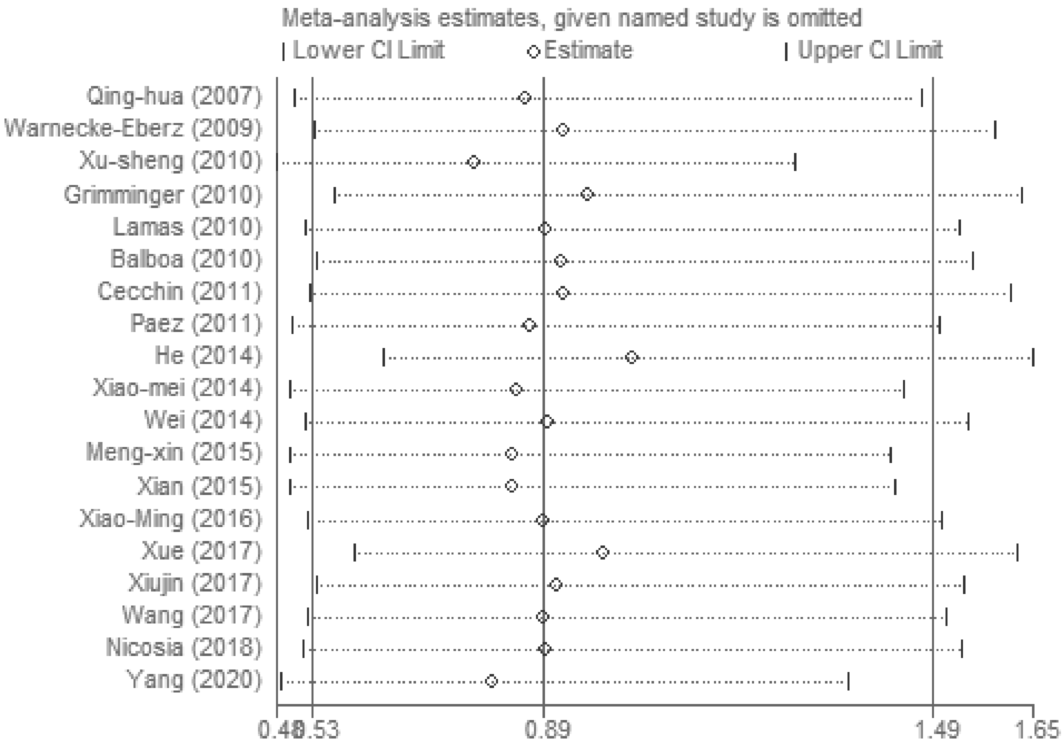

Meta-analysis estimates, given named study is omitted
Lower CI Limit
Estimate
Upper CI Limit
Qing-hua (2007)
Warnecke-Eberz (2009)
Xu-sheng (2010)
Grimminger (2010)
Lamas (2010)
Balboa (2010)
Cecchin (2011)
Paez (2011)
He (2014)
Xiao-mei (2014)
Wei (2014)
Meng-xin (2015)
Xian (2015)
Xiao-Ming (2016)
Xue (2017)
Xiujin (2017)
Wang (2017)
Nicosia (2018)
Yang (2020)
0.53
0.89
1.49
1.65

GA VS GG

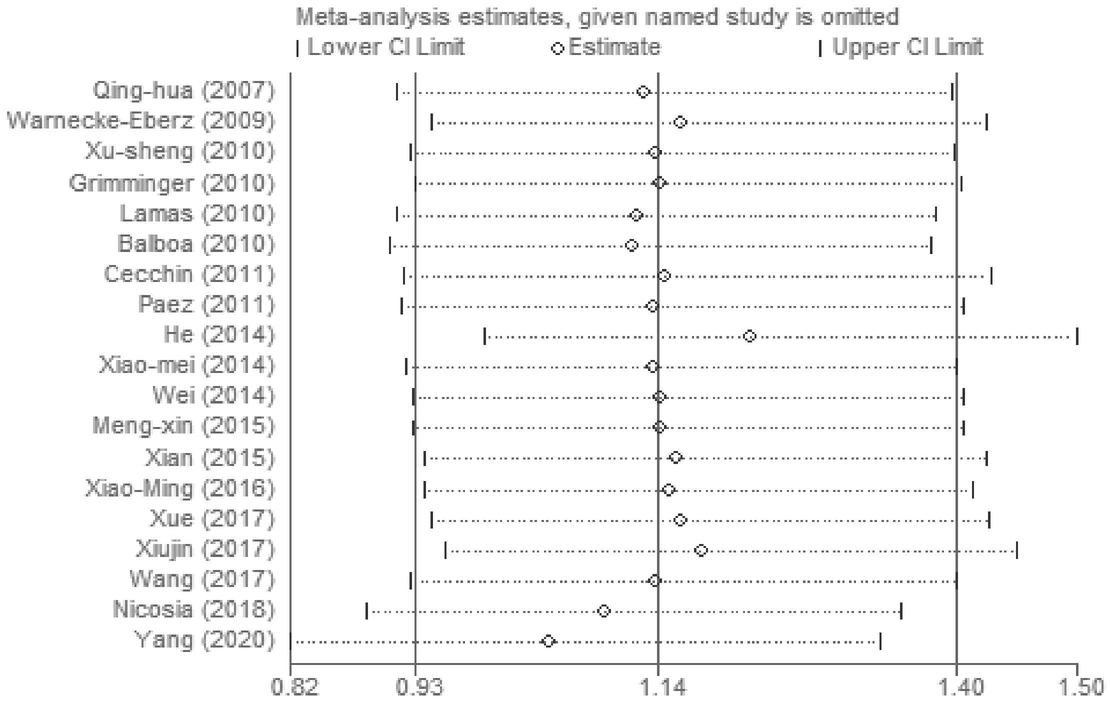
Meta-analysis estimates, given named study is omitted
Lower CI Limit
Estimate
Upper CI Limit
Qing-hua (2007)
Warnecke-Eberz (2009)
Xu-sheng (2010)
Grimminger (2010)
Lamas (2010)
Balboa (2010)
Cecchin (2011)
Paez (2011)
He (2014)
Xiao-mei (2014)
Wei (2014)
Meng-xin (2015)
Xian (2015)
Xiao-Ming (2016)
Xue (2017)
Xiujin (2017)
Wang (2017)
Nicosia (2018)
Yang (2020)
0.82
0.93
1.14
1.40
1.50

GA+AA VS GG

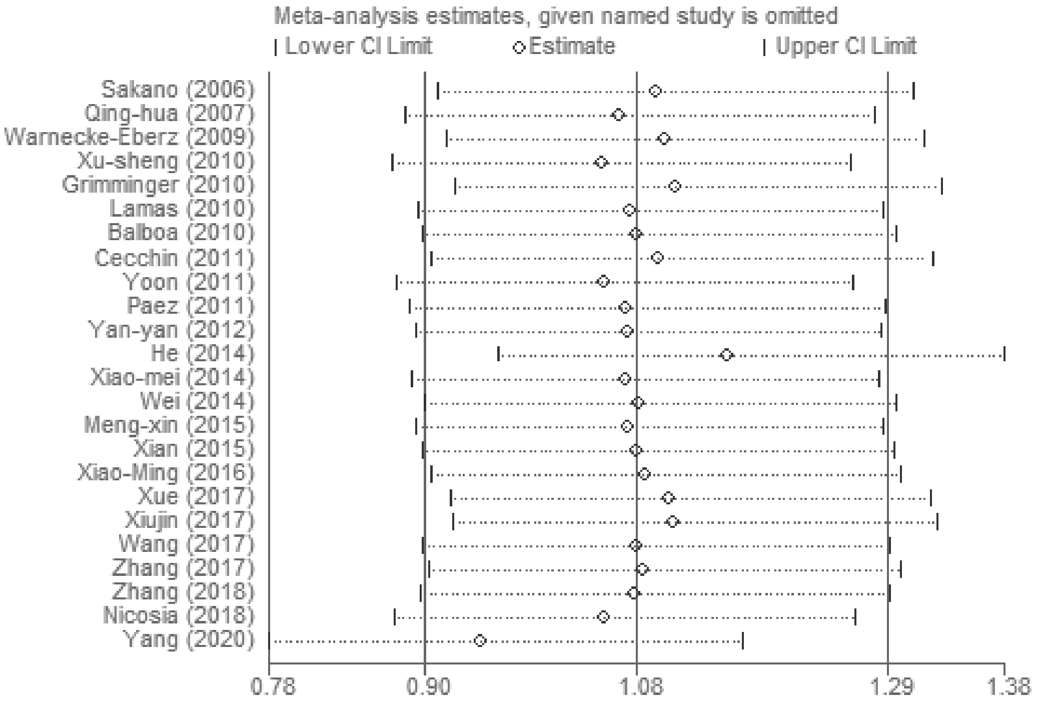

Meta-analysis estimates, given named study is omitted
Lower CI Limit
Estimate
Upper CI Limit
Sakano (2006)
Qing-hua (2007)
Warnecke-Eberz (2009)
Xu-sheng (2010)
Grimminger (2010)
Lamas (2010)
Balboa (2010)
Cecchin (2011)
Yoon (2011)
Paez (2011)
Yan-yan (2012)
He (2014)
Xiao-mei (2014)
Wei (2014)
Meng-xin (2015)
Xian (2015)
Xiao-Ming (2016)
Xue (2017)
Xiujin (2017)
Wang (2017)
Zhang (2017)
Zhang (2018)
Nicosia (2018)
Yang (2020)
0.78
0.90
1.08
1.29
1.38

Supplementary File 7

Sensitivity analysis for association between XRCC1 rs25487 and side effects.

The analysis indicate the results are stable.

AA VS GG

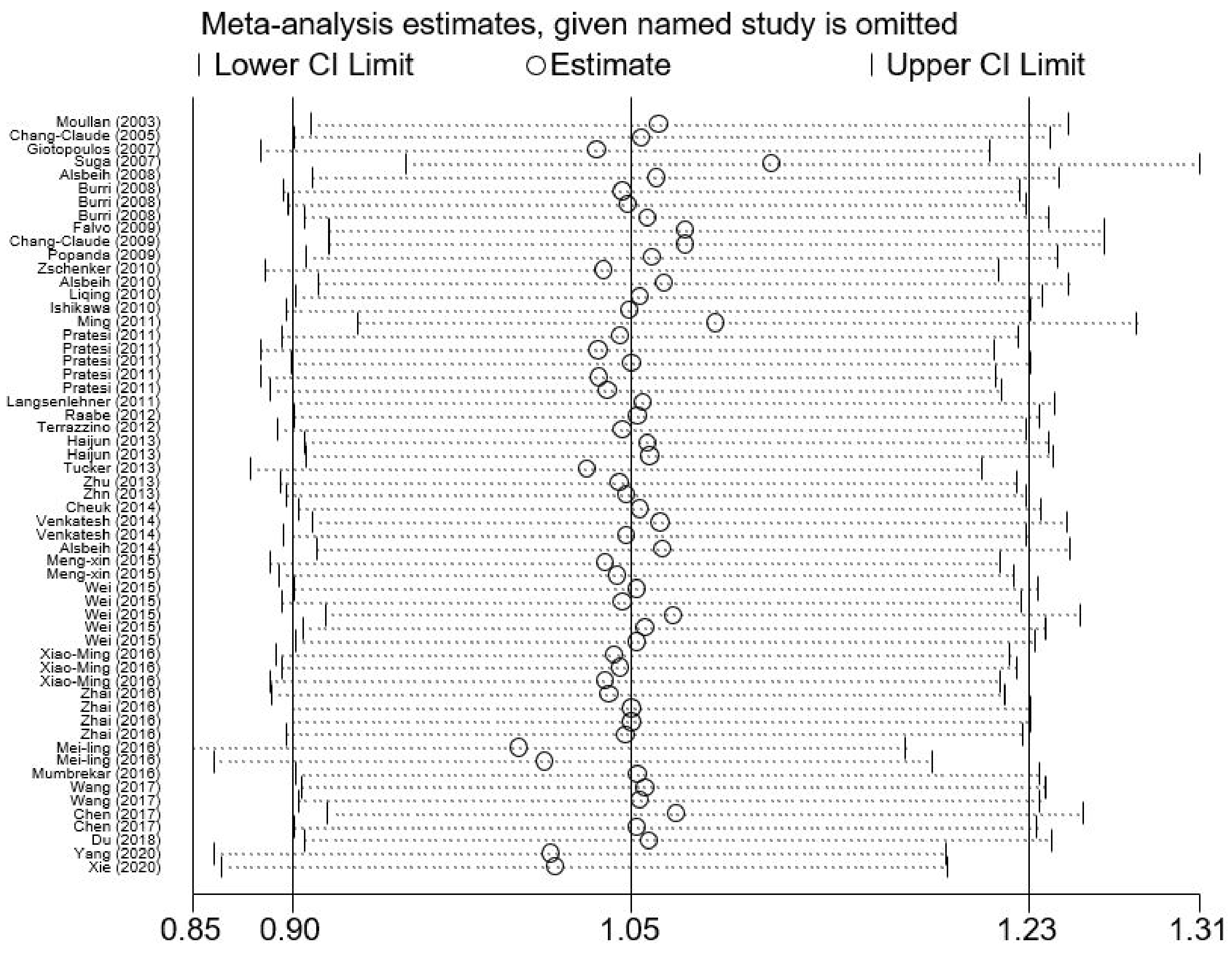

Meta-analysis estimates, given named study is omitted
Lower CI Limit
Estimate
Upper CI Limit
Moullan (2003)
Chang-Claude (2005)
Giotopoulos (2007)
Suga (2007)
Alsbeih (2008)
Burri (2008)
Burri (2008)
Burri (2008)
Falvo (2009)
Chang-Claude (2009)
Popanda (2009)
Zschenker (2010)
Alsbeih (2010)
Liqing (2010)
Ishikawa (2010)
Ming (2011)
Pratesi (2011)
Pratesi (2011)
Pratesi (2011)
Pratesi (2011)
Pratesi (2011)
Langsenlehner (2011)
Raabe (2012)
Terrazzino (2012)
Haijun (2013)
Haijun (2013)
Tucker (2013)
Zhu (2013)
Zhn (2013)
Cheuk (2014)
Venkatesh (2014)
Venkatesh (2014)
Alsbeih (2014)
Meng-xin (2015)
Meng-xin (2015)
Wei (2015)
Wei (2015)
Wei (2015)
Wei (2015)
Wei (2015)
Xiao-Ming (2016)
Xiao-Ming (2016)
Xiao-Ming (2016)
Zhai (2016)
Zhai (2016)
Zhai (2016)
Zhai (2016)
Mei-ling (2016)
Mei-ling (2016)
Mumbrekar (2016)
Wang (2017)
Wang (2017)
Chen (2017)
Chen (2017)
Du (2018)
Yang (2020)
Xie (2020)
0.85
0.90
1.05
1.23
1.31

GA VS GG

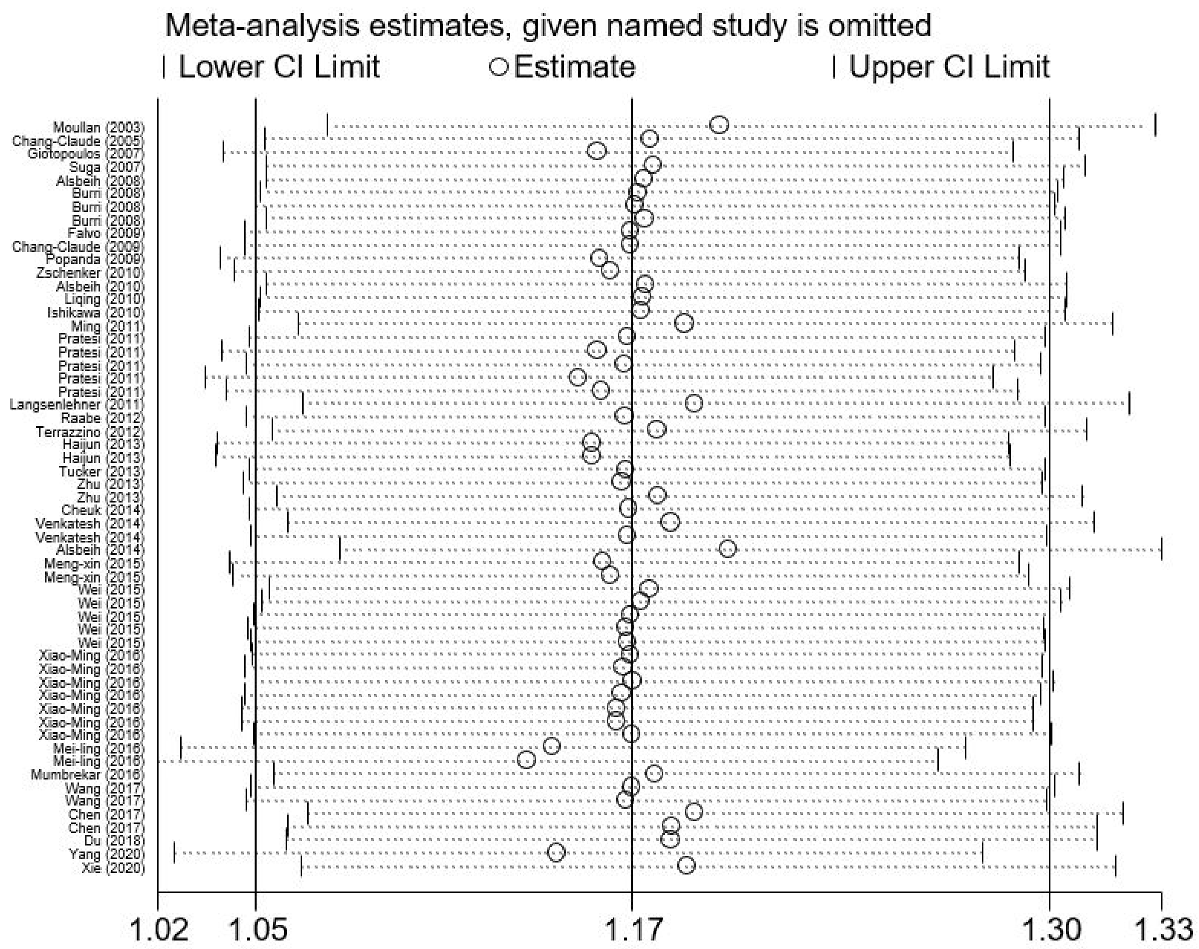

Meta-analysis estimates, given named study is omitted
Lower CI Limit
Estimate
Upper CI Limit
Moullan (2003)
Chang-Claude (2005)
Giotopoulos (2007)
Suga (2007)
Alsbeih (2008)
Burri (2008)
Burri (2008)
Burri (2008)
Falvo (2009)
Chang-Claude (2009)
Popanda (2009)
Zschenker (2010)
Alsbeih (2010)
Liqing (2010)
Ishikawa (2010)
Ming (2011)
Pratesi (2011)
Pratesi (2011)
Pratesi (2011)
Pratesi (2011)
Pratesi (2011)
Langsenlehner (2011)
Raabe (2012)
Terrazzino (2012)
Haijun (2013)
Haijun (2013)
Tucker (2013)
Zhu (2013)
Zhu (2013)
Cheuk (2014)
Venkatesh (2014)
Venkatesh (2014)
Alsbeih (2014)
Meng-xin (2015)
Meng-xin (2015)
Wei (2015)
Wei (2015)
Wei (2015)
Wei (2015)
Wei (2015)
Xiao-Ming (2016)
Xiao-Ming (2016)
Xiao-Ming (2016)
Xiao-Ming (2016)
Xiao-Ming (2016)
Xiao-Ming (2016)
Xiao-Ming (2016)
Mei-ling (2016)
Mei-ling (2016)
Mumbrekar (2016)
Wang (2017)
Wang (2017)
Chen (2017)
Chen (2017)
Du (2018)
Yang (2020)
Xie (2020)
1.02
1.05
1.17
1.30
1.33

GA+AA VS GG

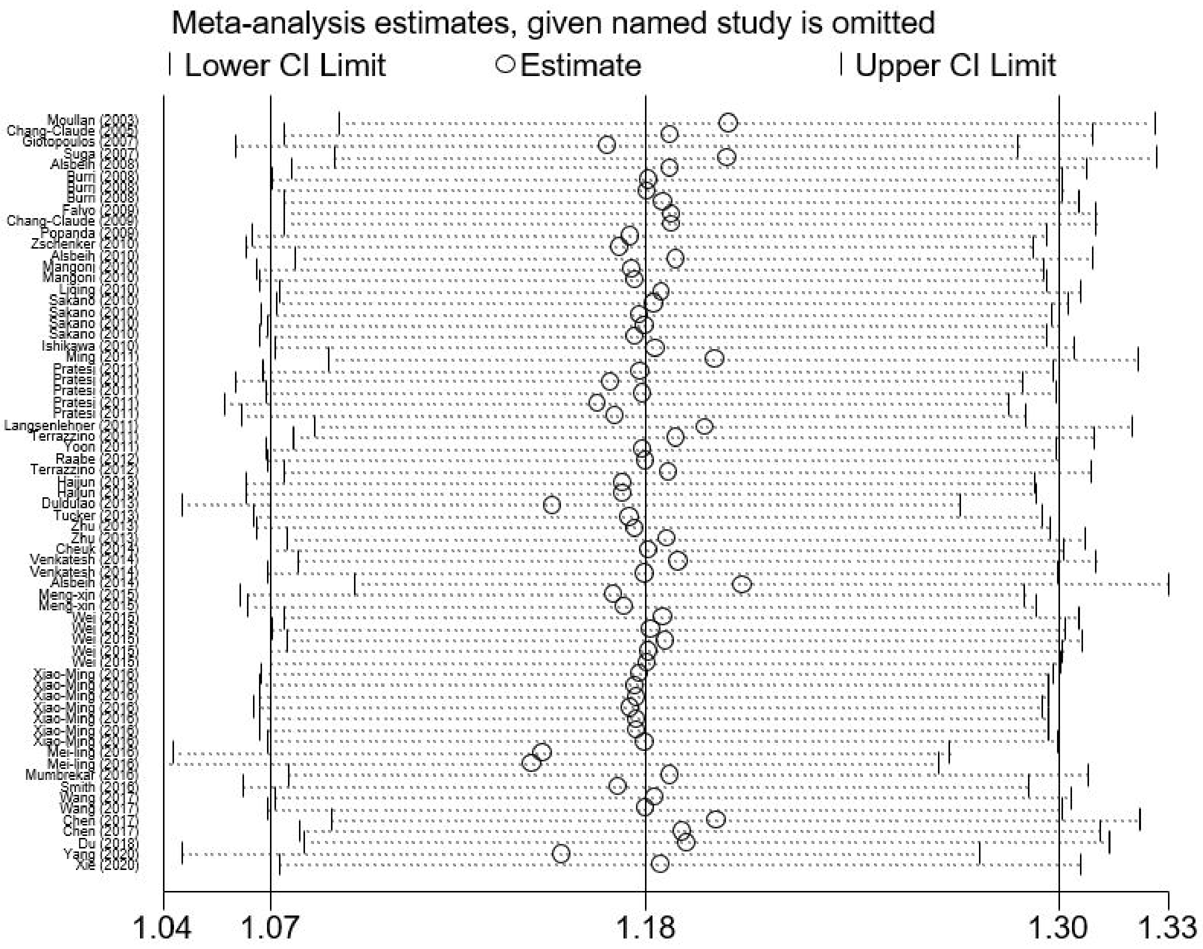

Meta-analysis estimates, given named study is omitted
Lower CI Limit
Estimate
Upper CI Limit
Moullan (2003)
Chang-Claude (2005)
Giotopoulos (2007)
Suga (2007)
Alsbeih (2008)
Burri (2008)
Burri (2008)
Burri (2008)
Falvo (2009)
Chang-Claude (2009)
Popanda (2009)
Zschenker (2010)
Alsbeih (2010)
Mangoni (2010)
Mangoni (2010)
Liqing (2010)
Sakano (2010)
Sakano (2010)
Sakano (2010)
Sakano (2010)
Ishikawa (2010)
Ming (2011)
Pratesi (2011)
Pratesi (2011)
Pratesi (2011)
Pratesi (2011)
Pratesi (2011)
Langsenlehner (2011)
Terrazzino (2011)
Yoon (2011)
Raabe (2012)
Terrazzino (2012)
Haijun (2013)
Haijun (2013)
Duldulao (2013)
Tucker (2013)
Zhu (2013)
Zhu (2013)
Cheuk (2014)
Venkatesh (2014)
Venkatesh (2014)
Alsbeih (2014)
Meng-xin (2015)
Meng-xin (2015)
Wei (2015)
Wei (2015)
Wei (2015)
Wei (2015)
Wei (2015)
Xiao-Ming (2016)
Xiao-Ming (2016)
Xiao-Ming (2016)
Xiao-Ming (2016)
Xiao-Ming (2016)
Xiao-Ming (2016)
Xiao-Ming (2016)
Mei-ling (2016)
Mei-ling (2016)
Mumbrekar (2016)
Smith (2016)
Wang (2017)
Wang (2017)
Chen (2017)
Chen (2017)
Du (2018)
Yang (2020)
Xie (2020)
1.04
1.07
1.18
1.30
1.33

Supplementary File 8

Publication bias of association between XRCC1 expression and treatment response/overall survival.

Publication bias of association between XRCC1 expression and treatment response

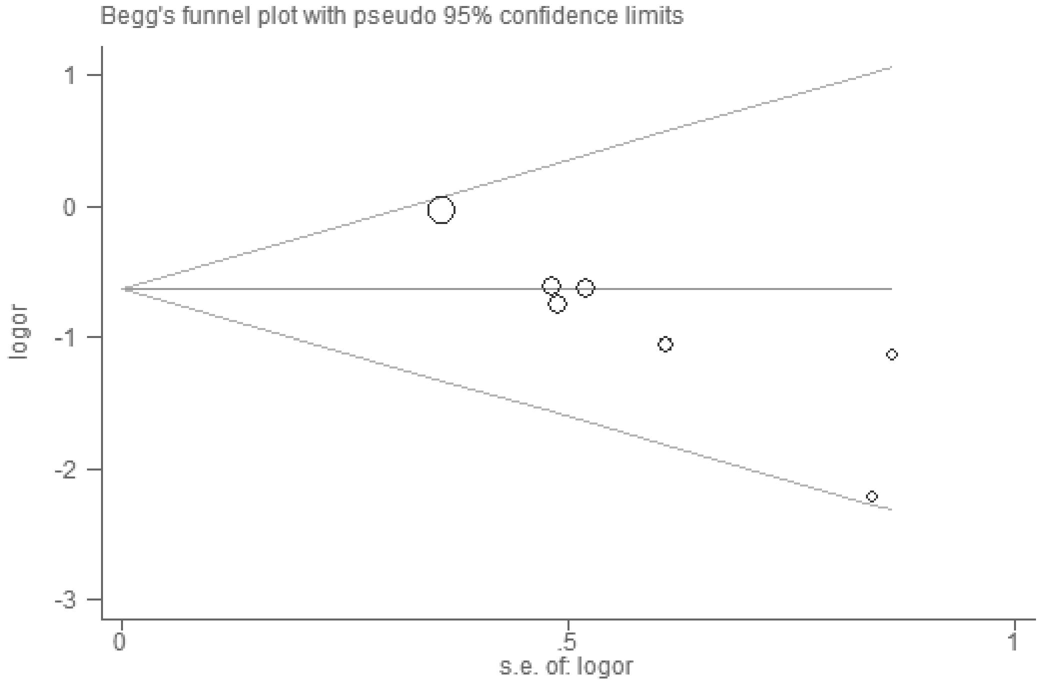

Publication bias of association between XRCC1 expression and overall survival

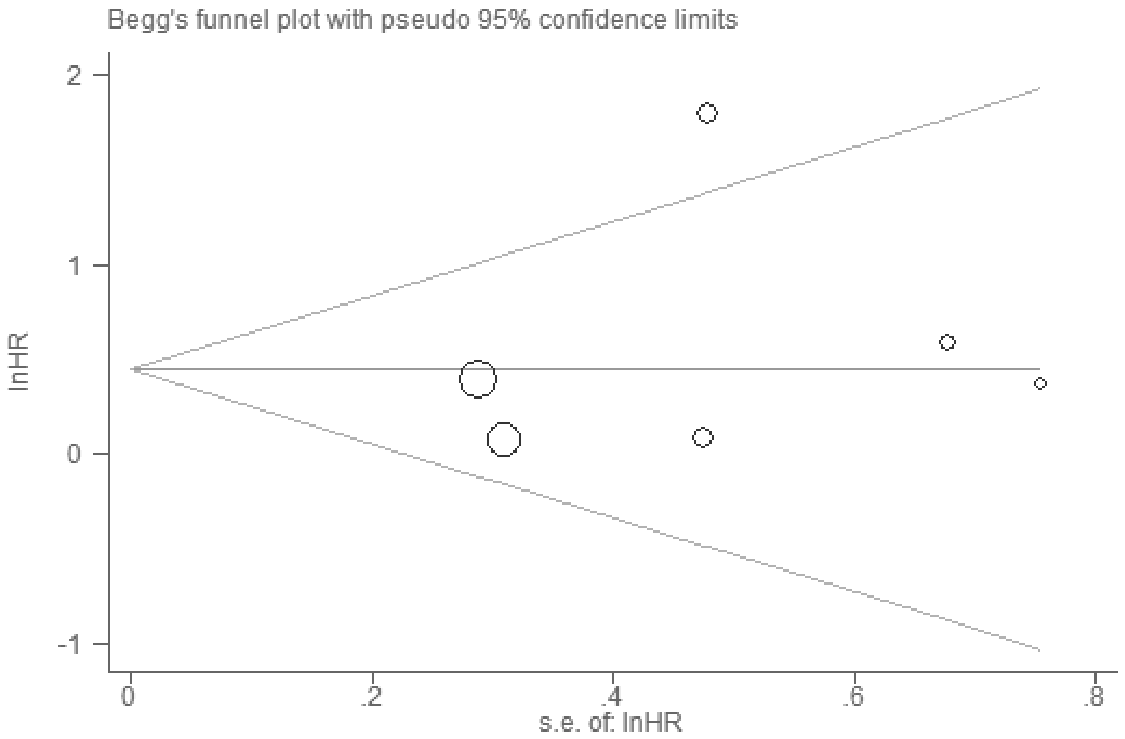

Supplementary File 9

Publication bias of association between XRCC1 rs25487 and treatment response.

AA VS GG

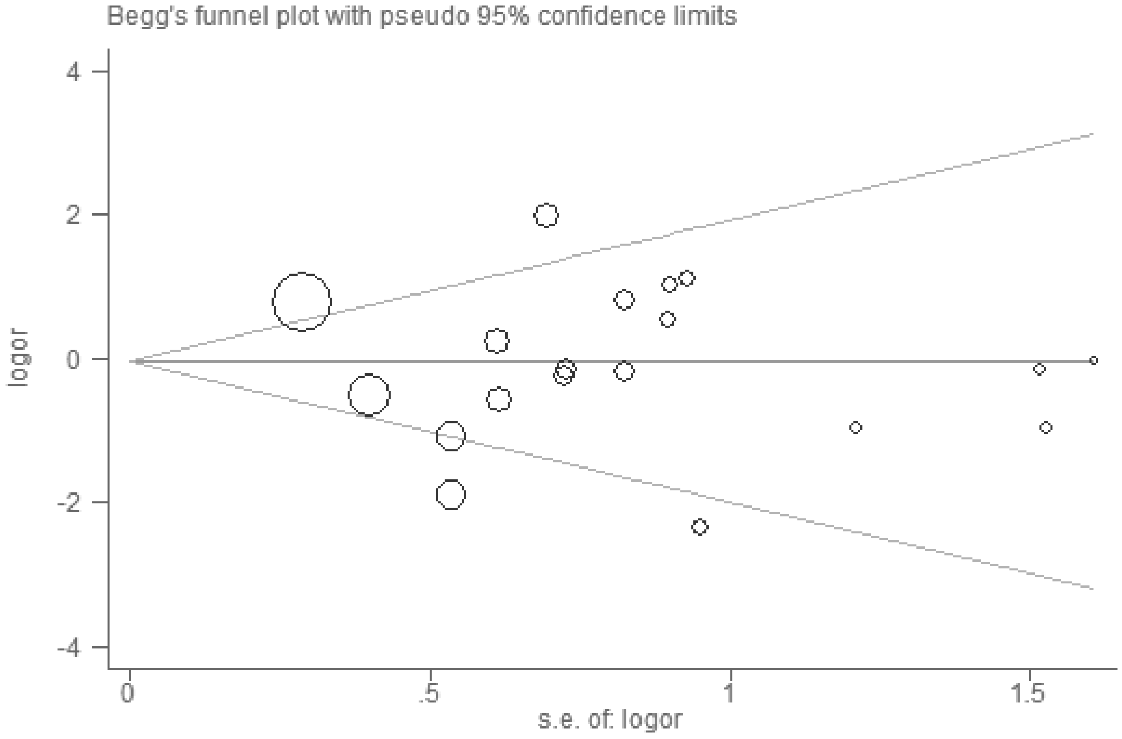

Begg's funnel plot with pseudo 95% confidence limits
4
2
0
-2
-4
logor
0
.5
1
1.5
s.e. of: logor

GA VS GG

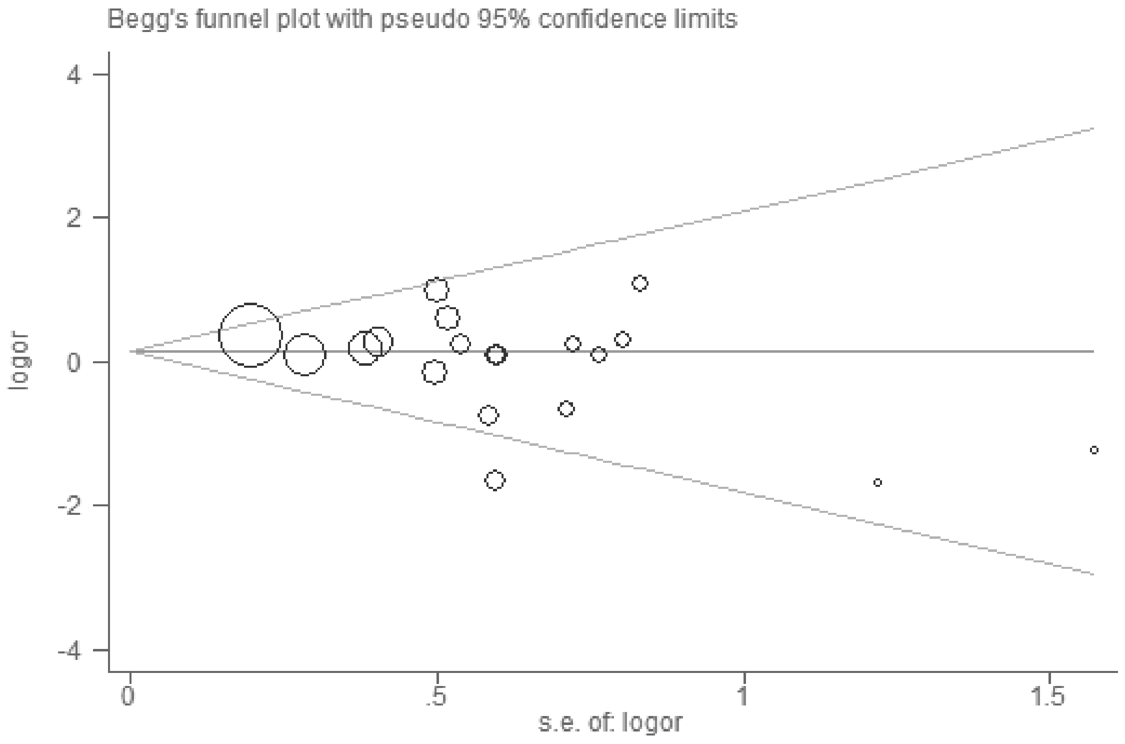

Begg's funnel plot with pseudo 95% confidence limits
4
2
0
-2
-4
logor
0
.5
1
1.5
s.e. of: logor

GA+AA VS GG

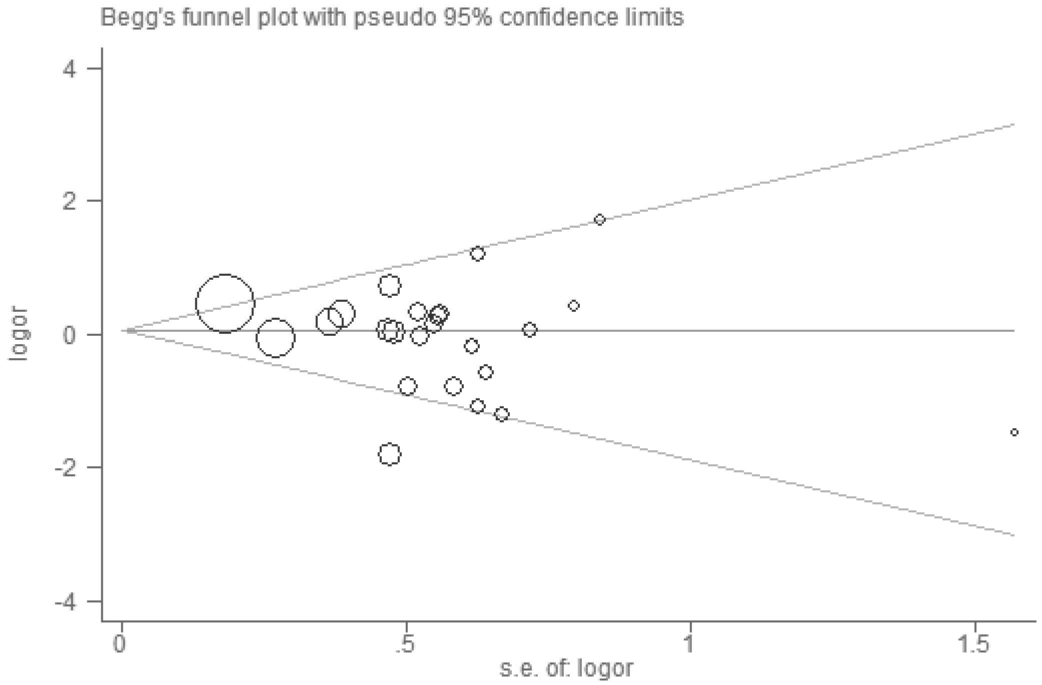

Begg's funnel plot with pseudo 95% confidence limits
4
2
0
-2
-4
logor
0
.5
1
1.5
s.e. of: logor

Supplementary File 10

Publication bias of association between XRCC1 rs25487 and side effects.

AA VS GG

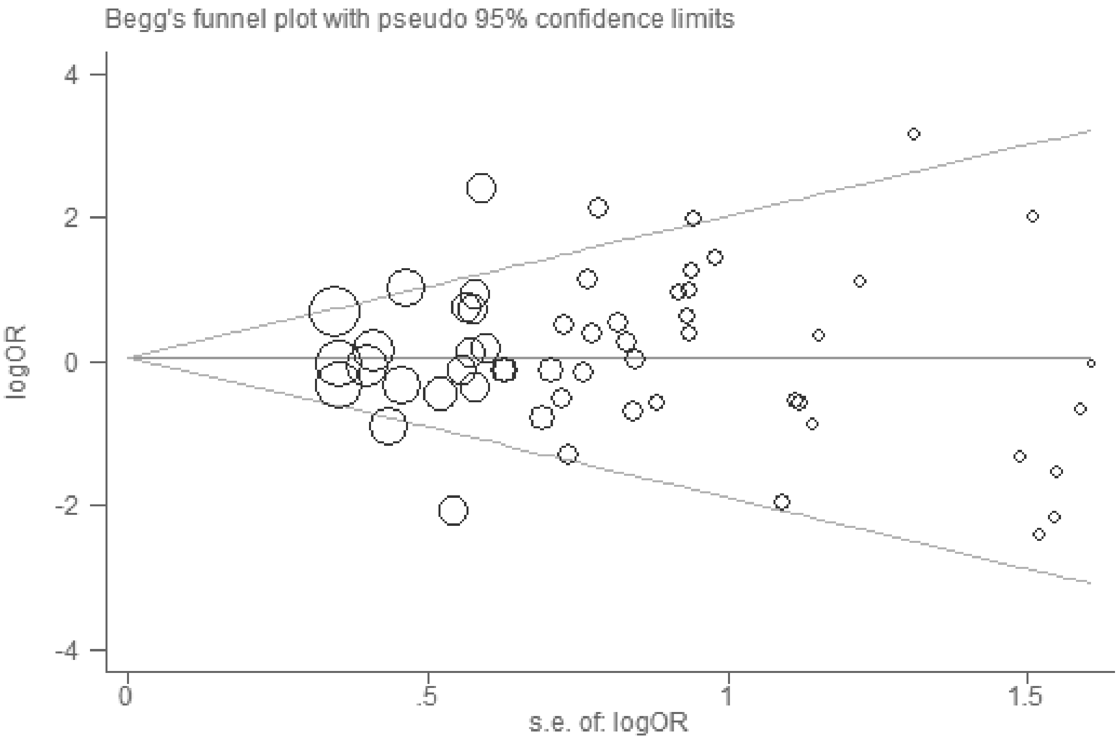

Begg's funnel plot with pseudo 95% confidence limits
logOR
s.e. of: logOR
4
2
0
-2
-4
0
.5
1
1.5

GA VS GG

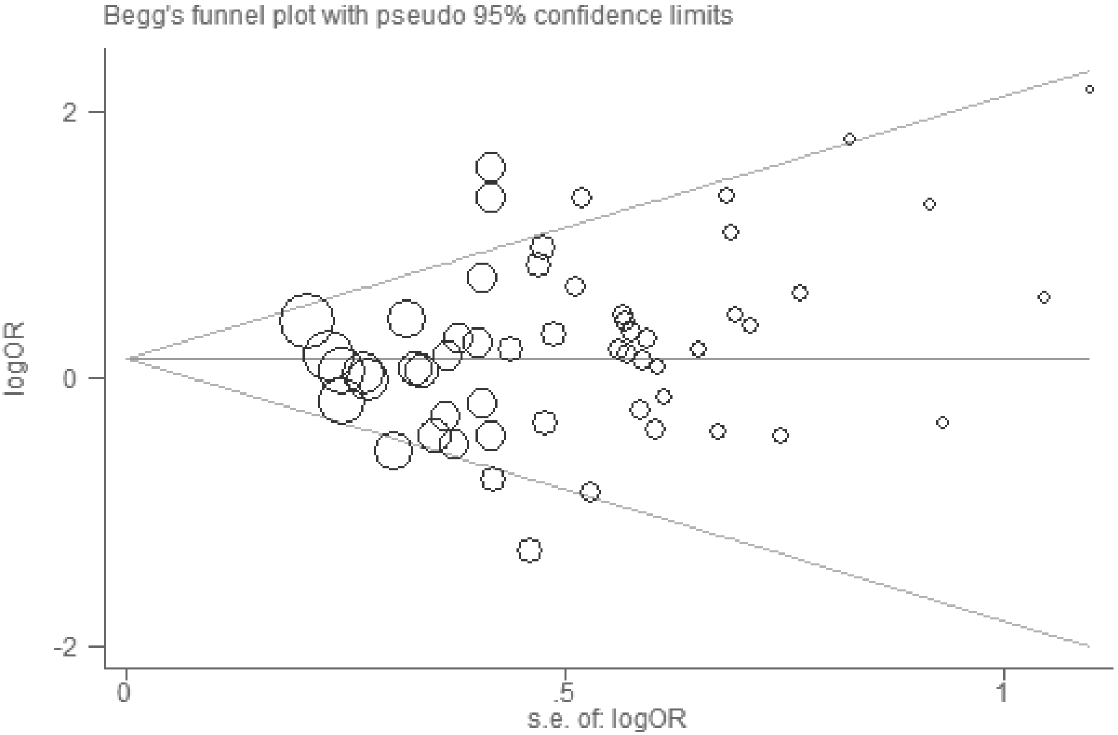

Begg's funnel plot with pseudo 95% confidence limits
2
0
-2
logOR
0
.5
1
s.e. of: logOR

GA+AA VS GG

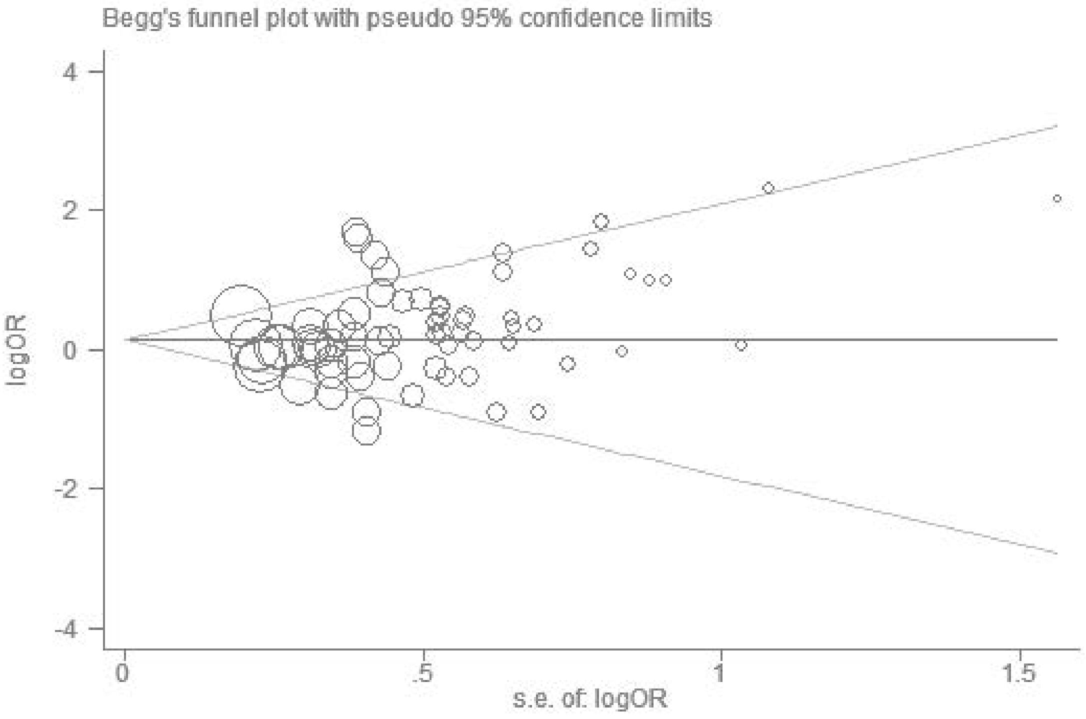

Begg's funnel plot with pseudo 95% confidence limits
4
2
0
-2
-4
logOR
0
.5
1
1.5
s.e. of: logOR

Supplement File 11

Supplement Table 2a. Characteristics of studies with adjusted data associated with rs25487 and side effects

Study	GA VS GG		AA VS GG		GA+AA VS GG		Adjusted factors
	OR (adjusted) 95%CI	OR (crude) 95%CI	OR (adjusted) 95%CI	OR (crude) 95%CI	OR (adjusted) 95%CI	OR (crude) 95%CI	
Moullan (2003)	1.80(0.97-3.31)	0.58 (0.32-1.06)	1.43(0.57-3.54)	0.72 (0.30-1.76)	NA	NA	age
Chang-Claude (2009)	1.09(0.65-1.82)	1.04 (0.61-1.76)	0.63(0.29-1.37)	0.95 (0.43-2.07)	0.96(0.59-1.57)	1.02 (0.62-1.68)	normalised total dose, age at the time of late toxicities evaluation, time since radiotherapy (months), clinic, acute skin toxicity, high blood pressure, allergy, pack-years (never, ≤20, > 20), skin type (always/moderate/seldom sunburn), clinic, marital status(single/divorced/widowed, married/partner).
Popanda (2009)	1.95(0.98-3.87)	1.56 (0.83-2.92)	0.51(0.13-1.97)	0.70 (0.22-2.17)	1.59(0.82-3.10)	1.38 (0.75-2.54)	age, size, year of diagnosis, time between diagnosis and start of radiotherapy, total dose, hormone therapy, smoking and alcohol consumption
Liqing (2010)	1.16 (0.47-3.59)	1.05 (0.54-2.03)	NA	NA	1.06 (0.45-2.53)	1.04 (0.42-2.63)	dose of radiotherapy, age, smoking status, estrogen receptor (ER) status, and progesterone receptor (PR) status
Sakano (2010)	NA	NA	NA	NA	1.2(0.22-5.80)	0.80 (0.19-3.43)	total cisplatin dose and total radiation dose
Sakano (2010)	NA	NA	NA	NA	2.30(0.68-8.40)	1.54 (0.51-4.68)	total cisplatin dose and total radiation dose
Sakano (2010)	NA	NA	NA	NA	1.70(0.55-5.00)	1.25 (0.45-3.47)	total cisplatin dose and total radiation dose

Terrazzino (2011)	NA	NA	NA	NA	0.84(0.38-1.90)	0.83 (0.42-1.63)	age, body mass index, breast diameter, follow-up, adjuvant treatment, history of vasculopathy, smoking status, dose per fraction, radiation quality, and boost method
Mei-ling (2016)	4.11(1.75-9.70)	3.83(1.70-8.65)	9.10(2.71-0.57)	11.00(3.46-.99)	NA	NA	surgery, dose of radiotherapy
Mei-ling (2016)	5.01(2.17-1.58)	4.84(2.14-0.94)	7.17(1.51-4.09)	8.42(1.81-9.11)	NA	NA	surgery, dose of radiotherapy

NA: not available

Supplement Table 2b. Characteristics of studies with adjusted data associated with rs25487 and treatment response

Study	GA VS GG		AA VS GG		GA+AA VS GG		Adjusted factors
	OR (adjusted) 95%CI	OR (crude) 95%CI	OR (adjusted) 95%CI	OR (crude) 95%CI	OR (adjusted) 95%CI	OR (crude) 95%CI	
Qing-hua (2007)	1.34(0.55-3.26)	1.31 (0.59-2.89)	1.69(0.27-10.79)	1.71 (0.29-9.89)	1.23(0.52-2.89)	1.36 (0.64-2.89)	gender, age, pathology type, pack-years, stage
Xiao-mei (2014)	1.43(0.34-6.03)	1.26 (0.44-3.63)	13.5(0.95-92.49)	2.25(0.45-1.33)	NA	NA	age, stage, tumor size(≤4cm, ＞4cm), radiation dose at point A, genotype

NA: not available

Supplement File 12

Forest plots of adjusted data and crude data of overall analyses for association between rs25487 and side effects/treatment response

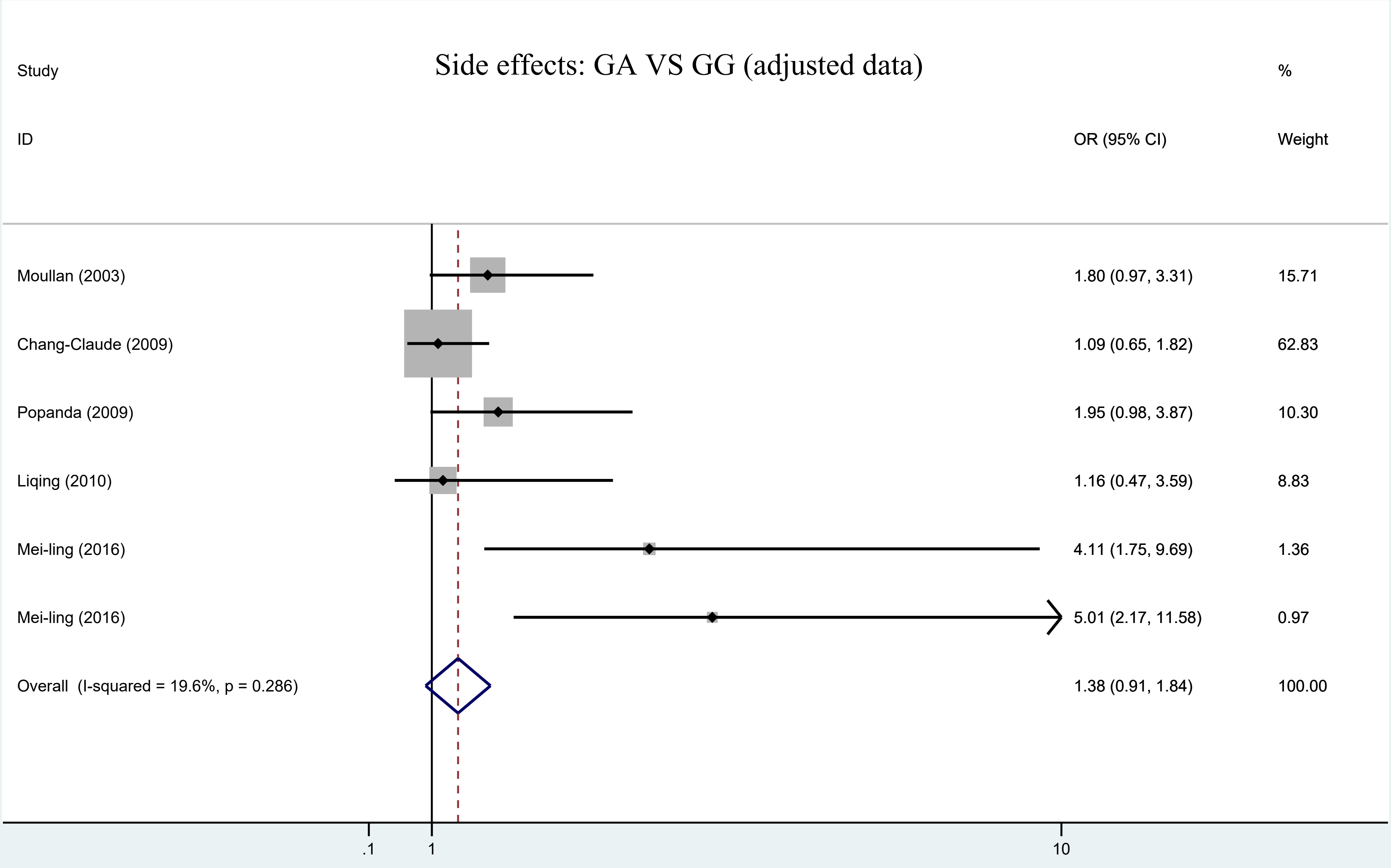
Side effects: GA VS GG (adjusted data)
Study
ID
OR (95% CI)
%
Weight
Moullan (2003)
1.80 (0.97, 3.31)
15.71
Chang-Claude (2009)
1.09 (0.65, 1.82)
62.83
Popanda (2009)
1.95 (0.98, 3.87)
10.30
Liqing (2010)
1.16 (0.47, 3.59)
8.83
Mei-ling (2016)
4.11 (1.75, 9.69)
1.36
Mei-ling (2016)
5.01 (2.17, 11.58)
0.97
Overall (I-squared = 19.6%, p = 0.286)
1.38 (0.91, 1.84)
100.00
.1
1
10

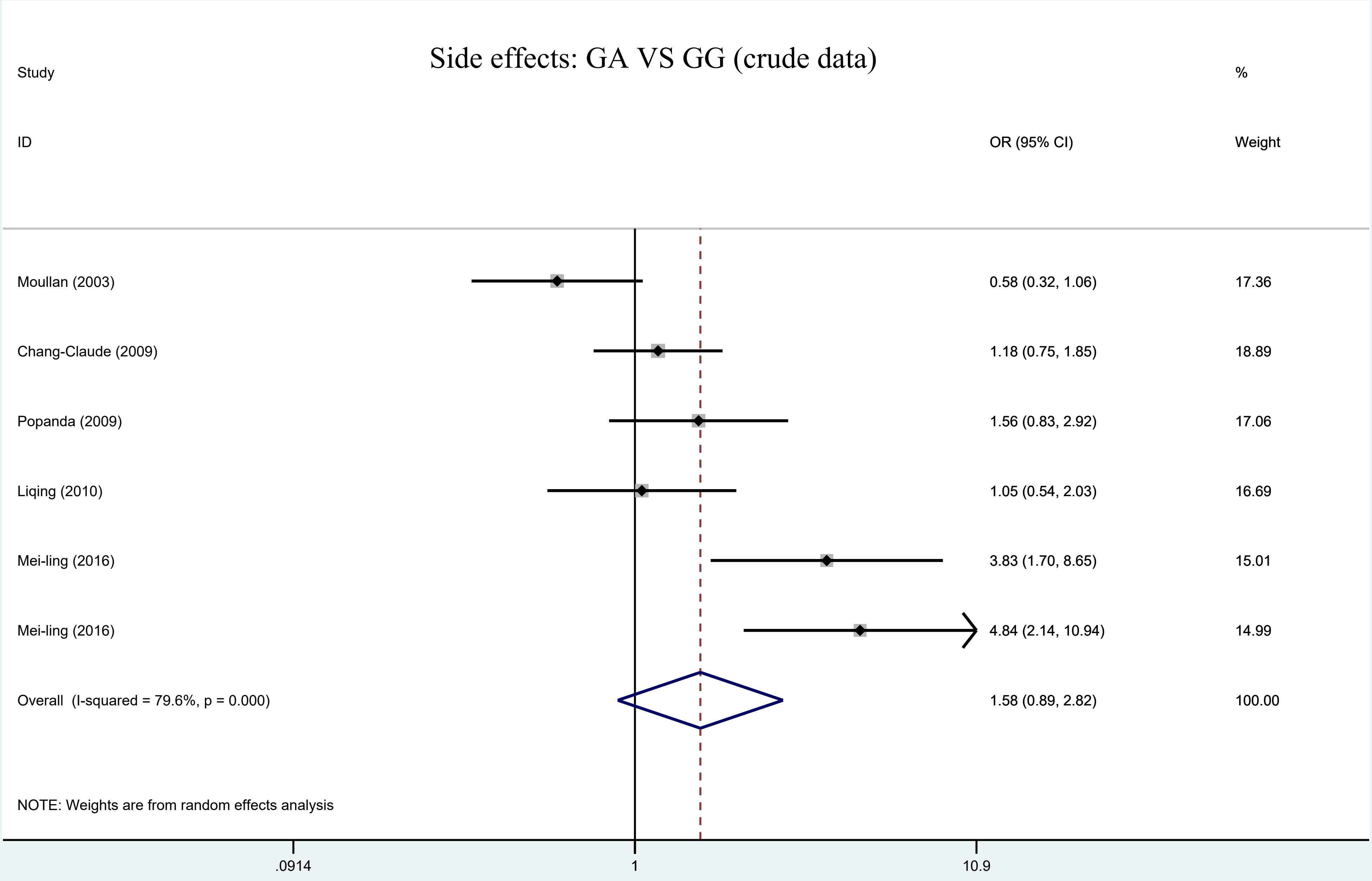

Side effects: GA VS GG (crude data)
Study
ID
OR (95% CI)
%
Weight
Moullan (2003)
0.58 (0.32, 1.06)
17.36
Chang-Claude (2009)
1.18 (0.75, 1.85)
18.89
Popanda (2009)
1.56 (0.83, 2.92)
17.06
Liqing (2010)
1.05 (0.54, 2.03)
16.69
Mei-ling (2016)
3.83 (1.70, 8.65)
15.01
Mei-ling (2016)
4.84 (2.14, 10.94)
14.99
Overall (I-squared = 79.6%, p = 0.000)
1.58 (0.89, 2.82)
100.00
NOTE: Weights are from random effects analysis
.0914
1
10.9

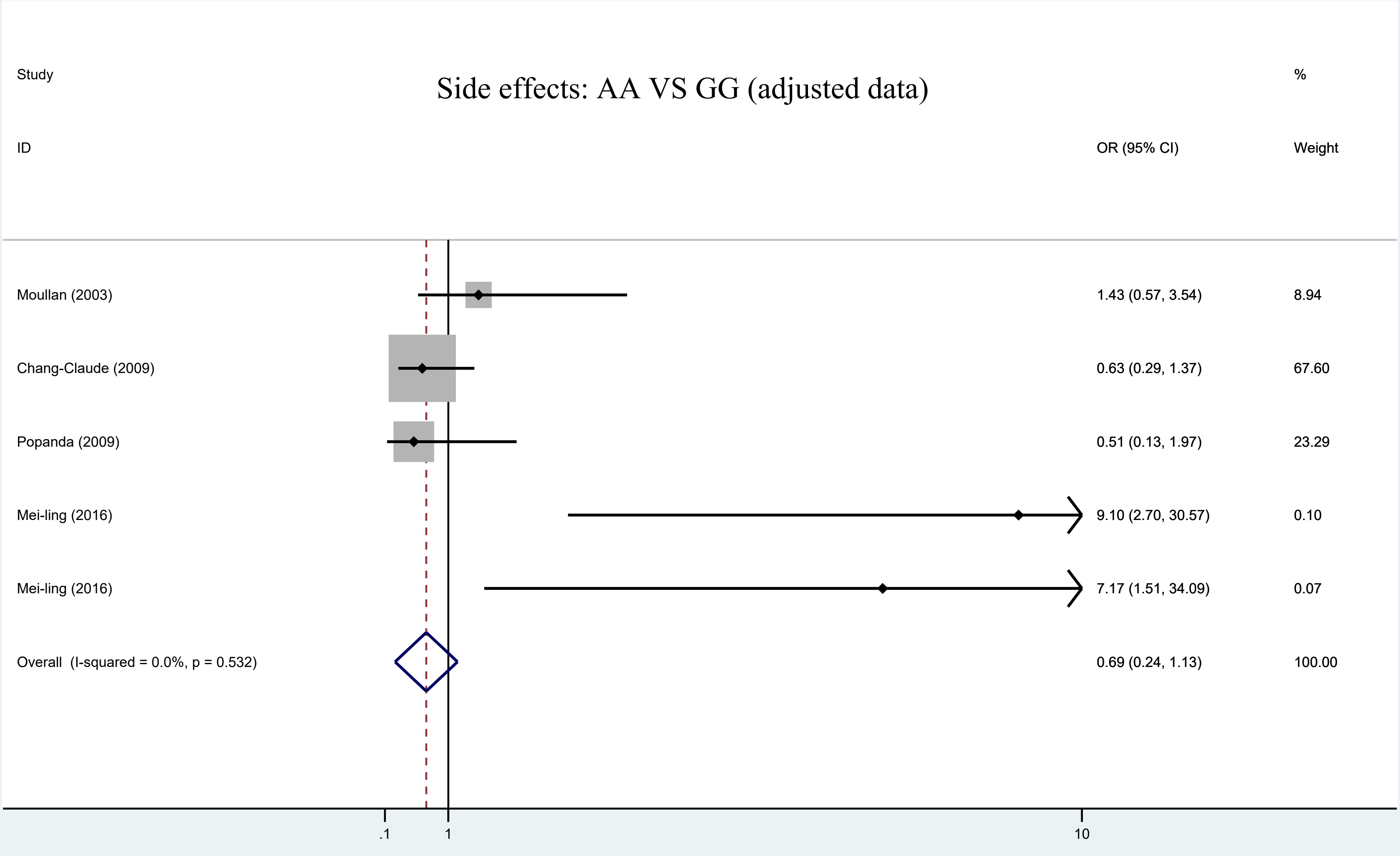
Study
Side effects: AA VS GG (adjusted data)
%
ID
OR (95% CI)
Weight
Moullan (2003)
1.43 (0.57, 3.54)
8.94
Chang-Claude (2009)
0.63 (0.29, 1.37)
67.60
Popanda (2009)
0.51 (0.13, 1.97)
23.29
Mei-ling (2016)
9.10 (2.70, 30.57)
0.10
Mei-ling (2016)
7.17 (1.51, 34.09)
0.07
Overall (I-squared = 0.0%, p = 0.532)
0.69 (0.24, 1.13)
100.00
.1
1
10

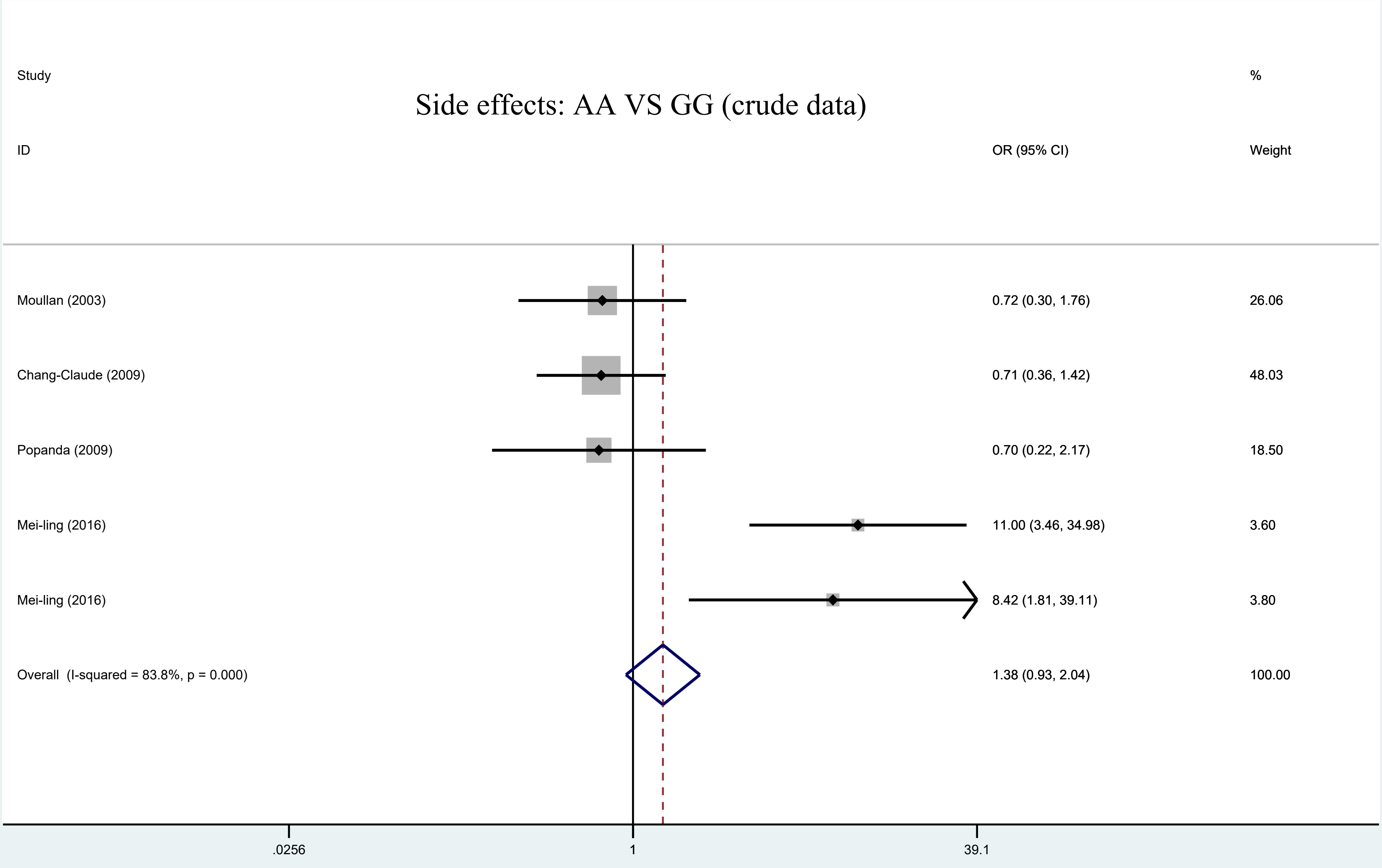
Side effects: AA VS GG (crude data)
Study
ID
OR (95% CI)
%
Weight
Moullan (2003)
0.72 (0.30, 1.76)
26.06
Chang-Claude (2009)
0.71 (0.36, 1.42)
48.03
Popanda (2009)
0.70 (0.22, 2.17)
18.50
Mei-ling (2016)
11.00 (3.46, 34.98)
3.60
Mei-ling (2016)
8.42 (1.81, 39.11)
3.80
Overall (I-squared = 83.8%, p = 0.000)
1.38 (0.93, 2.04)
100.00
.0256
1
39.1

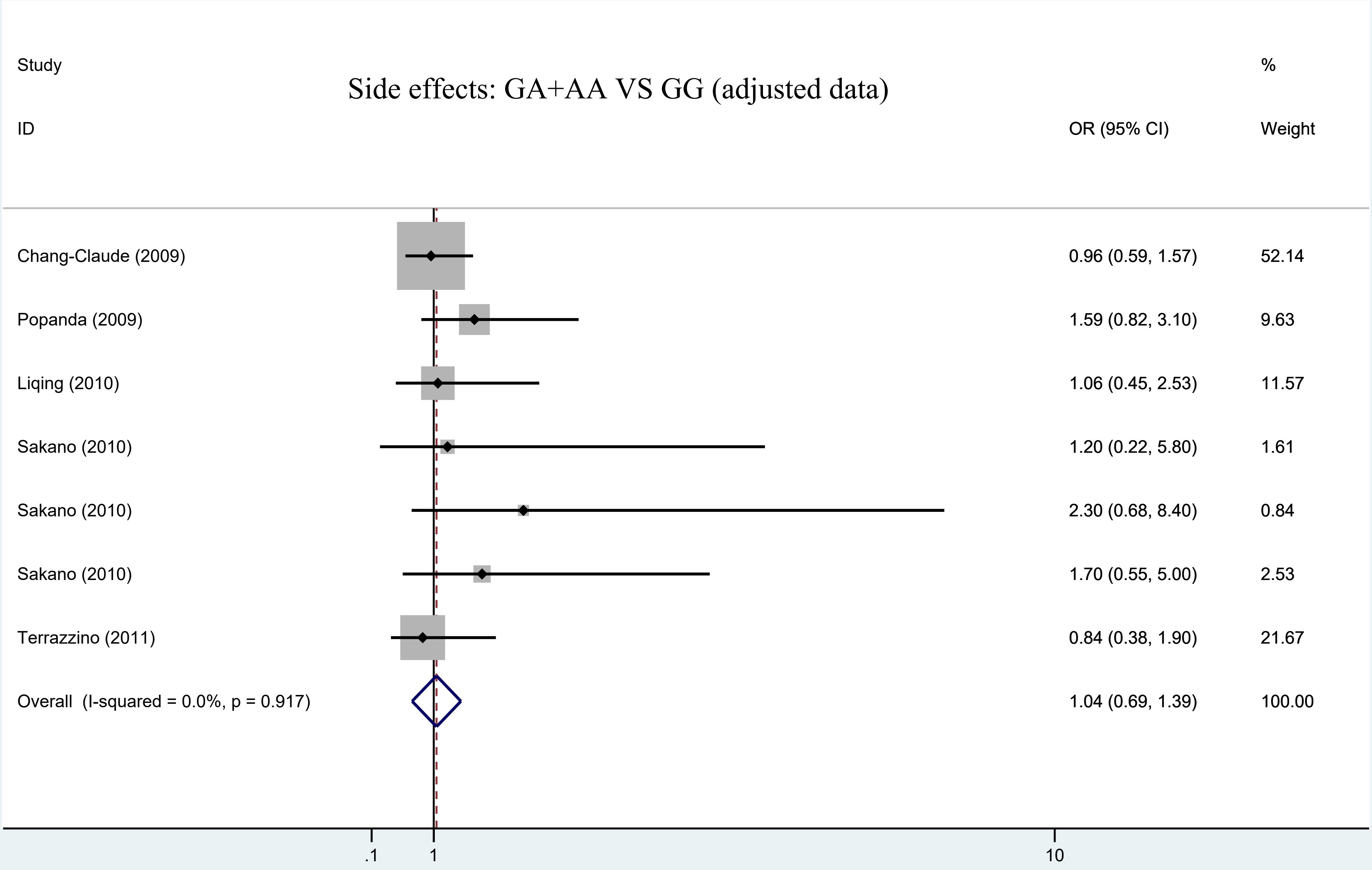
Study
Side effects: GA+AA VS GG (adjusted data)
%
ID
OR (95% CI)
Weight
Chang-Claude (2009)
0.96 (0.59, 1.57)
52.14
Popanda (2009)
1.59 (0.82, 3.10)
9.63
Liqing (2010)
1.06 (0.45, 2.53)
11.57
Sakano (2010)
1.20 (0.22, 5.80)
1.61
Sakano (2010)
2.30 (0.68, 8.40)
0.84
Sakano (2010)
1.70 (0.55, 5.00)
2.53
Terrazzino (2011)
0.84 (0.38, 1.90)
21.67
Overall (I-squared = 0.0%, p = 0.917)
1.04 (0.69, 1.39)
100.00
.1
1
10

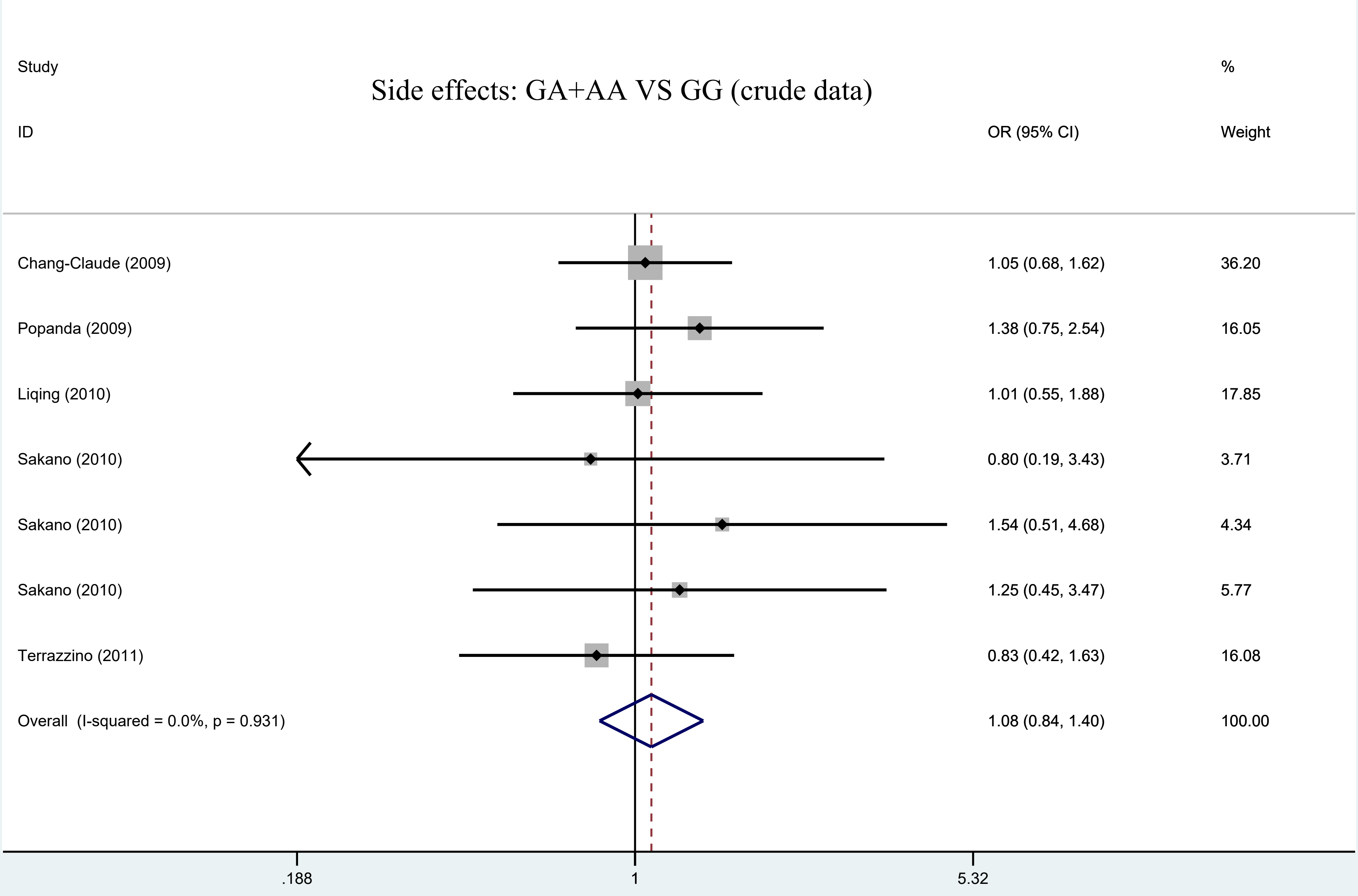

Side effects: GA+AA VS GG (crude data)
Study
ID
OR (95% CI)
%
Weight
Chang-Claude (2009)
1.05 (0.68, 1.62)
36.20
Popanda (2009)
1.38 (0.75, 2.54)
16.05
Liqing (2010)
1.01 (0.55, 1.88)
17.85
Sakano (2010)
0.80 (0.19, 3.43)
3.71
Sakano (2010)
1.54 (0.51, 4.68)
4.34
Sakano (2010)
1.25 (0.45, 3.47)
5.77
Terrazzino (2011)
0.83 (0.42, 1.63)
16.08
Overall (I-squared = 0.0%, p = 0.931)
1.08 (0.84, 1.40)
100.00
.188
1
5.32

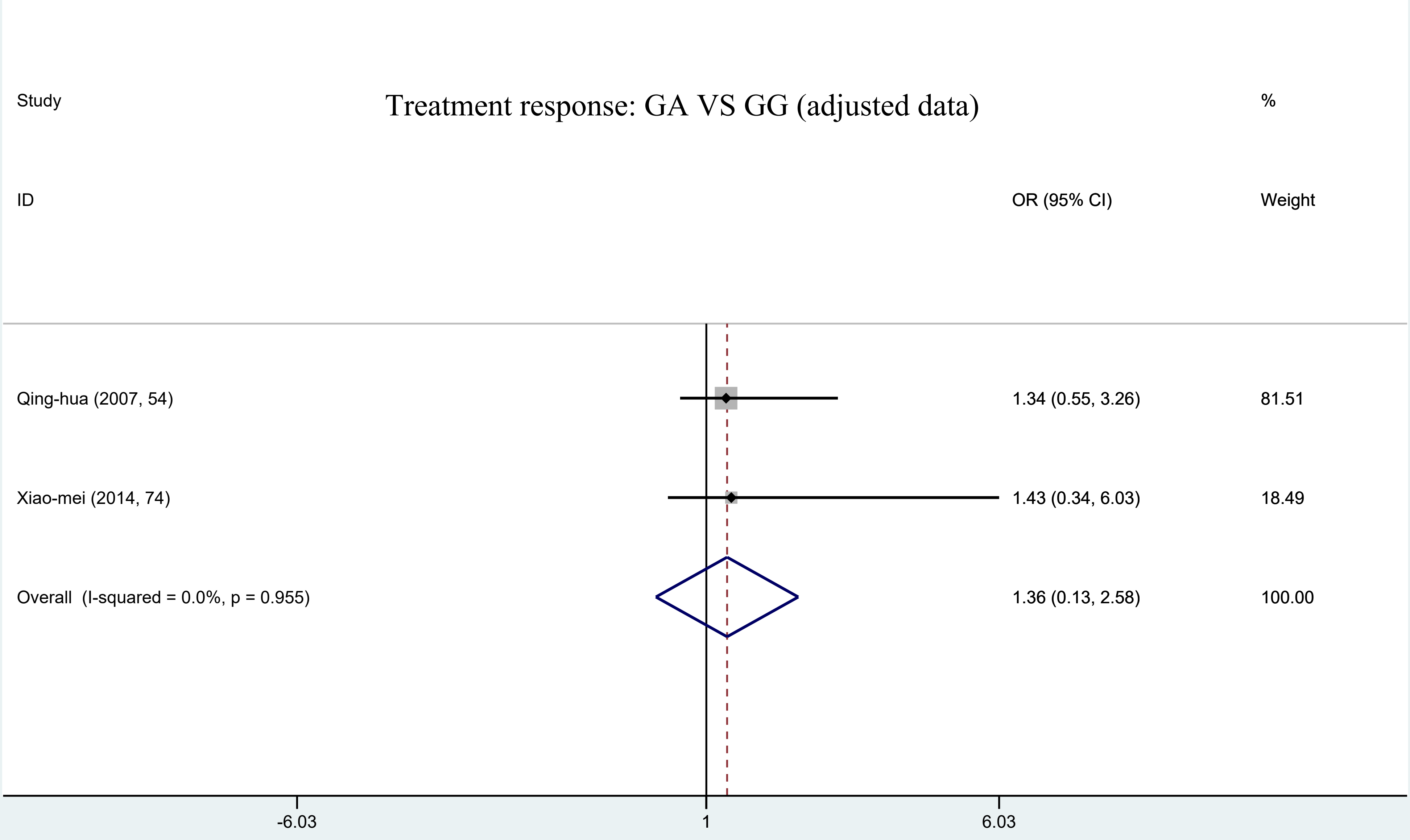
Treatment response: GA VS GG (adjusted data)
Study
ID
%
Weight
OR (95% CI)
Qing-hua (2007, 54)
1.34 (0.55, 3.26)
81.51
Xiao-mei (2014, 74)
1.43 (0.34, 6.03)
18.49
Overall (I-squared = 0.0%, p = 0.955)
1.36 (0.13, 2.58)
100.00
-6.03
1
6.03

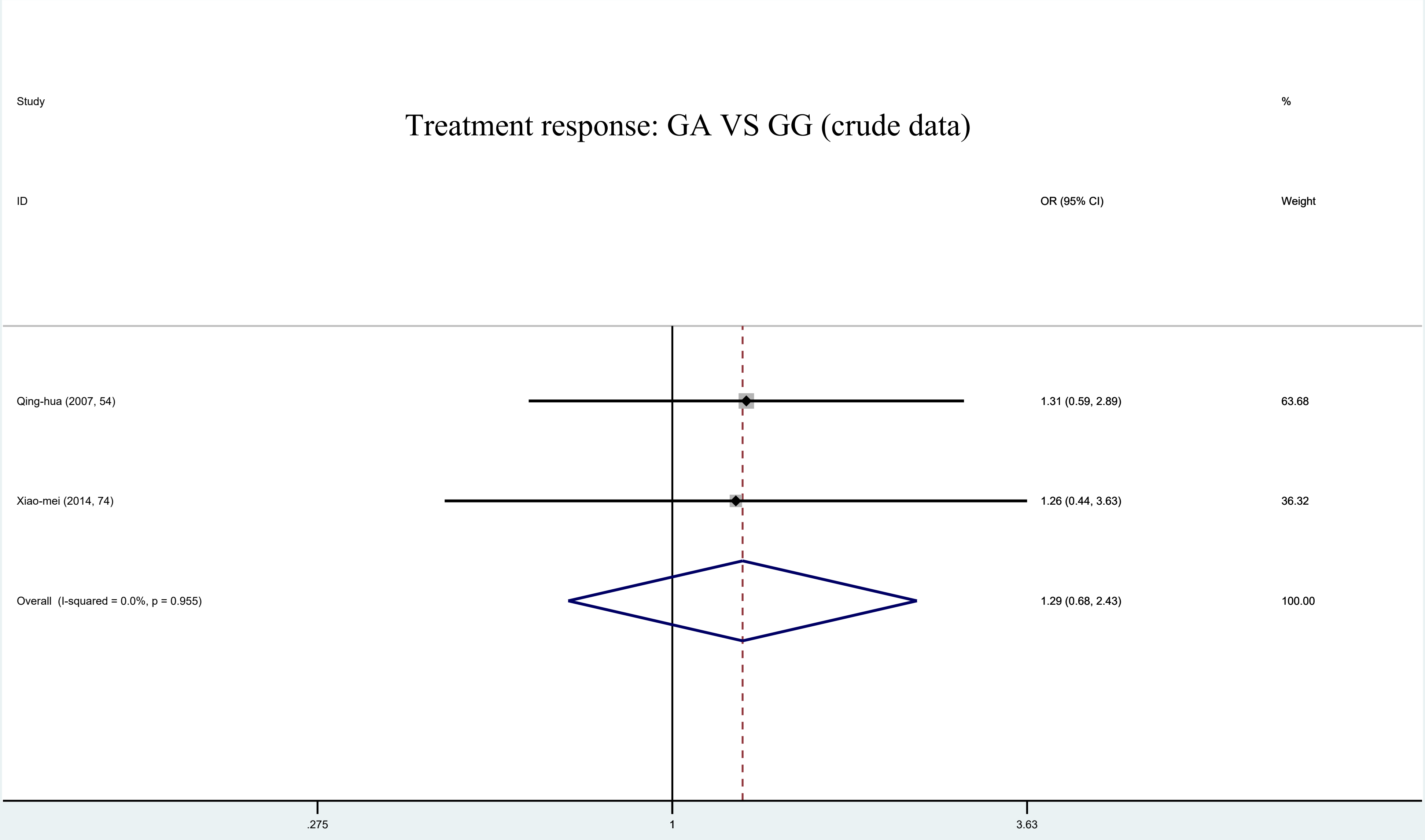

Study
Treatment response: GA VS GG (crude data)
%
ID
OR (95% CI)
Weight
Qing-hua (2007, 54)
1.31 (0.59, 2.89)
63.68
Xiao-mei (2014, 74)
1.26 (0.44, 3.63)
36.32
Overall (I-squared = 0.0%, p = 0.955)
1.29 (0.68, 2.43)
100.00
.275
1
3.63

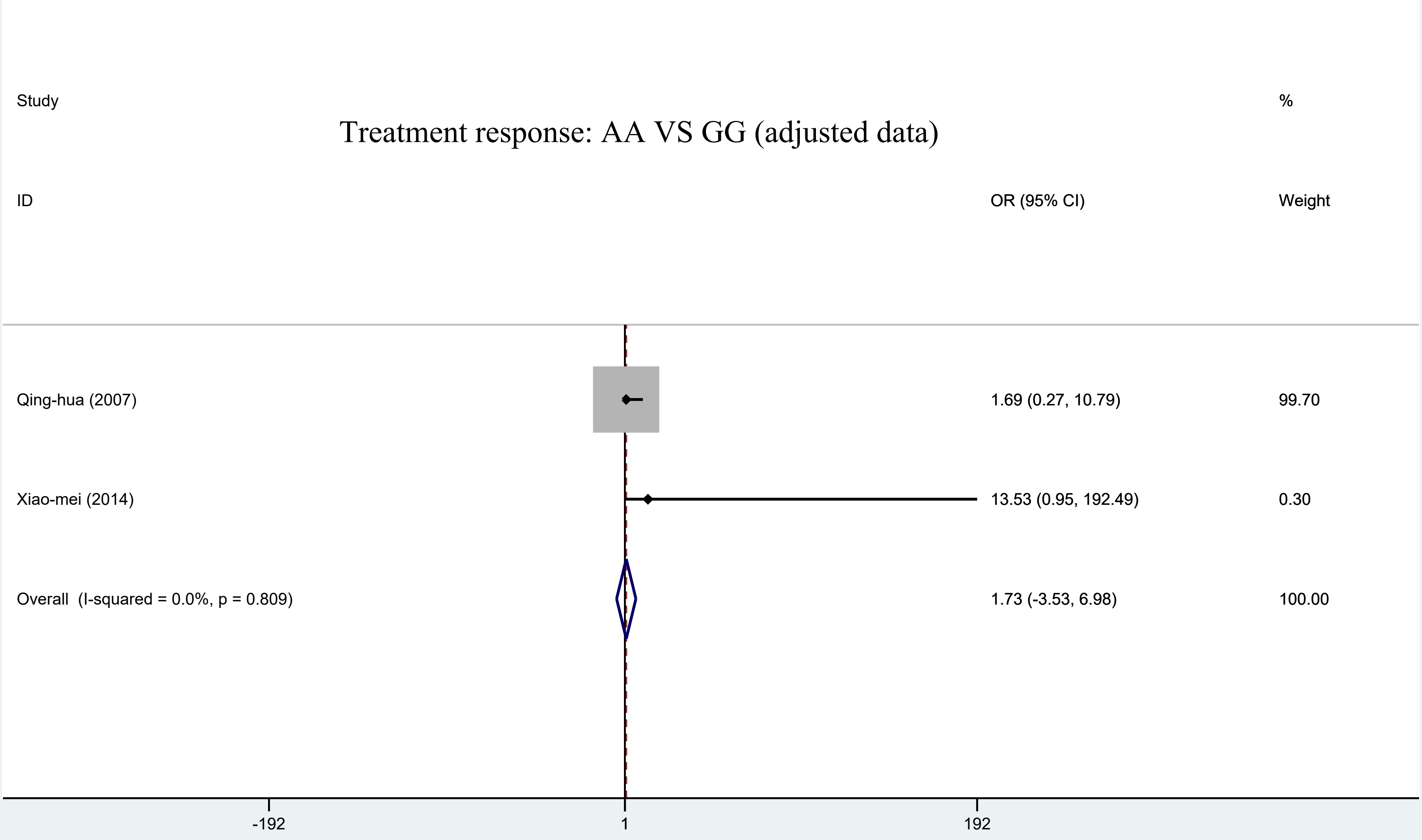
Treatment response: AA VS GG (adjusted data)
Study
ID
%
Weight
OR (95% CI)
Qing-hua (2007)
1.69 (0.27, 10.79)
99.70
Xiao-mei (2014)
13.53 (0.95, 192.49)
0.30
Overall (I-squared = 0.0%, p = 0.809)
1.73 (-3.53, 6.98)
100.00
-192
1
192

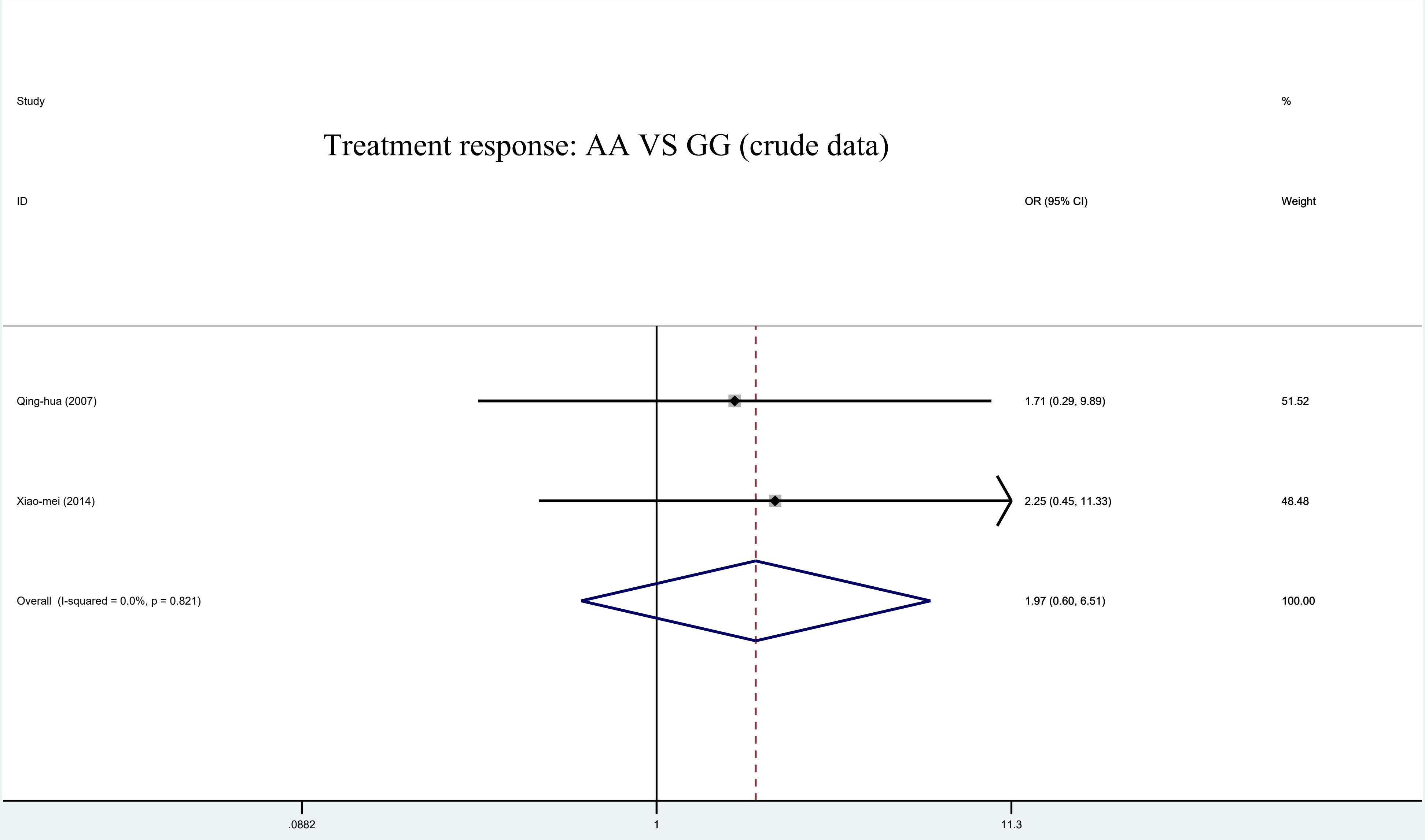

Study
%
Treatment response: AA VS GG (crude data)
ID
OR (95% CI)
Weight
Qing-hua (2007)
1.71 (0.29, 9.89)
51.52
Xiao-mei (2014)
2.25 (0.45, 11.33)
48.48
Overall (I-squared = 0.0%, p = 0.821)
1.97 (0.60, 6.51)
100.00
.0882
1
11.3
